# Supplementary material for: Insights into 6‐Methylsalicylic Acid Bio‐assembly by Using Chemical Probes
Source: Angew Chem Int Ed Engl. 2016 Feb 2;55(10):3463–7. doi: 10.1002/anie.201509038 (PMC4797705; doi:10.1002/anie.201509038)
Supplement: Supplementary file 1 — Supplementary [file ANIE-55-3463-s001.pdf]

Angewandte Chemie
Eine Zeitschrift der Gesellschaft Deutscher Chemiker

Supporting Information

Insights into 6-Methylsalicylic Acid Bio-assembly by Using Chemical Probes

*James S. Parascandolo, Judith Havemann, Helen K. Potter, Fanglu Huang, Elena Riva, Jack Connolly, Ina Wilkening, Lijiang Song, Peter F. Leadlay, and Manuela Tosin**

anie_201509038_sm_miscellaneous_information.pdf

Contents

1 SYNTHESIS OF CHEMICAL PROBES 3

1.1 GENERAL METHODS 3

1.2 SYNTHESIS OF PROBE 5 (BUTYL 6-ACETAMIDO-3-OXOHEXANOATE) 3

1.3 SYNTHESIS OF TRIKETIDE SUBSTRATE 13 (*N*-(6-HYDROXY-4,8-DIOXO-NONYL)ACETAMIDE) 4

1.3.1 METHYL 2-(2-(3-ACETAMIDOPROPYL)-4-PHENYL-1,3-DIOXOLAN-2-YL)ACETATE (17) 4

1.3.2 *N*-(3-(2-(2-HYDROXYETHYL)-4-PHENYL-1,3-DIOXOLAN-2-YL)PROPYL)ACETAMIDE (18) 5

1.3.3 *N*-[3-[2-(2-OXOETHYL)-4-PHENYL-1,3-DIOXOLAN-2-YL]PROPYL]ACETAMIDE (19) 6

1.3.4 *N*-(6-HYDROXY-4,8-DIOXO-NONYL)ACETAMIDE (13) 6

1.4 SYNTHESIS OF 6-MSA *N*-ACETYLCYSTEAMINE THIOESTER (14) 7

1.5 SYNTHESIS OF 6-MSA *N*-DECANOYLCYSTEAMINE THIOESTER (15) 8

1.5.1 SYNTHESIS OF *N*, *N'*-DIDECANOYLCYSTAMINE (23) 8

1.5.2 SYNTHESIS OF *N*-DECANOYLCYSTEAMINE (24) 9

1.5.3 SYNTHESIS OF 6-MSA *N*-DECANOYLCYSTEAMINE THIOESTER (15) 9

2 *IN VIVO* EXPERIMENTS 11

2.1 MICROBIOLOGY METHODS 11

2.2 GROWTH OF *PENICILLIUM PATULUM*, *ESCHERICHIA COLI* BAP1 PKOS007-109, *STREPTOMYCES ANTIBIOTICUS* DSM40725 STRAINS AND MASS SPECTROMETRY ANALYSIS 11

2.3 SUMMARY OF 6-MSA INTERMEDIATES CAPTURED FROM *P. PATULUM*, *E. COLI* BAP1 PKOS007-109 AND *S. ANTIBIOTICUS* DSM40725 14

2.4 CAPTURE OF 6-MSAS INTERMEDIATES FROM *P. PATULUM VIA* CHAIN TERMINATION PROBES 18

2.4.1 INTERMEDIATE CAPTURE BY PROBES 4A-B 18

2.4.2 INTERMEDIATE CAPTURE BY PROBE 5 (BUTYL 6-ACETAMIDO-3-OXOHEXANOATE) 19

2.4.3 INTERMEDIATE CAPTURE BY PROBE 6 (METHYL 3-OXO-6-(PENT-4-YNAMIDO) HEXANOATE) 24

2.4.4 INTERMEDIATE CAPTURE BY PROBE 8 (METHYL 6-DECANAMIDO-3-OXOHEXANOATE) 26

2.5 CAPTURE OF 6-MSAS INTERMEDIATES FROM *E. COLI* BAP1 PKOS007-109 *VIA* CHAIN TERMINATION PROBES 33

2.5.1 INTERMEDIATE CAPTURE BY PROBE 4A 33

2.5.2 INTERMEDIATE CAPTURE BY PROBE 6 34

2.5.3 INTERMEDIATE CAPTURE BY PROBE 8 35

2.6 CAPTURE OF 6-MSAS FROM *S. ANTIBIOTICUS* DSM40725 *VIA* CHAIN TERMINATION PROBES 42

2.6.1 INTERMEDIATE CAPTURE BY PROBE 8 42

2.6.2 INTERMEDIATE CAPTURE BY PROBE 6 48

2.6.3 INTERMEDIATE CAPTURE BY PROBE 7 (METHYL 2-FLUORO-3-OXO-6-(PENT-4-YNAMIDO) HEXANOATE) 51

3 *IN VITRO* EXPERIMENTS 54

3.1 CONSTRUCTION OF *E. COLI* EXPRESSING 6-MSAS AND 6-MSAS H958A 54

3.2 6-MSAS AND 6-MSAS H958A EXPRESSION AND PURIFICATION 54

3.3 6-MSAS AND 6-MSAS H958A ACTIVITY ASSAYS 56

3.3.1 PRODUCTION OF 6-MSA AND TAL 56

3.3.2 KETOREDUCTASE ACTIVITY ASSAY 57

3.3.3 CHAIN TERMINATION ASSAYS 58

3.4 *IN VITRO* CAPTURE OF INTERMEDIATES FROM 6-MSAS AND 6-MSAS H958A *VIA* PROBES 9A-B 59

3.5 ENZYMATIC DEHYDRATION OF *N*-(6-HYDROXY-4,8-DIOXO-NONYL)ACETAMIDE 13 63

3.6 THIOESTER HYDROLASE ASSAYS 65

3.7 COMPARISON OF *P. PATULUM* 6-MSAS TO HOMOLOGUES 67

3.8 NMR SPECTRA OF SYNTHETIC COMPOUNDS 71

3.8.1 ^{1}H- NMR OF BUTYL BUTYL 6-ACETAMIDO-3-OXOHEXANOATE (5) 71

3.8.2 ^{1}H- AND ^{13}C- NMR OF METHYL 2-(2-(3-ACETAMIDOPROPYL)-4-PHENYL-1,3-DIOXOLAN-2-YL)ACETATE (17) 72

3.8.3 ^{1}H- AND ^{13}C- NMR OF N-(3-(2-(2-HYDROXYETHYL)-4-PHENYL-1,3-DIOXOLAN-2-YL)PROPYL)ACETAMIDE (18) 73

3.8.4 ^{1}H- NMR OF *N*-[3-[2-(2-OXOETHYL)-4-PHENYL-1,3-DIOXOLAN-2 YL]PROPYL] ACETAMIDE (19) 74

3.8.5 ^{1}H- AND ^{13}C- NMR OF *N*-(6-HYDROXY-4,8-DIOXO-NONYL)ACETAMIDE (13) **75**

3.8.6 ^{1}H- AND ^{13}C-NMR OF *N*, *N'*-DIDECANOYLCYSTAMINE (23) 76

3.8.7 ^{1}H- AND ^{13}C-NMR OF *N*-DECANOYLCYSTEAMINE (24) 77

3.8.8 ^{1}H- AND ^{13}C-NMR OF 6-MSA *N*-DECANOYLCYSTEAMINE THIOESTER (15) 78

4 REFERENCES 79

1 Synthesis of chemical probes

1.1 General methods

Unless specified otherwise, chemicals were purchased from Sigma Aldrich, Fisher Scientific, Carbosynth and Alfa Aesar and were used without further purification. Anhydrous dichloromethane and toluene were purchased from VWR International (AR grade) and dried using solvent towers. Anhydrous ethyl acetate, isopropanol, butanol, dimethyl sulfoxide and pyridine were purchased from Fisher Scientific. Reagent grade dichloromethane, ethyl acetate, methanol, acetonitrile, cyclohexane, butanol and tetrahydrofuran were purchased from Fisher Scientific.

Analytical thin-layer chromatography (TLC) was performed on aluminium sheets precoated with silica gel 60 (F_{254}, Merck) and visualized under ultra-violet light (short and long-wave) and using potassium permanganate ($KMnO_4$) or vanillin stains. Silica gel was purchased from Sigma Aldrich (Tech Grade, pore size 60 Å, 230-400 mesh).

^1H and ^{13}C NMR spectra were recorded in d_4-MeOD, $CDCl_3$ or D_2O on the following Bruker Avance instruments: DPX-300 300 MHz, DPX-400 400 MHz, DRX-500 500 MHz, AV III-500 HD 500 MHz, AV-600 600 MHz or AV-700 700 MHz.

High-resolution mass spectra (HRMS) of newly made compounds were obtained using electrospray ionization (ESI) on a MaXis UHR-TOF (Bruker Daltonics) or on Bruker MaXis (ESI-HR-MS).

Compounds were purified by semipreparative HPLC on a Phenomenex synergi™ Polar RP 80 Å (250 x 10.0 mm, 4µm) column. The mobile phase consisted of a gradient of water and acetonitrile (HPLC grade, containing 0.1 % trifluoroacetic acid) at a flow rate of 2.5 mL/min, with UV detection at 210, 254 and 280 nm.

1.2 Synthesis of probe 5 (butyl 6-acetamido-3-oxohexanoate)

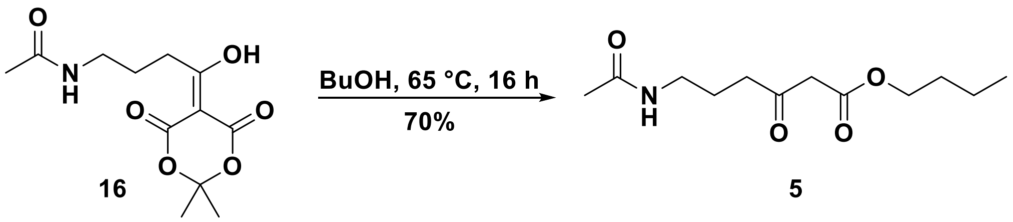

N-(4-(2,2-dimethyl-4,6-dioxo-1,3-dioxan-5-ylidene)-4-hydroxybutyl)acetamide[1a] (**16**, 250 mg, 0.92 mmol) was dissolved in dry butanol (15 mL) and the reaction was stirred at 65 °C. After 16 h

the solvent was removed under reduced pressure. The crude material was purified by column chromatography (CH_2Cl_2/MeOH 9 : 1) delivering compound **5** as a white solid (157 mg, 70%); R_f = 0.35 **^{1}H-NMR** (400 MHz, $CDCl_3$): δ_H 5.75 (br s, 1H, NH), 4.07 (m, *J* 6.6 Hz, 2H, CH_2), 3.49 (s, 2H, CH_2), 3.19 (m, *J* 6.8 Hz, 2H, CH_2), 2.55 (t, *J* 6.8 Hz, 2H, CH_2), 1.91 (s, 3H, CH_3), 1.75 (quint, *J* 6.8 Hz, 2H, CH_2), 1.55 (quint, *J* 6.6 Hz, 2H, CH_2), 1.32 (qt, *J* 7.4, 6.6 Hz, 2H, CH_2), 0.85 (t, *J* 7.4 Hz, 3H, CH_3); **HRMS**: $[M+Na]^+$ calculated: 266.1368, found: 266.1345.

1.3 Synthesis of triketide substrate 13 (*N*-(6-hydroxy-4,8-dioxo-nonyl)acetamide)

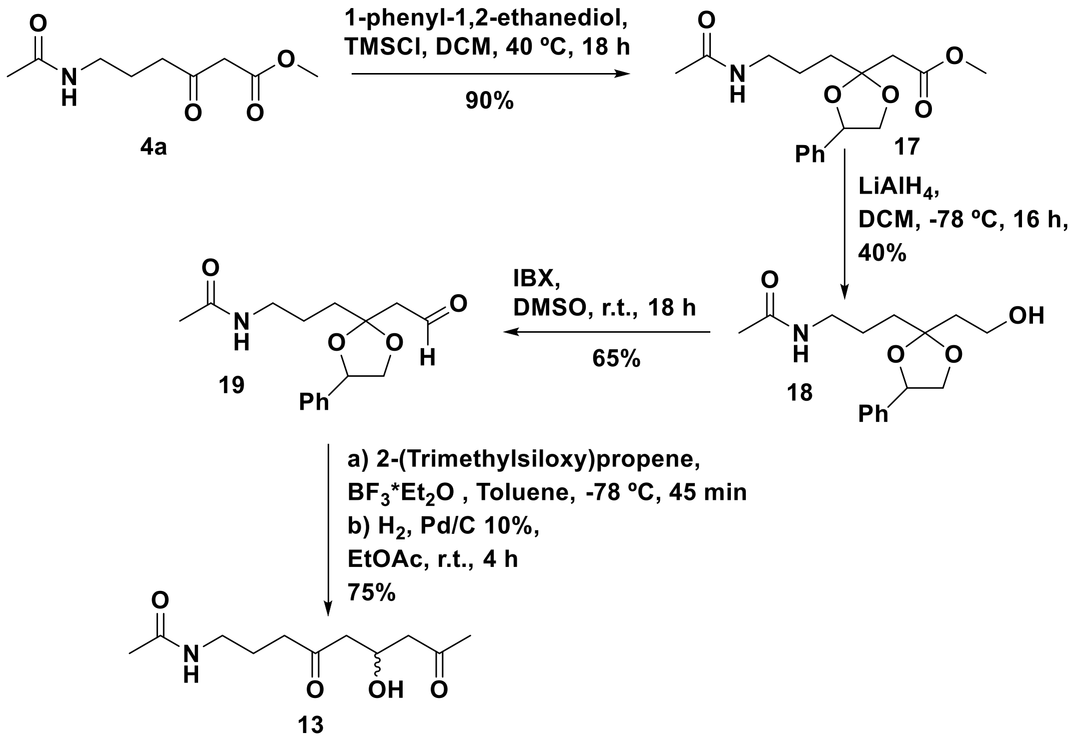

1.3.1 Methyl 2-(2-(3-acetamidopropyl)-4-phenyl-1,3-dioxolan-2-yl)acetate (17)

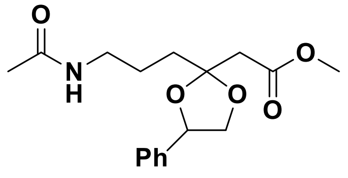

To a solution of methyl 6-acetamido-3-oxohexanoate[1a] (**4a**, 1.40 g, 7.0 mmol) in dry dichloromethane (40 mL), 1-phenyl-1,2-ethanediol (1.92 g, 13.9 mmol) and then chlorotrimethylsilane (3.51 mL, 27.9 mmol) were added dropwise under argon atmosphere. The reaction mixture was heated under reflux at 40 °C for 18 h. The solvent was removed *in vacuo* and

the resulting residue dissolved in EtOAc (40 mL) and washed with 5% $NaHCO_3$ (20 mL). The organic layer was dried over $MgSO_4$, filtered and concentrated to afford **17** (2.01 g, 90%).

^{1}H-NMR (400 MHz, $CDCl_3$) δ 7.37-7.29 (m, 10H, ArH), 5.98 (br s, 1H, NH), 5.15 and 5.05 (m, each 1H, *J* 6.2 Hz, CH), 4.37-4.31 (m, 2H, *J* 8.0, 6.5 Hz, CH_2), 3.71 (s, 3H, CH_3), 3.70 (s, 3H, CH_3), 3.70 (t, 2H, *J* 8.5 Hz, CH_2), 3.33-3.27 (m, 4H, CH_2NH), 2.80 and 2.80 (d, each 2H, *J* 15.0 Hz, CH_2), 2.04-1.92 (m, 4H, CH_2), 1.97 and 1.95 (s, each 3H, CH_3), 1.79-1.68 (m, 4H, CH_2); **^{13}C-NMR** (125 MHz, $CDCl_3$) δ 170.3 (s, COOR), 169.8 (s, CONH), 137.7 (C arom), 128.6 (CH), 128.3 (CH), 126.3 (CH), 109.9 (CO_2), 78.9 (CH), 71.9 (CH_2O), 51.8 (CH_3O), 43.1 (CH_2), 39.5 (CH_2), 35.2 (CH_2), 23.5 (CH_3), 23.1 (CH_2); **HRMS**: m/z $[M + Na]^+$ found: 344.1470, calculated: 344.1468.

1.3.2 *N*-(3-(2-(2-hydroxyethyl)-4-phenyl-1,3-dioxolan-2-yl)propyl)acetamide (18)

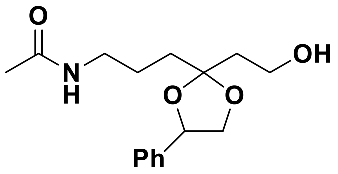

Compound **17** (200 mg, 0.6 mmol) was dissolved in dichloromethane (5 mL) under argon and cooled to -78 °C. To this lithium aluminium hydride (1 M solution in THF, 3.12 mL, 3.1 mmol) was added. The reaction mixture was kept at -78 °C for 1 hour before being allowed to warm at room temperature over 15 h. Water (3 mL) was slowly added at 0 °C followed by saturated aqueous NH_4Cl (9 mL) and water (3 mL). The resulting slurry was filtered and extracted with ethyl acetate. After concentrating this, the crude residue was purified by silica gel chromatography (CH_2Cl_2/MeOH 9 : 1) to give **18** (73 mg, 40%); R_f = 0.5.

^{1}H-NMR (400 MHz, $CDCl_3$): δ 7.26-7.19 (m, 5H, ArH), 5.51 (br s, 1H, NH), 4.98 (m, 1H, CH), 4.22 (m, 1H, CH), 3.71 (m, 2H, CH_2), 3.60 (m, 1H, CH), 3.17 (dt, *J* 6.6, 6.1 Hz, 2H, CH_2), 2.54 (br, 1H, OH), 1.92 (m, 2H, CH_2), 1.83 (s, 3H, $COCH_3$), 1.71 (m, 2H, CH_2), 1.57 (m, 2H, CH_2); **^{13}C-NMR** (400 MHz, $CDCl_3$): δ 170.0 (RCOR), 137.9 (C arom), 128.8 (CH), 126.4 (CH), 126.2 (CH), 112.6 (R_2CO_2), 78.4 ($OCHR_2$), 71.6 (H_2COR), 58.8 (CH_2OH), 39.6 (CH_2), 38.6 (CH_2), 34.6 (CH_2), 24.2 (CH_2), 23.3 (CH_3); **HRMS**: m/z $[M + Na]^+$, found: 316.1518, calculated: 316.1519.

1.3.3 *N*-[3-[2-(2-oxoethyl)-4-phenyl-1,3-dioxolan-2-yl]propyl]acetamide (19)

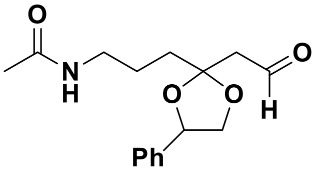

Compound **18** (73 mg, 0.3 mmol) and 2-iodoxybenzoic acid (IBX, 126 mg, 0.4 mmol) were dissolved in dry DMSO (1 mL). The reaction mixture was stirred for 18 hours before adding water (10 mL) and ethyl acetate (10 mL). The layers were separated and the organic phase was filtered through Celite, dried and purified by silica gel chromatography (CH_2Cl_2/THF 1 : 1) to give **19** as a yellow oil (47 mg, 65%); ; R_f = 0.55.

^{1}H-NMR (400 MHz, $CDCl_3$): δ 9.72 (t, *J* 2.8 Hz, 1H, RCHO), 7.28-7.24 (m, 5H, ArH), 5.55 (br s, 1H, NH), 5.01 (m, 1H, CH), 4.29 (m, 1H, CH), 3.65 (m, 1H, CH), 3.21 (dt, *J* 6.6, 6.1 Hz, 2H, CH_2), 2.72 (d, *J* 2.9 Hz, 2H, CH_2), 1.91 (s, 3H, CH_3), 1.80 (m, 2H, CH_2), 1.60 (m, 2H, CH_2); **HRMS**: m/z $[M + Na]^+$, found: 314.1370, calculated: 314.1363.

1.3.4 *N*-(6-hydroxy-4,8-dioxo-nonyl)acetamide (13)

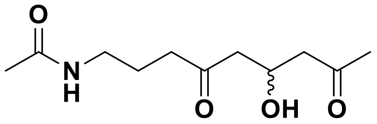

To compound **19** (47 mg, 0.16 mmol) in toluene (2.5 mL), 2-(trimethylsiloxy)propene (20 µl, 0.10 mmol, 85% solution) and boron trifluoride diethyl etherate (25 µl, 0.20 mmol) were added. The reaction mixture was stirred for 45 minutes before adding saturated $NaHCO_3$ solution (2.5 ml). The organic phase was extracted with dichloromethane (5 mL), dried over Na_2SO_4 and evaporated. The crude material was purified by silica gel chromatography (CH_2Cl_2/THF from 4 : 1 to 1 : 1). The obtained yellow oil (39 mg, 0.11 mmol) was dissolved in EtOAc (5 mL) under H_2 atmosphere. Pd/C 10% (90 mg) was added and the reaction was stirred at r.t. for 4 h. The reaction was filtered through Celite and the solvent was evaporated under reduced pressure. The crude product was purified by semipreparative HPLC (elution gradient starting from 2% acetonitrile and linearly increasing to 10% over 8 minutes, then isocratically at 10% for 20 minutes) to yield **13** (26 mg, 80%); Rt: 17.6 min.

^{1}H-NMR (400 MHz, $CDCl_3$): δ 5.66 (br s, 1H, NH), 4.48 (m, 1H, CH), 3.49 (d, *J* 3.3 Hz, 1H, OH), 3.23 (m, 2H, CH_2), 2.59 (m, 2H, CH_2), 2.53 (m, 2H, CH_2), 2.48 (t, *J* 6.7 Hz, 2H, CH_2) 2.20 (s, 3H, CH_3), 1.97 (s, 3H, $COCH_3$), 1.80 (quint, *J* 6.7 Hz, 2H, CH_2); **^{13}C-NMR** (400 MHz, $CDCl_3$): δ 210.1 (CO), 208.5 (CO), 170.5

(NHCO), 62.2 (CHOH), 49.3 (CH_2), 48.6 (CH_2), 40.9 (CH_2), 38.9 (CH_2), 30.8 (CH_3), 23.3 (CH_3), 23.2 (CH_2); **HRMS**: m/z $[M + Na]^+$, found: 252.1206, calculated: 252.1206.

To verify that **13** was a racemic mixture it was subjected to chiral chromatography. Its analysis was performed on a ChiralCel OD column (250 x 4.6 mm) at a flow rate of 0.8 mL/min (eluent: mixture (v/v) of 93% hexane and 7% ethanol) for 15 minutes. In these conditions compound **13** separated into two peaks with retention times of 3.71 and 4.36 minutes respectively (Figure 1S).

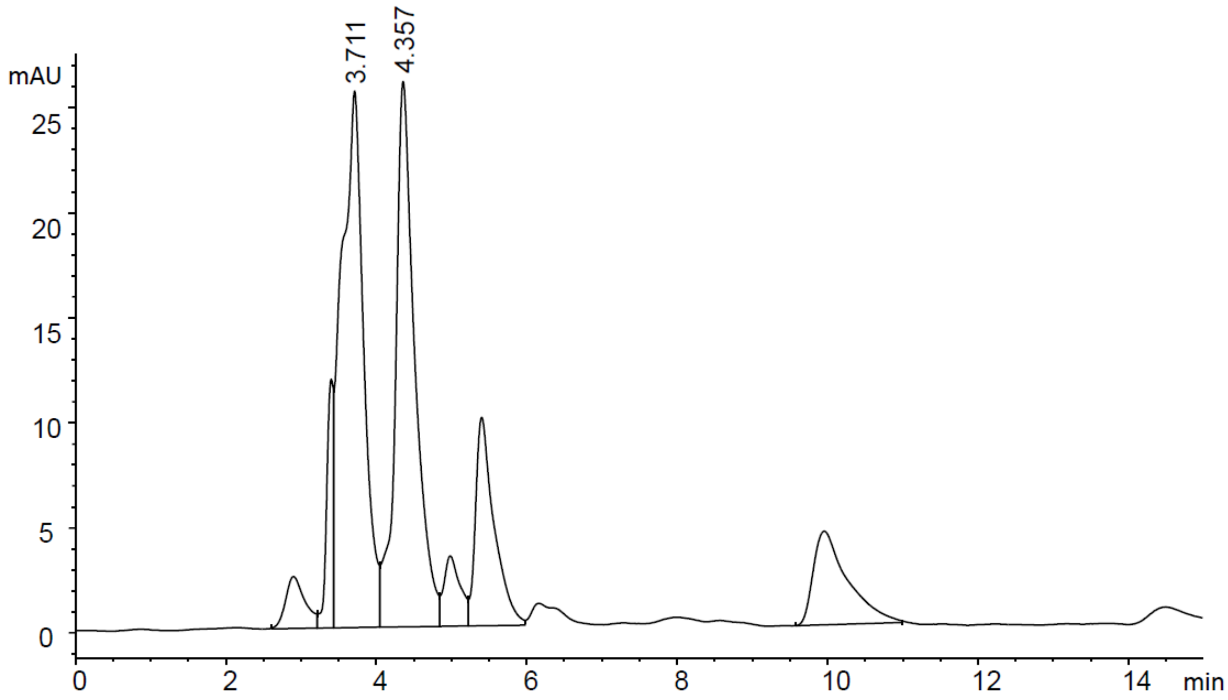

Figure 1S: Chiral analysis of compound **13** on a ChiraCel OD column.

1.4 Synthesis of 6-MSA *N*-acetylcysteamine thioester (14)

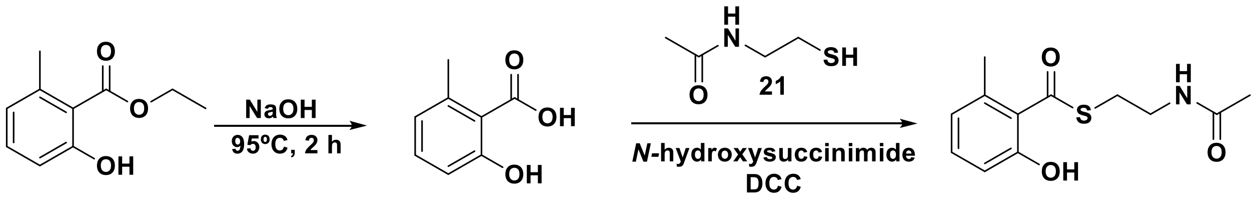

20 1 14

6-Methylsalicylic acid (6-MSA, **1**) was prepared by a modified procedure from Silverman *et al.*[2] Ethyl 6-methylsalicylate (**20**, 100 mg, 0.60 mmol) was dissolved in NaOH (2.35 M, 5 mL) and heated under reflux for 2 hours. The solution was cooled before acidifying with HCl (1 M). The white precipitate was filtered and dried to give **1** (73.0 mg, 99%). The spectroscopic data for **1** were in agreement with those reported in literature.[3]

^{1}H-NMR (300 MHz, MeOD): δ 7.16 (t, *J* 8.0 Hz, 1H, ArH), 6.65 (apt t, *J* 5.5 Hz 2H, ArH), 2.44 (s, 3H, CH_3), 2.07 (s, 1H, OH); **HRMS:** m/z $[M + H]^+$, found: 153.0549, calculated: 153.0552.

6-MSA *N*-acetylcysteamine thioester (**14**) was then synthesized following a literature procedure utilizing *N*-acetylcysteamine (**21**), *N*-hydroxysuccinimide and *N*,*N*'-dicyclohexylcarbodiimide. The spectroscopic data of **14** were in accordance with those reported in literature.[4]

^{1}H-NMR (300 MHz, MeOD): δ 7.13 (t, *J* 7.9 Hz, 1H, ArH), 6.71 (d, *J* 5.0 Hz, 1H, ArH), 6.69 (d, *J* 5.8 Hz, 1H, ArH), 3.46 (t, *J* 6.7 Hz, 2H, C*H*$_2$NH), 3.20 (t, *J* 6.7 Hz, 2H, C*H*$_2$S), 2.26 (s, 3H, CH_3), 1.96 (s, 3H, $COCH_3$).

1.5 Synthesis of 6-MSA *N*-decanoylcysteamine thioester (15)

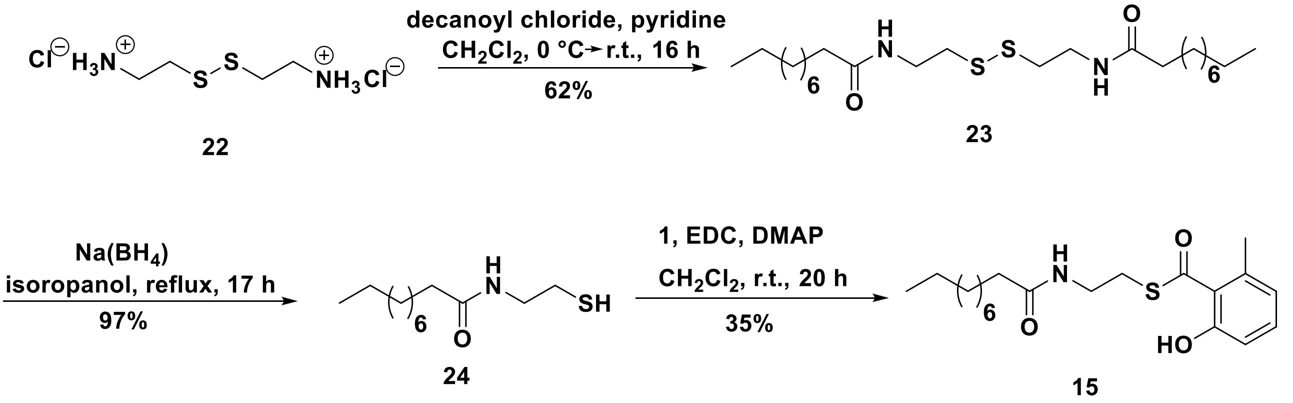

1.5.1 Synthesis of *N*, *N'*-didecanoylcystamine (23)

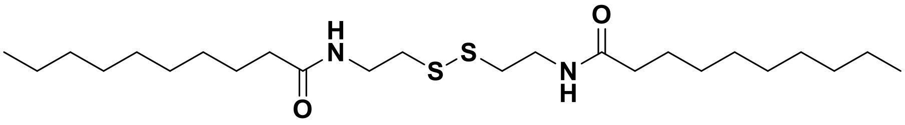

N, *N'*-didecanoylcystamine (**23**) was synthesized adapting a procedure from Withers *et al.*[5]

Cystamine hydrochloride (**22**, 0.90 g, 4.00 mmol) was dissolved in dry dichloromethane (20 mL) and dry pyridine (1.3 mL, 16.1 mmol). The mixture was cooled to 0 °C before decanoyl chloride (1.83 mL, 8.80 mmol) was slowly added. The reaction mixture was stirred overnight at room temperature and afterwards methanol (2 mL) was added at 0 °C. After continuous stirring for 30 min, the mixture was diluted with dichloromethane, washed with aqueous HCl (1 M), dried over $MgSO_4$ and filtered. The dichloromethane was removed under reduced pressure to obtain the crude product as a solid. Recrystallization from EtOAc afforded *N*, *N'*-didecanoylcystamine (**23**) as a white solid (1.13 g, 62%).

^{1}H-NMR (500 MHz, $CDCl_3$): δ_H 6.29 (t, *J* 5.0 Hz, 2H, NH), 3.58 (app q, *J* 6.4 Hz, 4H, C*H*$_2$NH), 2.84 (t, *J* 6.4 Hz, 4H, CH_2S), 2.22 (t, *J* 7.5 Hz, 4H, CH_2CO), 1.57 - 1.70 (m, 4H, C*H*$_2$$CH_2CO$), 1.19 - 1.38 (m, 24H, CH_2), 0.88 (t, *J* 6.9 Hz, 6H, CH_3); **^{13}C-NMR** (126 MHz, $CDCl_3$): δ_C 173.7 (CONH), 38.3 (CH_2NH), 37.8 (CH_2S), 36.6 (*C*H$_2$CO), 31.7 (CH_2), 29.5 (CH_2), 29.4 (CH_2), 29.3 (CH_2), 29.3 (CH_2), 25.7 (CH_2), 22.7 (CH_2), 14.10 (CH_3); **HRMS**: *m/z* $[M + Na]^+$, found: 483.3054, calculated: 483.3049.
^{1}H-NMR data are in agreement with those previously reported.[6]

1.5.2 Synthesis of *N*-decanoylcysteamine (24)

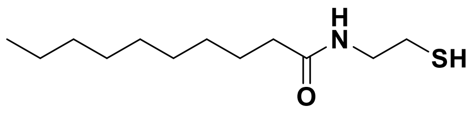

N-decanoylcysteamine (**24**) was synthesized adapting literature procedure.[7]
N, *N'*-didecanoylcystamine (**23**, 242 mg, 0.53 mmol) was dissolved in dry isopropanol (5 mL). The solution was degassed by bubbling argon for 45 min. Sodium borohydride (100 mg, 2.63 mmol) was then added and the reaction mixture was heated to reflux for 16 h. The reaction was monitored by TLC and further sodium borohydride (10 mg, 0.26 mmol) was added. The reaction mixture was allowed to cool to room temperature and methanol (10 mL) was added. The reaction was quenched with acetic acid (2.8 mL) at 0 °C and concentrated *in vacuo*. The crude product was purified by column chromatography (cyclohexane/ethyl acetate from 3:1 to 2:1) to obtain **24** as a white solid (249 mg, 97%); R_f = 0.13 (cyclohexane/ethyl acetate 3:1).
^{1}H-NMR (500 MHz, $CDCl_3$): δ_H 3.44 (q, *J* 6.1 Hz, 2H, C*H*$_2$NH), 2.68 (dt, *J* 8.4, 6.4 Hz, 2H, C*H*$_2$SH), 2.16 - 2.23 (m, 2H, CH_2CO), 1.57 - 1.69 (m, 2H, C*H*$_2$$CH_2CO$), 1.20 - 1.37 (m, 12H, CH_2), 0.88 (t, *J* 6.9 Hz, 3H, CH_3); **^{13}C-NMR** (126 MHz, $CDCl_3$): δ_C 173.2 (CONH), 42.2 (CH_2NH), 36.8 (*C*H$_2$CO), 29.4 (CH_2), 29.3 (CH_2), 29.3 (CH_2), 29.2 (CH_2), 25.7 (CH_2SH), 24.8 (CH_2), 22.6 (CH_2), 14.1 (CH_3); **HRMS**: *m/z* $[M+Na]^+$, found: 254.1549, calculated: 254.1549.

1.5.3 Synthesis of 6-MSA *N*-decanoylcysteamine thioester (15)

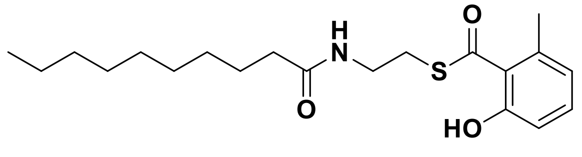

The compound was synthesized adapting a method from Hertweg *et al*.[8]

6-MSA (**1**, 47 mg, 0.31 mmol) was suspended in dichloromethane (5 mL) and 4-dimethylaminopyridine (37.9 mg, 0.31 mmol), *N*-decanoylcysteamine (**24**) (74 mg, 0.32 mmol) and 1-ethyl-3-(3-dimethylaminopropyl)carbodiimide (EDC, 48 mg, 0.31 mmol) were added. The reaction mixture was stirred at room temperature for 16 h. The reaction mixture was diluted with 10 mL of dichloromethane and extracted with aqueous HCL (10 mL, 1 M). The organic layer was dried over magnesium sulphate, filtered, and concentrated *in vacuo*. The crude mixture was purified by HPLC (solvent A: water (1% TFA), solvent B: acetonitrile (1% TFA); gradient: 10% A, 0 min to 100% B, 30 min). The pure product **15** was obtained as a white solid (40 mg, 35 %); Rt: 26.6 min.

^{1}H-NMR (500 MHz, $CDCl_3$): δ_H 9.87 (br s, 1H, OH), 7.24 (d, *J* 7.9 Hz, 1H, ArH), 6.84 (d, *J* 8.2 Hz, 1H, ArH), 6.75 (d, *J* 7.5 Hz, 1H, ArH), 5.86 (s, 1H, NH), 3.60 (q, *J* 6.1 Hz, 2H, CH_2NH), 3.26 (t, *J* 6.3 Hz, 2H, CH_2S), 2.61 (s, 3H, CH_3C), 2.18 (t, *J* 7.6 Hz, 2H, CH_2CO), 1.55 - 1.71 (m, 4H, CH_2), 1.19 - 1.34 (m, 10H, CH_2), 0.88 (t, *J* 7.0 Hz, 3H, CH_3);**^{13}C-NMR** (126 MHz, $CDCl_3$): δ_C 197.8 (COS), 173.8 (CONH), 158.2 (COH), 138.1 (*C*CH_3), 133.5 (C), 123.2 (C), 123.0 (*C*COS), 115.9 (C), 39.0 (CH_2NH), 36.8 (CH_2S), 31.8 (CH_2), 29.9 (CH_2), 29.4 (CH_2), 29.3 (CH_2), 29.3 (CH_2), 25.7 (CH_2), 23.1 (CH_2), 22.6 (CH_2), 14.1 (CH_3); **HRMS**: *m/z* $[M + Na]^+$, found: 388.1921, calculated: 388.1917.

2 *In vivo* experiments

2.1 Microbiology methods

All media and glassware were sterilized prior to use by autoclave (Astell). Liquid cultures were grown with shaking in Innova 44 incubator/shaker (New Brunswick scientific).

P. patulum was grown from spores on Czapek media agar (30 g sucrose, 0.5 g magnesium glycerophosphate, 2 g $NaNO_3$, 0.5 g KCl, 0.35 g K_2SO_4, 0.01 g $FeSO_4{\cdot}7H_2O$, 12 g agar in 1 L of distilled water adjusted to pH 6.8) plates (15 mL) at 25°C for 1 week. Spores were washed and filtered with 1 mL mycelial solution (40 µL IPEGAL® CA-630 detergent in 100 mL 1 % (w/v) NaCl), 200 µL of which was used to inoculate 25 mL of *P. patulum* growth medium (5 g yeast extract, 2.5 g KH_2PO_4, 2.5 g K_2HPO_4, 40 g glucose, 0.5 g $MgSO_4{\cdot}7H_2O$, 0.1 g KCl in 1 L of distilled water).

A single colony of *E. coli* BAP1 pKOS007-109[9] was used to inoculate 10 mL of LB medium and grown overnight at 37°C and 100 µL of the overnight culture was used to inoculate 10 mL LB or ATCC medium 765 with 10 % glycerol (4.49 g KH_2PO_4, 11.67 g K_2HPO_4, 1.98 g $(NH_4)_2SO_4$, 0.5 mg $FeSO_4{\cdot}7H_2O$, 0.25 g $MgSO_4$, 100 mL glycerol, 11 g casamino acids in 1 L of distilled water) and grown at 37 °C.

S. antibioticus was grown in SFM (20 g/L soya flour, 20 g/L mannitol) with 0.2 % $CaCO_3$. 100 µL of a spore stock solution (40% glycerol) were used to inoculate 10 mL of SFM medium with 0.2% $CaCO_3$ and grown for 3 days at 30 °C. 100 µL of the precultured cells were used to inoculate 10 mL of the same medium and grown at 30 °C.

2.2 Growth of *Penicillium patulum*, *Escherichia coli* BAP1 pKOS007-109, *Streptomyces antibioticus* DSM40725 strains and mass spectrometry analysis

Spores of *P. patulum* were used to inoculate 10 mL of growth medium containing chain termination probes at 5 or 10 mM final concentration (previously dissolved in MeOH, 20 µL). Cultures were then grown for 2 days at 30°C. Control liquid cultures in absence of chain termination probes were also prepared (in duplicate/triplicate copy). Each flask was extracted twice with 20 mL of EtOAc which was removed *in vacuo* and the residue was then dissolved in HPLC grade methanol (0.5 mL).

E. coli BAP1 pKOS007-109[9] was grown in LB or ATCC medium 765 until the A_{600} reached 0.6. After adding 0.5 mM isopropyl ß-D-1-thiogalactopyranoside and 2 to 10 mM of the chain termination

probe, the cultures were incubated at 30 °C for 24 h. Each culture was then extracted twice with ethyl acetate, the extracts combined and the solvent removed *in vacuo*. The residue was dissolved in 0.5 mL of HPLC grade methanol for mass spectrometry analysis.

S. antibioticus strain was grown in SFM with 0.2% $CaCO_3$ medium (10 mL) for 3 days at 30 °C in 50 mL Erlenmeyer flasks with spring. Seed cultures (100 µL) were used to inoculate SFM with 0.2% $CaCO_3$ medium liquid cultures (10 mL, in duplicate/triplicate copy, in 50 mL Erlenmeyer flasks with spring). They were incubated at 30 °C for 5 days. After the first day of incubation, the different probes were added portion wise as follows[1b]: addition of 8.3 µmol dissolved in 100 µl of MeOH on day 2 and day 3; addition of 4.1 µmol dissolved in 50 µl of MeOH on day 4 and 5. Control liquid cultures in absence of the probes were also prepared (in duplicate/triplicate copy). After 5 days of fermentation, the liquid cultures were extracted with ethyl acetate (20 mL x 2). The extracts were concentrated and the residues were redissolved in HPLC-grade methanol (1 mL) for mass spectrometry analysis.

LC-MS analyses of ethyl acetate extracts were performed on a MaXis Impact UHR-TOF and/or on a Thermo Orbitrap Fusion (Q-OT-qIT, Thermo) instrument unless otherwise stated.

UPLC-HR-ESI-MS analyses of ethyl acetate extracts on a MaXis Impact UHR-TOF (Bruker Daltonics): samples (5 µl) were injected onto an Acquity UPLC HSS T3 (150 mm x 1.0 mm, 1.8 µm) or Agilent Eclipse C18 (1.8 um, 100 mm x 2.1 mm). The mobile phase consisted of a gradient of water and acetonitrile (HPLC grade, each with 0.1% trifluoroacetic acid). The following solvent (A =1% TFA in H_2O, B =1% TFA in MeCN) gradient was applied: 10% B 0-2.7 min; 10-100% B 2.7-42.7 min; 100% B 42.7-52.7 min; 100-10% B 52.7-55.7 min; 10% B 55.7-67.7 min, using an Acquity UPLC HSS T3 column at a flow rate of 0.05 mL/min. Spectra were recorded in positive ionisation mode, scanning from *m/z* 100 to 3000, with the resolution set at 45K. Selected ion search within 5 ppm was performed.

Further *high resolution analyses* for ethyl acetate extracts obtained from *N*-pentynoyl and *N*-decanoyl probes were performed on a *Thermo Orbitrap Fusion* (Q-OT-qIT, Thermo) instrument. Reversed phase chromatography was used to separate the mixtures prior to MS analysis. Two columns were utilized: an Acclaim PepMap µ-precolumn cartridge 300 µm i.d. x 5 mm 5 µm 100 Å and an Acclaim PepMap RSLC 75 µm x 15 cm 2 µm 100 Å (Thermo Scientific). The columns were installed on an Ultimate 3000 RSLCnano system (Dionex). Mobile phase buffer A was composed of 0.1% aqueous formic acid and mobile phase B was composed of 100% acetonitrile containing 0.1% formic acid. Samples were loaded onto the µ-precolumn equilibrated in 2% aqueous acetonitrile

containing 0.1% trifluoroacetic acid for 8 min at 10 µL min^{-1} after which compounds were eluted onto the analytical column following a 45 min or a 75 min gradient for which the mobile phase B concentration was increased from 50% B to 99.5% over 15 min, then maintained at 99.5% B for 5/ 35 minutes, then decreased to 50% over 16 min, followed by a 9 min wash at 50% B. Eluting cations were converted to gas-phase ions by electrospray ionization and analyzed. Survey scans of precursors from 150 to 1500 *m/z* were performed at 60K resolution (at 200 *m/z*) with a 5×10^5 ion count target. Tandem MS was performed by isolation at 0.7 Th with the quadrupole, HCD fragmentation with normalized collision energy of 30, and rapid scan MS analysis in the ion trap. The MS^2 ion count target was set to 10^4 and the maximum injection time was 35 ms. A filter targeted inclusion mass list was used to select the precursor ions. The dynamic exclusion duration was set to 45 s with a 10 ppm tolerance around the selected precursor and its isotopes. Monoisotopic precursor selection was turned on. The instrument was run in top speed mode with 5 s cycles, meaning the instrument would continuously perform MS^2 events until the list of nonexcluded precursors diminishes to zero or 5 s, whichever is shorter. Fusion runs were performed with Survey scans of precursors from 150 to 1500 *m/z* 60K resolution (at 200 *m/z*) with a 1×10^6 ion count target. Tandem MS was performed by isolation at 1.8 Th with the ion-trap, CAD fragmentation with normalized collision energy of 32, and 15K resolution scan MS analysis in the Orbitrap. The data dependent top 20 precursors were selected for MS^2. MS^2 ion count target was set to 4×10^6 and the max injection time was 50 ms. The dynamic exclusion duration was set to 40 s with a 10 ppm tolerance around the selected precursor and its isotopes.

2.3 Summary of 6-MSA intermediates captured from *P. patulum*, *E. coli* BAP1 pKOS007-109 and *S. antibioticus* DSM40725

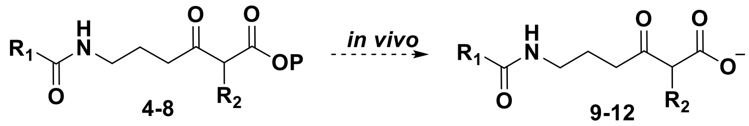

	R_1	R_2	P
**4a**	CH_3	H	Me
**4b**	CD_3	H	Me
**5**	CH_3	H	Bu
**6**	$(CH_2)_2C{\equiv}CH$	H	Me
**7**	$(CH_2)_2C{\equiv}CH$	F	Me
**8**	$(CH_2)_8CH_3$	H	Me

	R_1	R_2
**9a**	CH_3	H
**9b**	CD_3	H
**9a**	CH_3	H
**10**	$(CH_2)_2C{\equiv}CH$	H
**11**	$(CH_2)_2C{\equiv}CH$	F
**12**	$(CH_2)_8CH_3$	H

Figure 2S: Overview of probes utilized for *in vivo* intermediate capture.

Table 1S: Overview of intermediates captured from *P. patulum via* probes **4a-b**, **5**, **6** and **8**.

intermediate	putative structure	probe **4a**[a, b] $R_1=CH_3$	probe **4b**[a, b] $R_1=CD_3$	probe **5**[a] $R_1=CH_3$	probe **6** [a] $R_1=(CH_2)_2C{\equiv}CH$	probe **8** [c] $R_1=(CH_2)_8CH_3$
Diketide	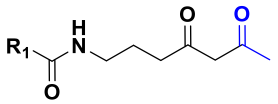	✓	✓	✓	✓	✓
Triketide	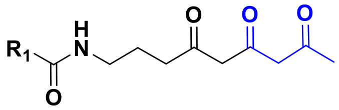	✓	✓			✓
Reduced triketide	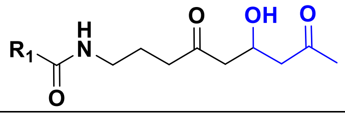	✓	✓	✓	✓	✓
Hydroxy tetraketide	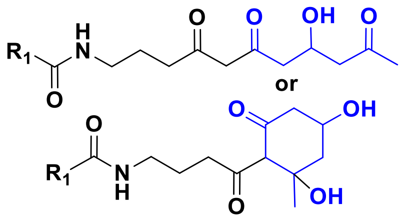	✓	✓	✓	✓	✓
Dehydrated tetraketide	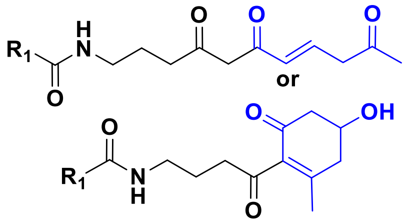			✓		✓ traces

continuing next page

continuing from previous page

intermediate	putative structure	probe **4a**[a, b] $R_1=CH_3$	probe **4b**[a, b] $R_1=CD_3$	probe **5**[a] $R_1=CH_3$	probe **6** [a] $R_1=(CH_2)_2C\equiv CH$	probe **8** [c] $R_1=(CH_2)_8CH_3$
Aromatized tetraketide	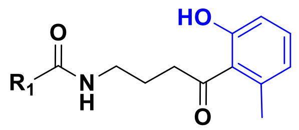					✓
Hydroxy pentaketide	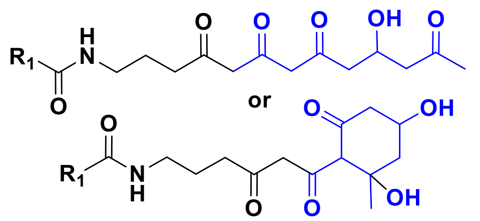					✓
Dehydrated pentaketide	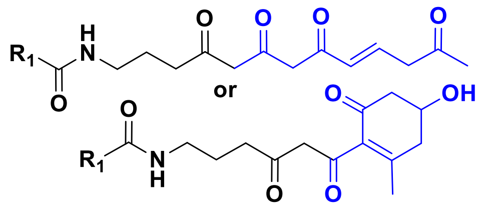	✓ traces	✓ traces	✓	✓	✓ traces
Aromatized pentaketide	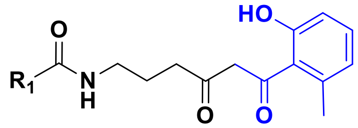			✓		✓

[a] analyzed on a Maxis Bruker instrument; [b] analyzed on an Orbitrap Classic instrument; [c] analyzed by an Orbitrap Fusion instrument.

Table 2S: Overview of intermediates captured from *E. coli* BAP1 pKOS007-109 *via* probes **4a-b**, **6** and **8**.

intermediate	putative structure	probe **4a**[a] $R_1=CH_3$	probe **4b**[a] $R_1=CD_3$	probe **6** [a] $R_1=(CH_2)_2C\equiv CH$	probe **8** [c] $R_1=(CH_2)_8CH_3$
Diketide	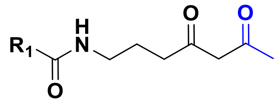	✓	✓	✓	✓
Triketide	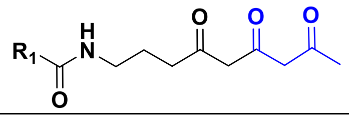	✓	✓	✓	✓
Reduced triketide	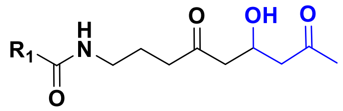				✓
Hydroxy tetraketide	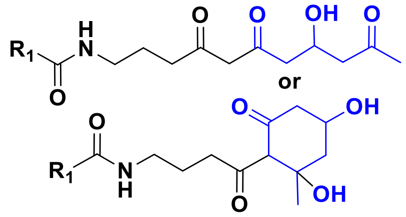			✓	✓

continuing next page

continuing from previous page

intermediate	putative structure	probe **4a**[a] $R_1=CH_3$	probe **4b**[a] $R_1=CD_3$	probe **6** [a] $R_1=(CH_2)_2C\equiv CH$	probe **8** [c] $R_1=(CH_2)_8CH_3$
Dehydrated tetraketide	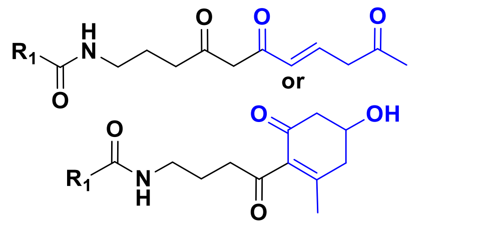			✓	✓
Aromatized tetraketide	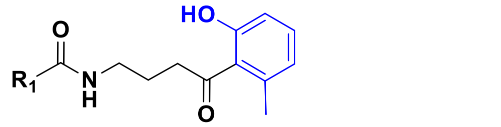				✓
Hydroxy pentaketide	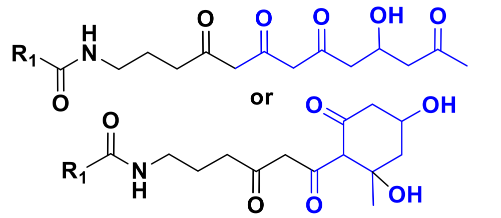	✓	✓		✓ traces
Dehydrated pentaketide	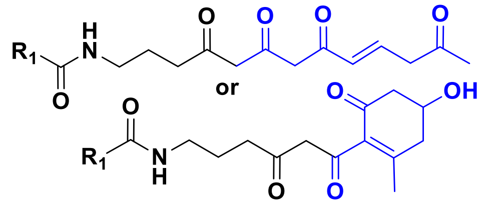			✓	✓ traces
Aromatized pentaketide	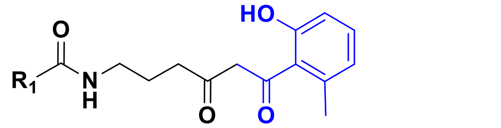			✓	✓

[a] analyzed on a Maxis Bruker instrument; [c] analyzed by an Orbitrap Fusion instrument

Table 3S: Overview of intermediates captured from *S. antibioticus* DSM40725 *via* probes **4a-b**, **6**, **7** and **8**.

intermediate	putative structure	probe **4a**[a] $R_1= CH_3$ $R_2=H$	probe **4b**[a] $R_1= CD_3$ $R_2=H$	probe **6**[c, d] $R_1=(CH_2)_2C\equiv CH$ $R_2=H$	probe **7**[c, d] $R_1=(CH_2)_2C\equiv CH$ $R_2=F$	probe **8**[c, d] $R_1=(CH_2)_8CH_3$ $R_2=H$
Diketide	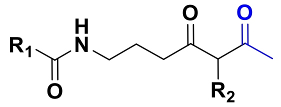	✓	✓	✓	✓	✓
Triketide	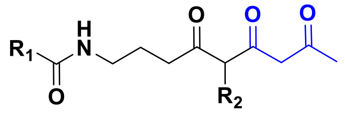	✓	✓	✓	✓	✓

continuing next page

from previous page

intermediate	putative structure	probe **4a**[a] R_1= CH_3 R_2=H	probe **4b**[a] R_1= CD_3 R_2=H	probe **6**[c, d] R_1= $(CH_2)_2C{\equiv}CH$ R_2=H	probe **7**[c, d] R_1= $(CH_2)_2C{\equiv}CH$ R_2=F	probe **8**[c, d] R_1=$(CH_2)_8CH_3$ R_2=H
Reduced triketide	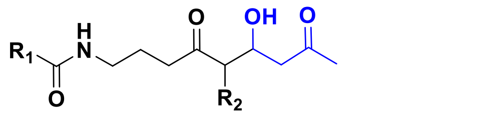				✓	✓
Hydroxy tetraketide	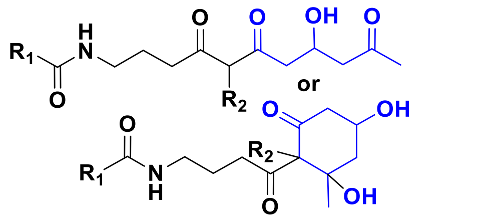			✓	✓	✓
Dehydrated tetraketide	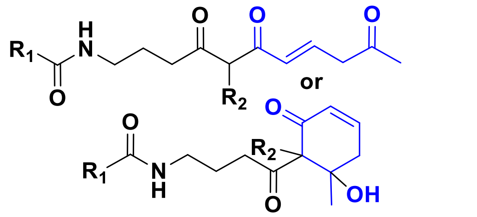			✓		✓
Aromatized tetraketide	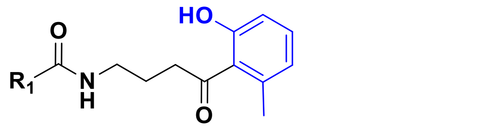					
Hydroxy pentaketide	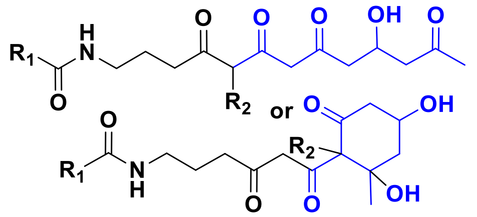			✓ traces	✓	✓
Dehydrated pentaketide	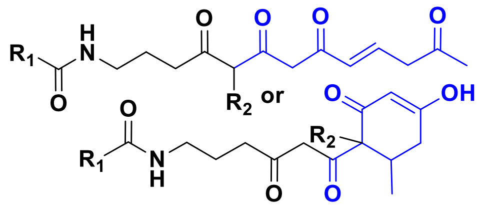			✓ traces	✓	
Aromatized pentaketide	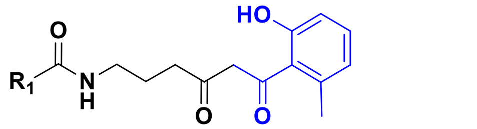			✓ traces		✓

[a] analyzed on a Maxis Bruker instrument; [c] analyzed by an Orbitrap Fusion instrument; [d] putative intermediates derived from the chlorothricin modular PKS were also identified (herein not shown).

2.4 Capture of 6-MSAS intermediates from *P. patulum* *via* chain termination probes

2.4.1 Intermediate capture by probes 4a-b [1a-c]

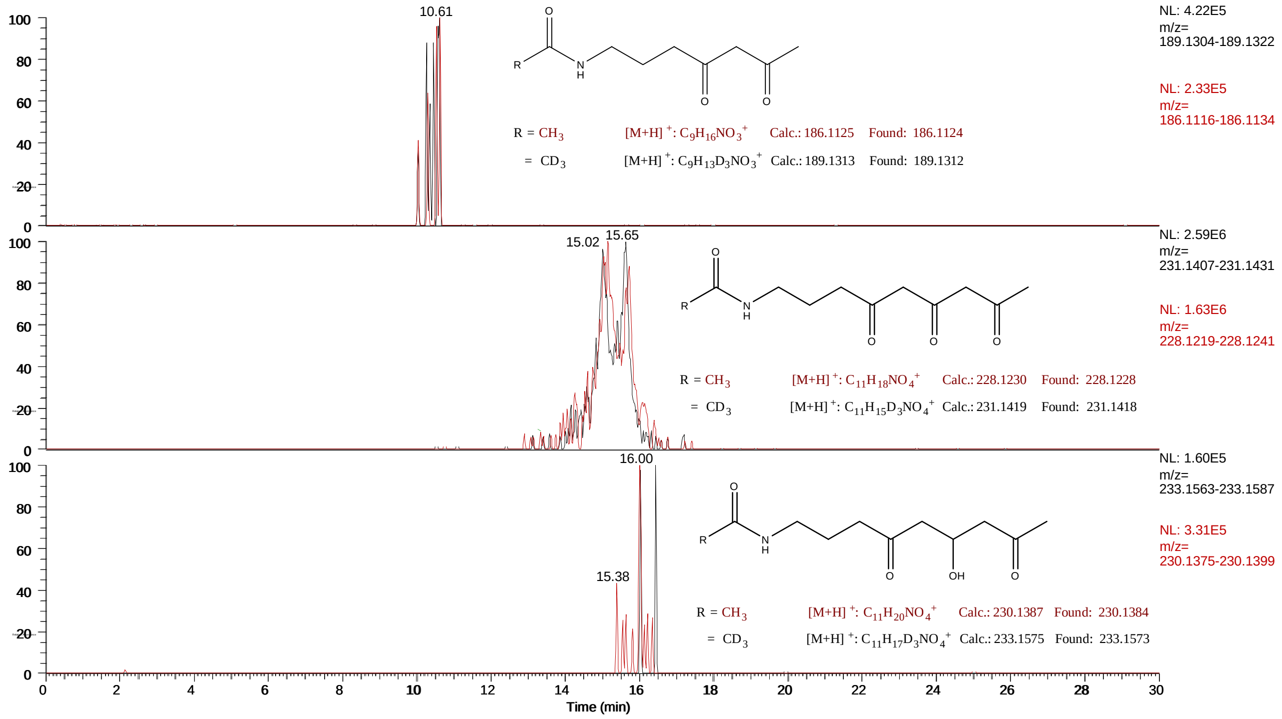

Figure 3S: LC-HRMS analyses of the organic extracts of *P. patulum* grown in the presence of probe **4a** (red) and **4b** (black; final concentration 10 mM): $[M+H]^+$ extracted ion traces and accurate mass for putative diketide and triketide species are given. The analyses were performed on an Orbitrap Classic instrument as previously reported.[1a, c] Masses corresponding to these species were not found in control samples (data not shown). Mass search for dehydrated triketides with retention times different from those of hydroxylated triketides were not found. Samples were also analyzed on a MaXis Impact instrument and hydroxy tetraketides were also identified (Figure 1B in main text). Dehydrated pentaketides were also detected in minor amounts.

2.4.2 Intermediate capture by probe 5 (butyl 6-acetamido-3-oxohexanoate)

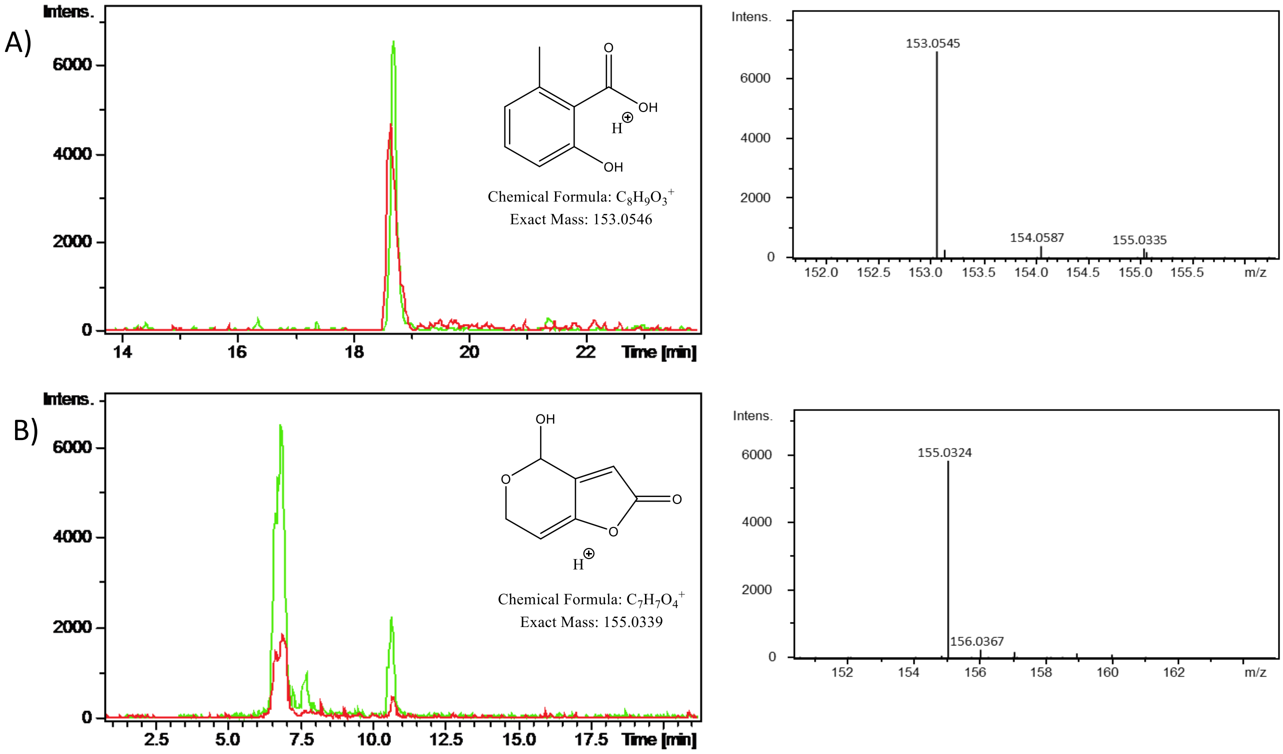

Figure 4S:LC-HRMS analyses of the organic extracts of *P. patulum* grown in the absence (green) and in the presence (red) of probe **5** (final concentration 10 mM): **A)** $[M+H]^+$ extracted ion trace (left) and high resolution mass (right) for 6-MSA (R_t = 18.7 min); **B)** $[M+H]^+$ extracted ion trace (left) and high resolution mass (right) for patulin (R_t = 7.0 min). The analyses were performed on a Bruker MaXis Impact instrument.

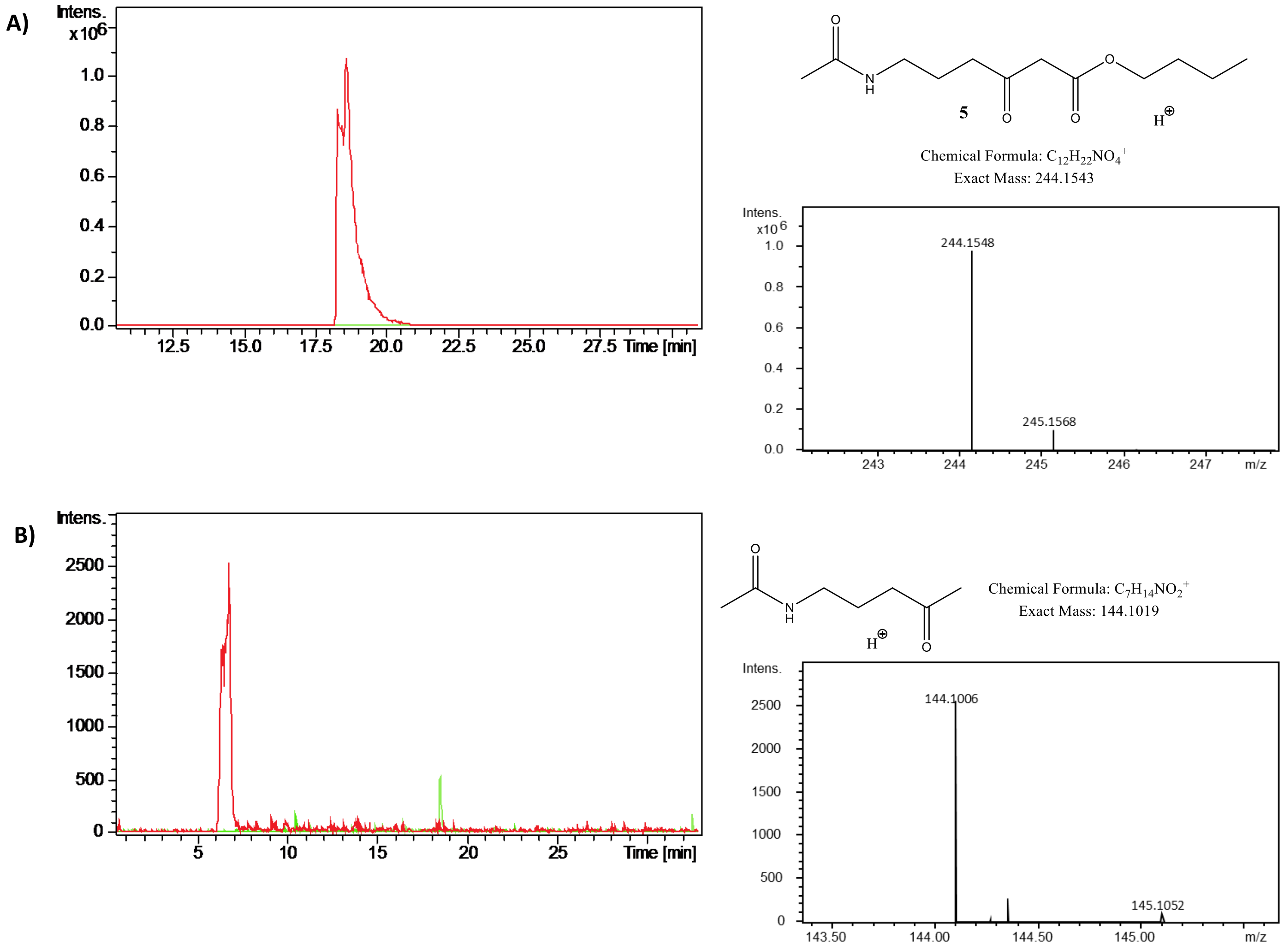

Figure 5S: LC-HRMS analysis of the organic extracts of *P. patulum* grown in the absence (green) and in the presence (red) of probe **5** (final concentration 10 mM): **A)** $[M+H]^+$ extracted ion trace (left) and high resolution mass (right) for butyl 6-acetamido-3-oxohexanoate (**5**, R_t = 18.6 min); **B)** $[M+H]^+$ extracted ion trace (left) and high resolution mass (right) for *N*-(4-oxopentyl)acetamide (R_t = 6.9 min), resulting from *in vivo* hydrolysis of **5** and decarboxylation (MaXis Impact analyses).

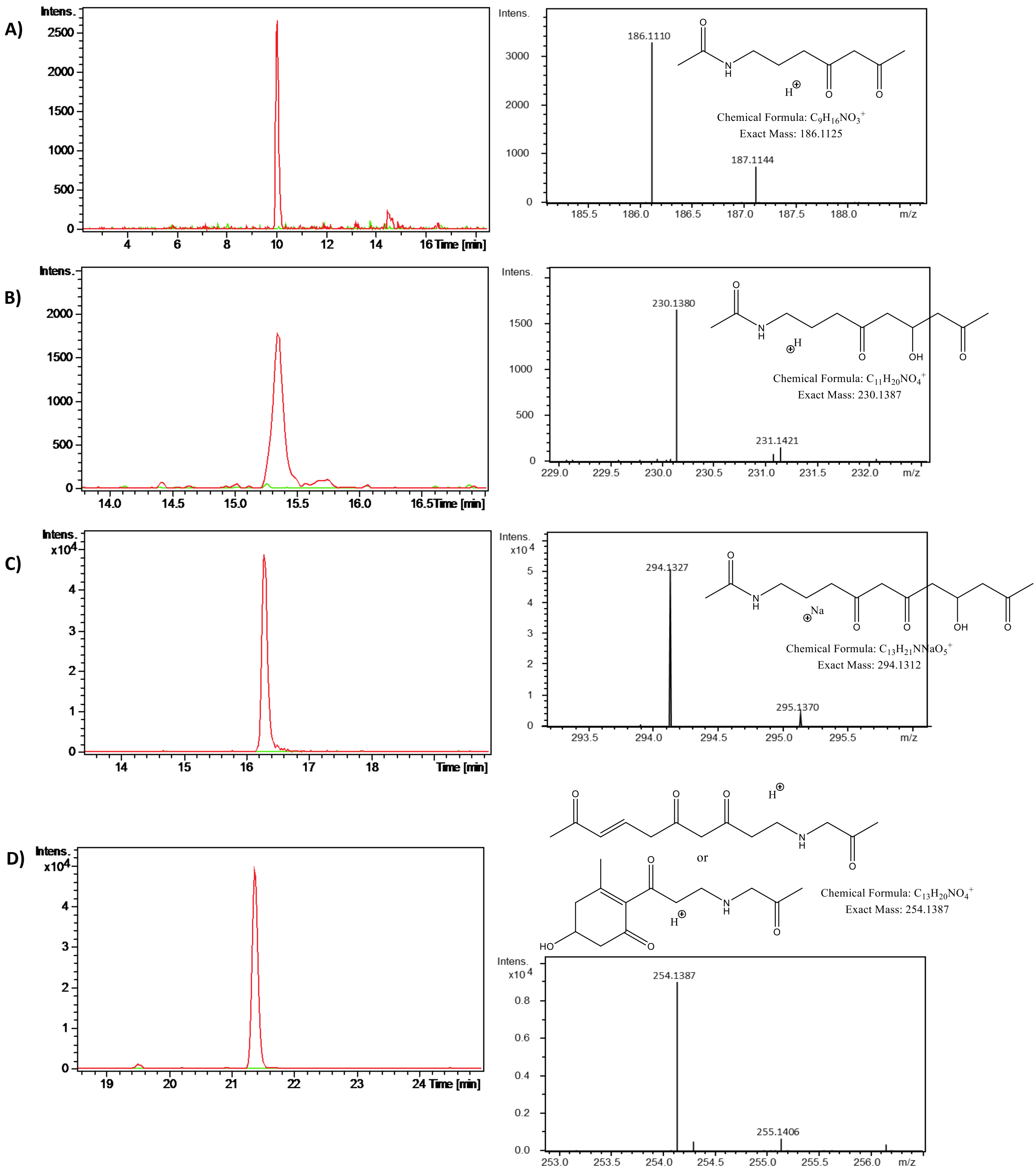

Figure 6S: LC-HRMS analysis (MaXis Impact) of the organic extracts of *P. patulum* grown in the absence (green) and in the presence (red) of **5** (final concentration 10 mM). $[M+H]^+$ extracted ion traces (left) and high resolution massed (right) are shown for: **A)** a putative diketide (R_t = 10.1 min), **B)** a reduced triketide (R_t = 16.3 min), a hydroxylated (**C**) and dehydrated (**D**) tetraketide (R_t = 16.3 and 21.4 min respectively).

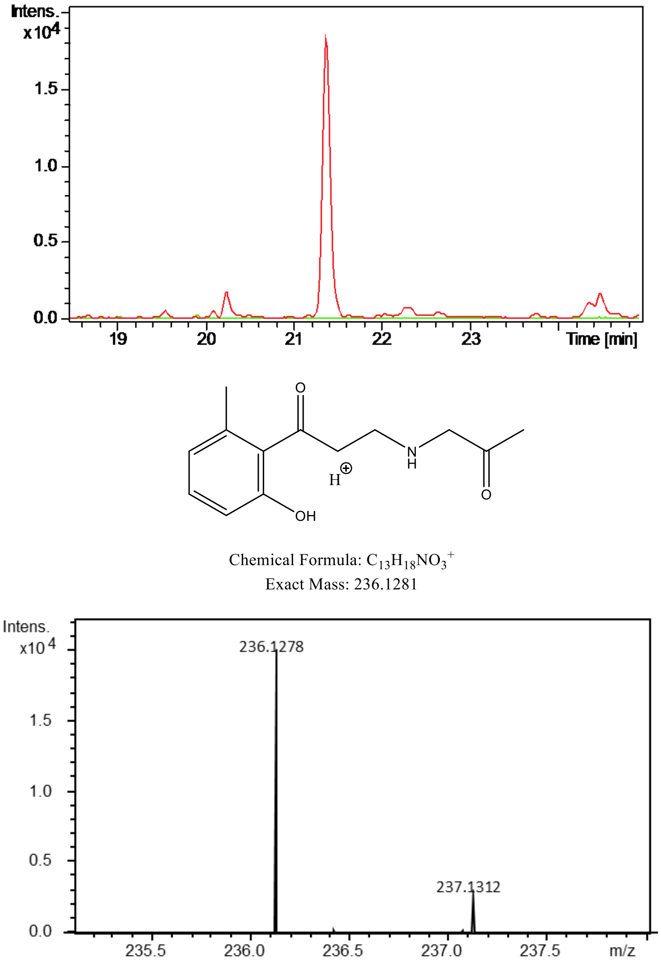

Figure 7S: LC-HRMS analysis (MaXis Impact) of the organic extracts of *P. patulum* grown in the absence (green) and in the presence (red) of **5** (final concentration 10 mM). $[M+H]^+$ extracted ion traces (top) and high resolution mass (bottom) are shown for a putative aromatized tetraketide of *m/z* 236. Given that its retention time is identical to that of a dehydrated tetraketide (21.4 min, Figure 6S), we are unable to confirm whether this is a distinct aromatized species.

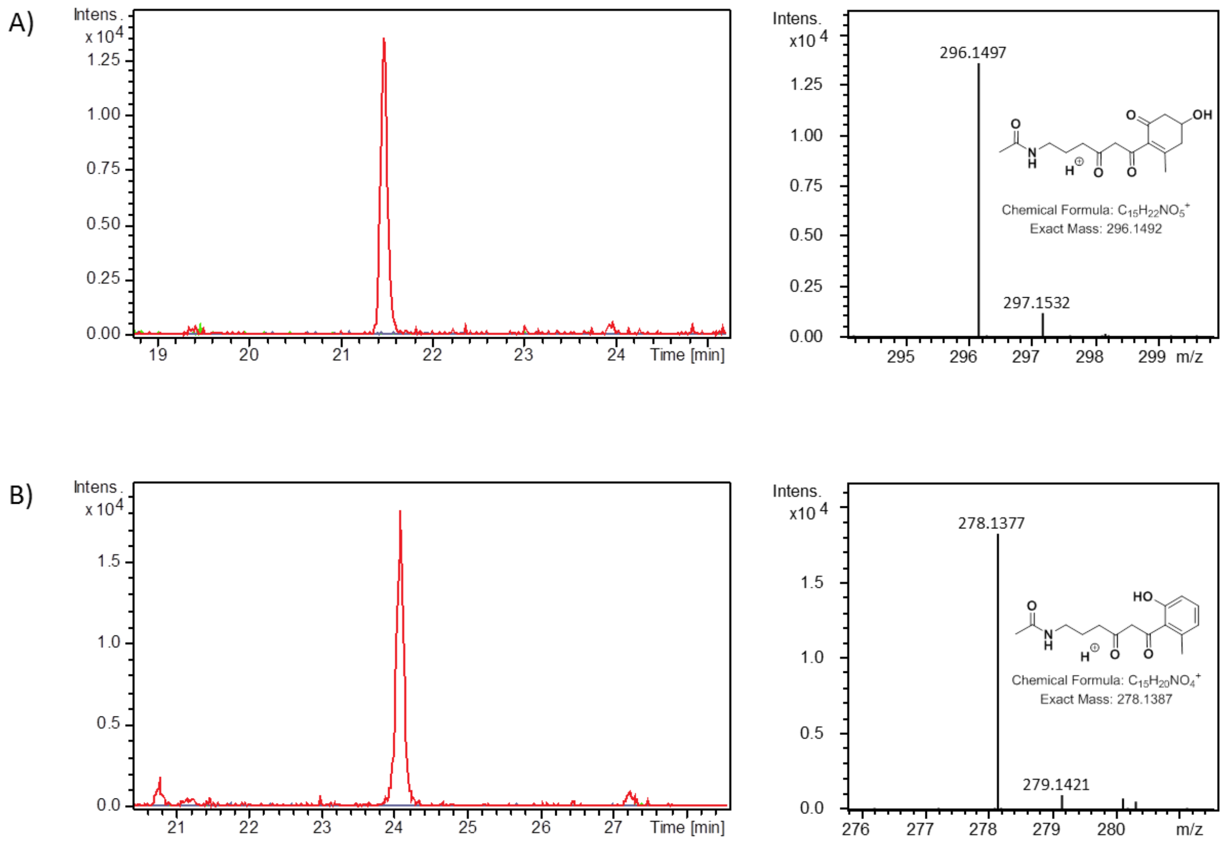

Figure 8S: LC-HRMS analysis (MaXis Impact) of the organic extracts of *P. patulum* grown in the absence (green) and in the presence (red) of **5** (final concentration 10 mM). [M+H]+ extracted ion traces and high resolution masses are shown for putative dehydrated (**A**, Rt= 21.5 min) and aromatized (**B**, Rt= 24.1 min) pentaketides.

2.4.3 Intermediate capture by probe 6 (methyl 3-oxo-6-(pent-4-ynamido) hexanoate)[1b]

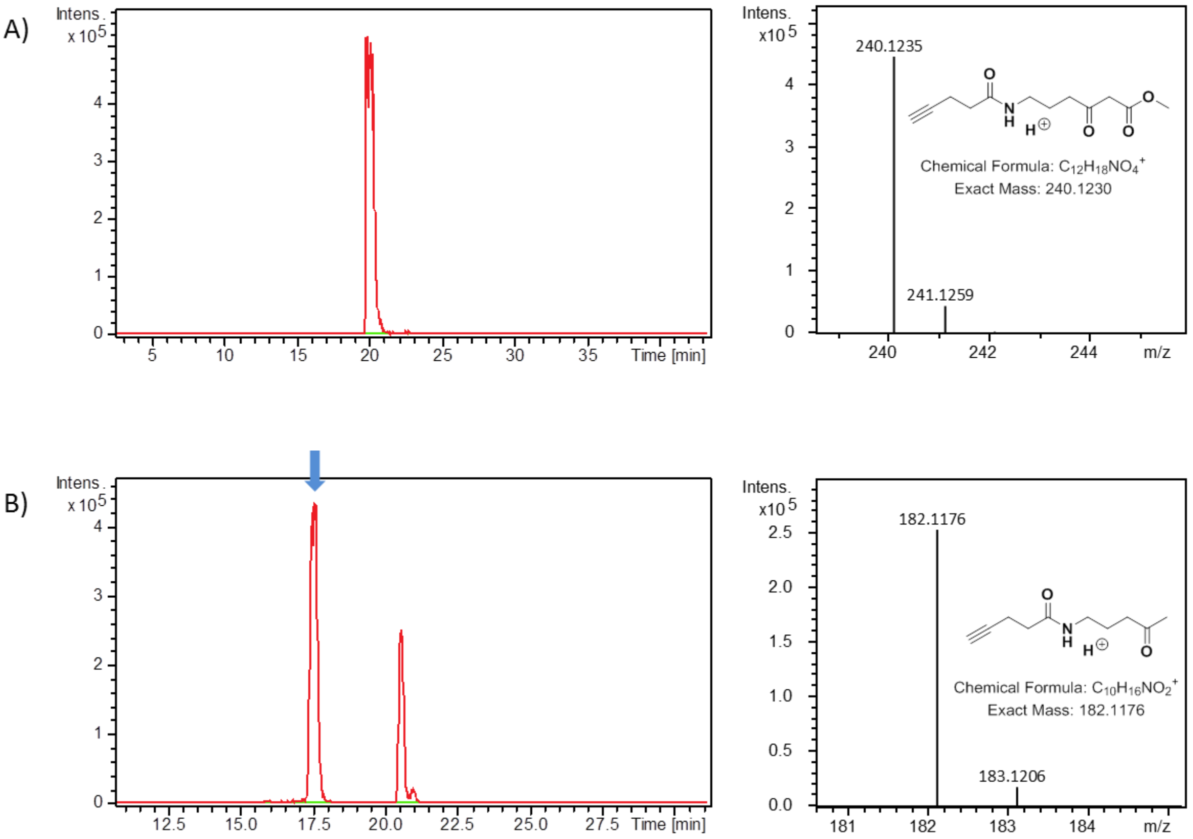

Figure 9S: LC-HRMS analysis of the organic extracts of *P. patulum* grown in the absence (green) and in the presence (red) of probe **6** (final concentration 10 mM): **A)** $[M+H]^+$ extracted ion trace (left) and high resolution mass (right) for **6** (R_t = 20.3 min); **B)** $[M+H]^+$ extracted ion trace (left) and high resolution mass (right) for *N*-(4-oxopentyl)pent-4-ynamide (R_t = 17.6 min), resulting from *in vivo* hydrolysis and decarboxylation of **6** (MaXis Impact analyses).

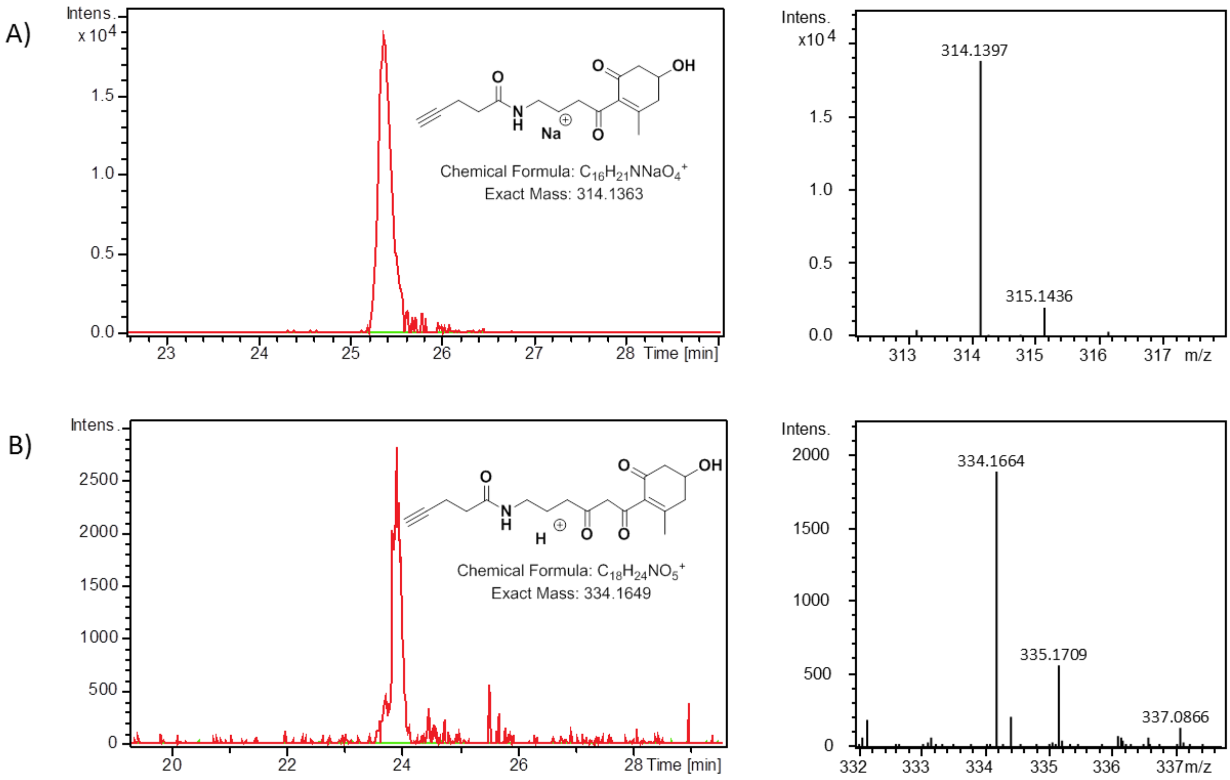

Figure 10S: LC-HRMS analysis (MaXis Impact) of the organic extracts of *P. patulum* grown in the absence (green) and in the presence (red) of **6** (final concentration 10 mM). $[M+H]^+$ extracted ion traces and high resolution masses are shown for a putative hydroxy tetraketide (R_t=25.4 min) and dehydrated pentaketide (R_t=23.9 min).

2.4.4 Intermediate capture by probe 8 (methyl 6-decanamido-3-oxohexanoate)[1b]

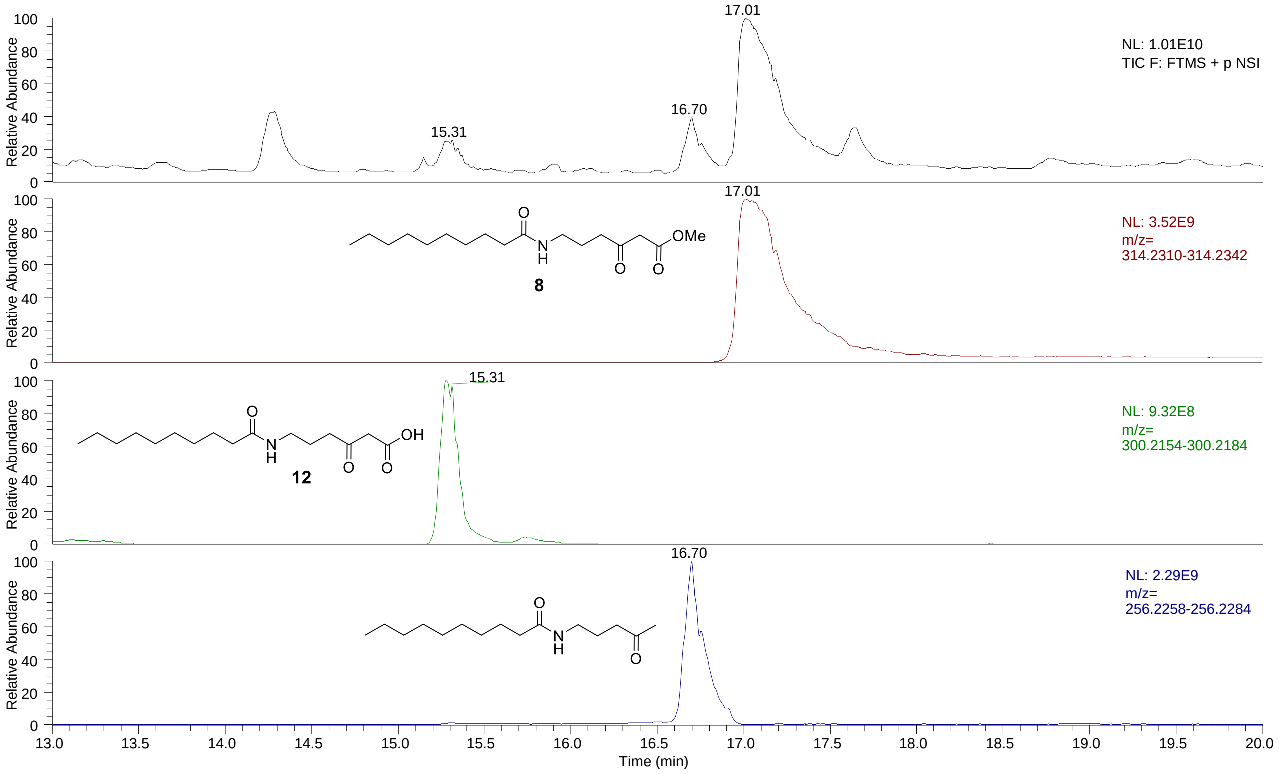

Figure 11S: LC-HRMS analysis (Orbitrap Fusion) of the organic extracts of *P. patulum* grown in the presence of probe **8** (final concentration 10 mM). TIC and $[M+H]^+$ extracted ion traces for methyl 6-decanamido-3-oxohexanoate **8**,[1b] the hydrolyzed probe **12** and its decarboxylation product *N*-(4-oxopentyl)decanamide are given.

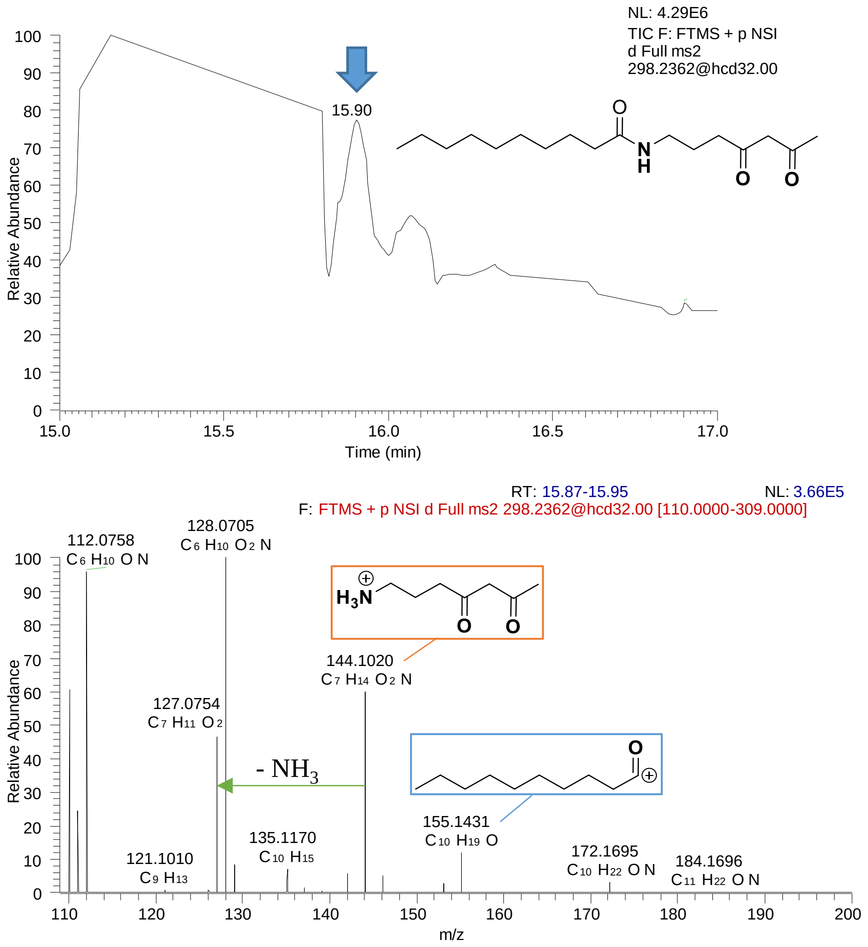

Figure 12S: LC-HRMS analysis (Orbitrap Fusion) of the organic extracts of *P. patulum* grown in the presence of **8:** detection (top) and fragmentation (bottom) of a putative diketide (with putative fragment structural assignment).

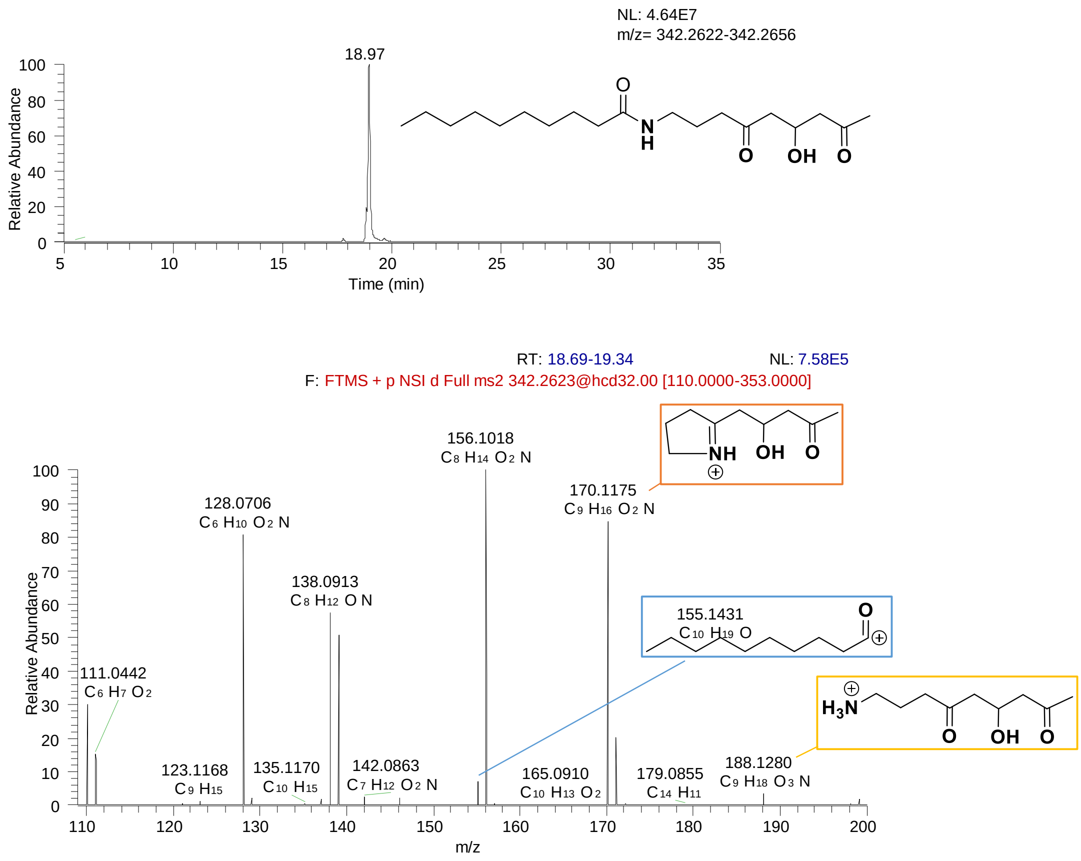

Figure 13S: LC-HRMS analysis (Orbitrap Fusion) of the organic extracts of *P. patulum* grown in the presence of **8:** [M+H]$^+$ extracted ion trace (top) and fragmentation (bottom) of a putative reduced triketide (with putative fragment structural assignment).

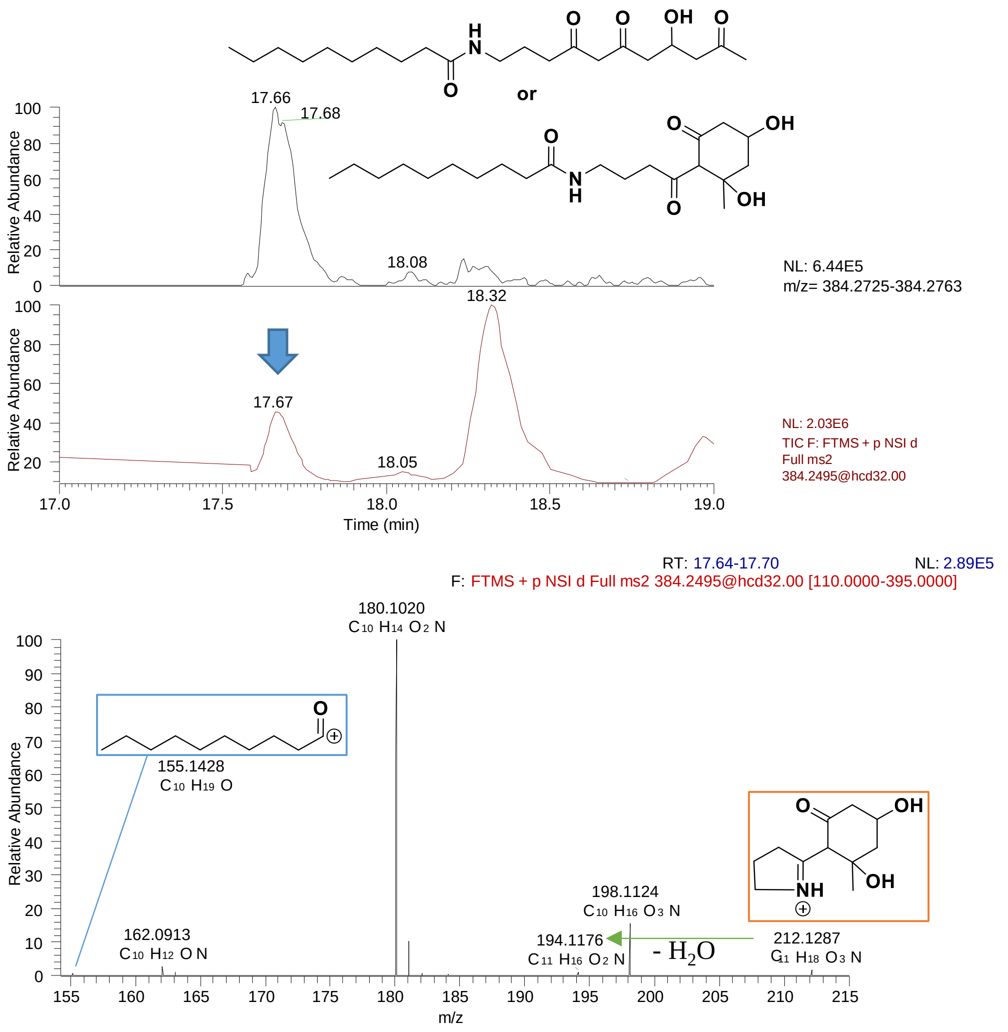

Figure 14S: LC-HRMS analysis (Orbitrap Fusion) of the organic extracts of *P. patulum* grown in the presence of **8:** $[M+H]^+$ extracted ion trace (top) and fragmentation (bottom) of a putative hydroxy tetraketide (with putative fragment structural assignment).

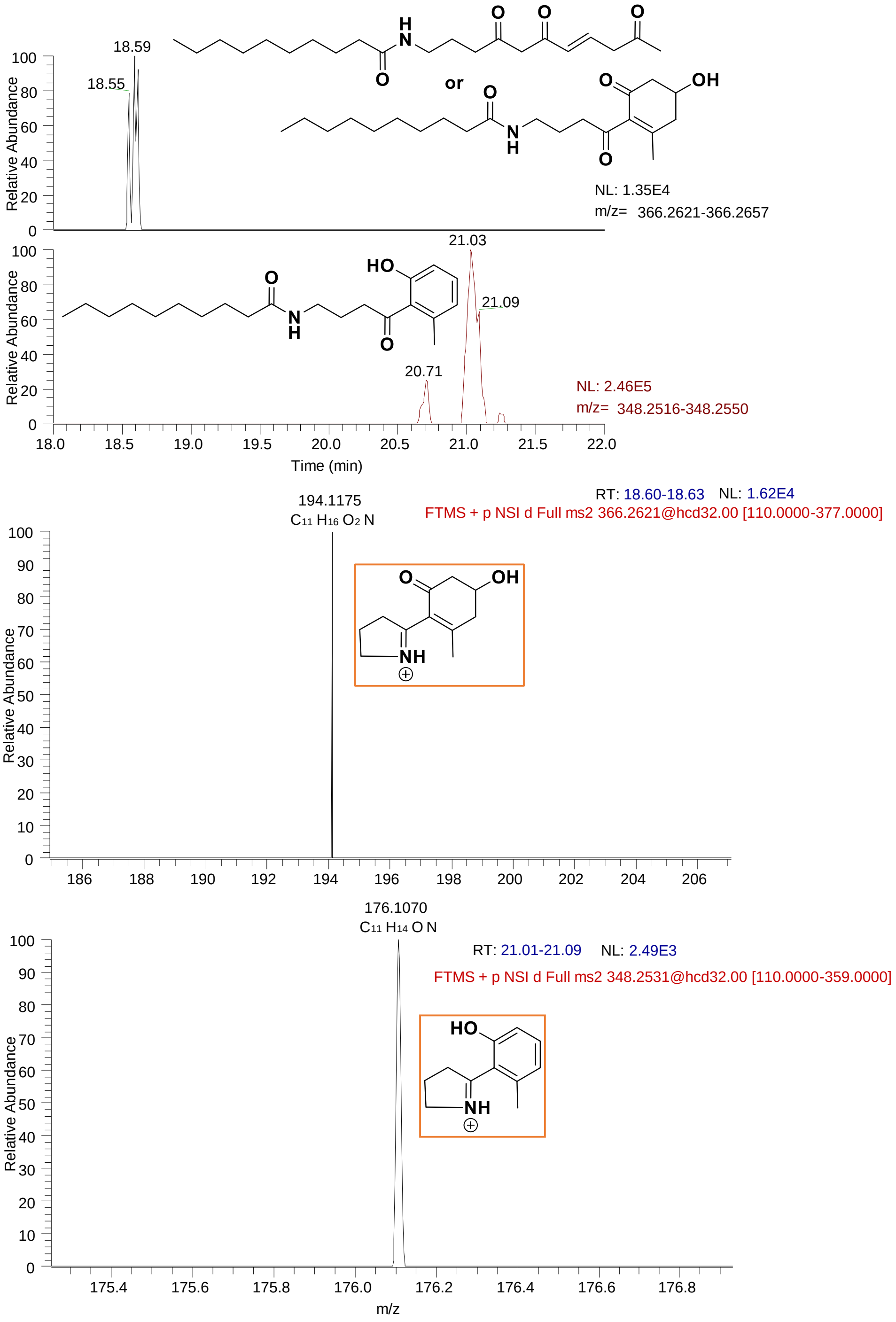

Figure 15S: LC-HRMS analysis (Orbitrap Fusion) of the organic extracts of *P. patulum* grown in the presence of **8:** $[M+H]^+$ extracted ion traces (top) and fragmentation expansions (middle and bottom) of dehydrated and aromatized tetraketides (with putative fragment structural assignment).

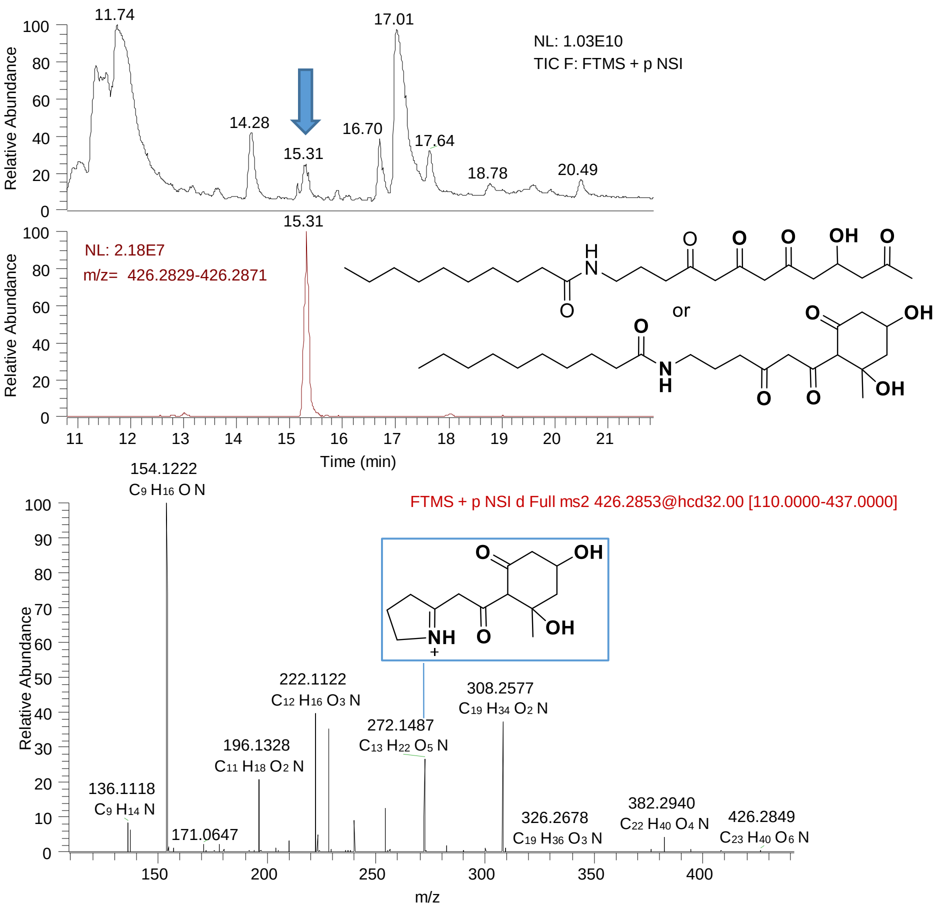

Figure 16S: LC-HRMS analysis (Orbitrap Fusion) of the organic extracts of *P. patulum* grown in the presence of **8:** TIC and $[M+H]^+$ extracted ion traces (top), followed by fragmentation (bottom, full view) of a hydroxyl pentaketide (with putative fragment structural assignment).

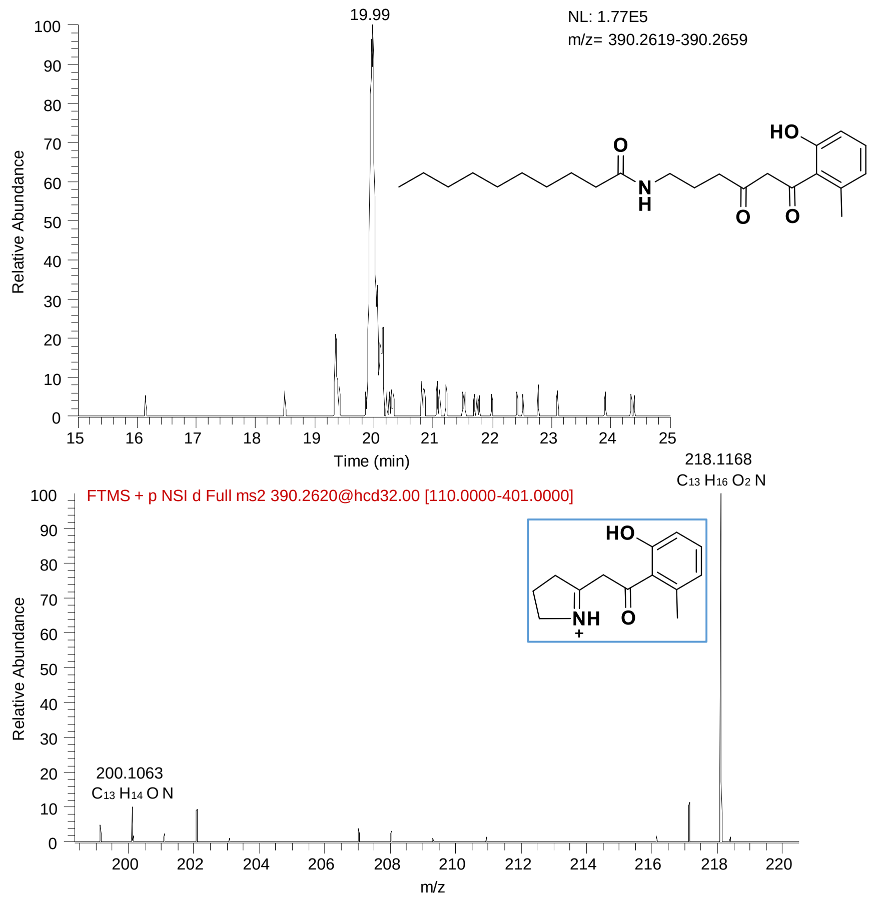

Figure 17S: LC-HRMS analysis (Orbitrap Fusion) of the organic extracts of *P. patulum* grown in the presence of **8:** $[M+H]^+$ extracted ion trace (top) and fragmentation (expansion, bottom) for a putative aromatized pentaketide (with putative fragment structural assignment).

2.5 Capture of 6-MSAS intermediates from *E. coli* BAP1 pKOS007-109 *via* chain termination probes

2.5.1 Intermediate capture by probes 4a and 4b

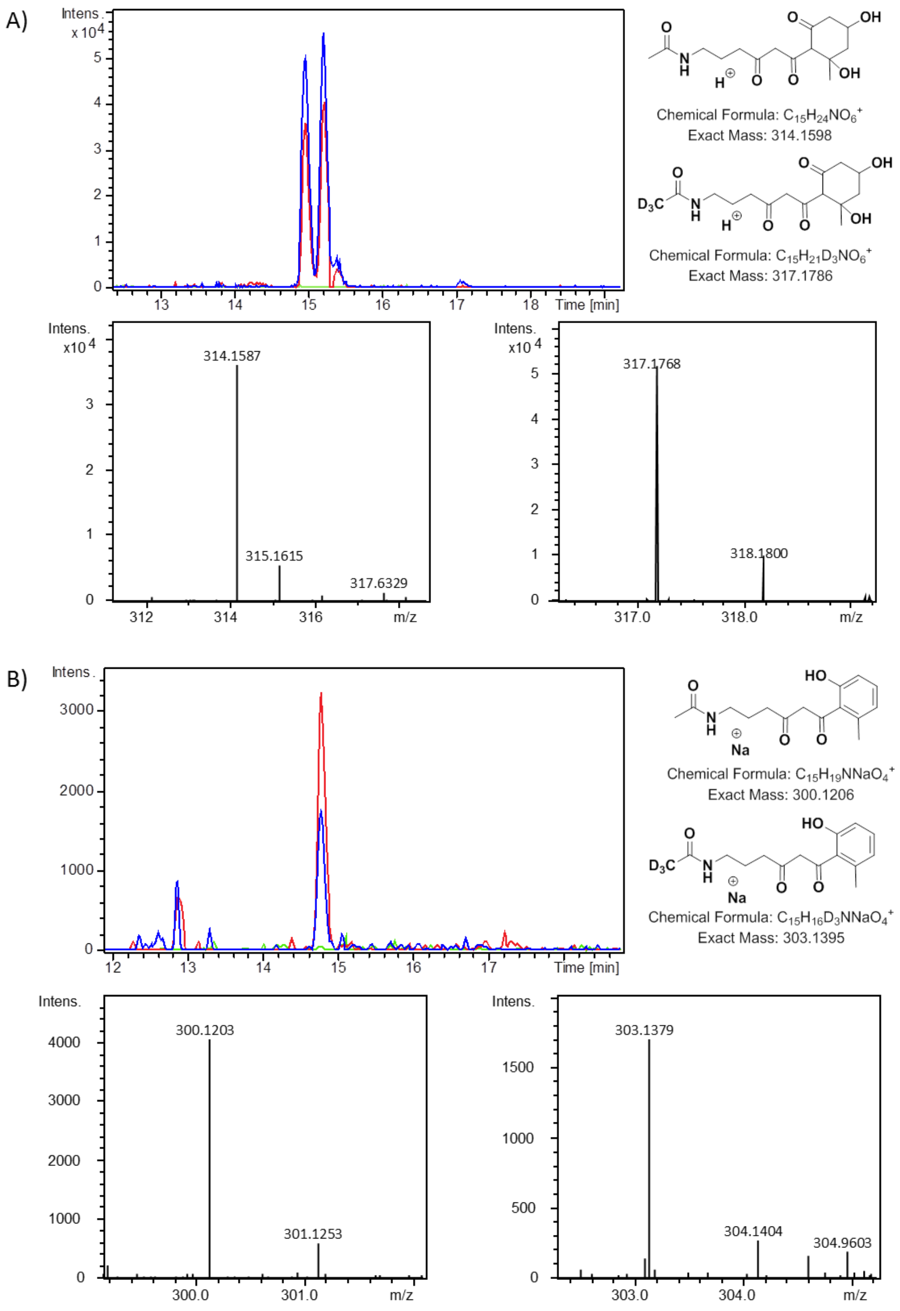

Figure 18S: LC-HRMS analysis (MaXis Impact) of the organic extracts of *E. coli* BAP1 pKOS007-109 grown in the absence (green) and in the presence of **4a** (red) and **4b** (blue)(final concentration 10 mM). $[M+H]^+$ extracted ion traces and high resolution masses are shown for putative hydroxy (**A**) and aromatized (**B**) pentaketides. Note the presence of multiple peaks, which may arise from isomerization and/or cyclization.

2.5.2 Intermediate capture by probe 6

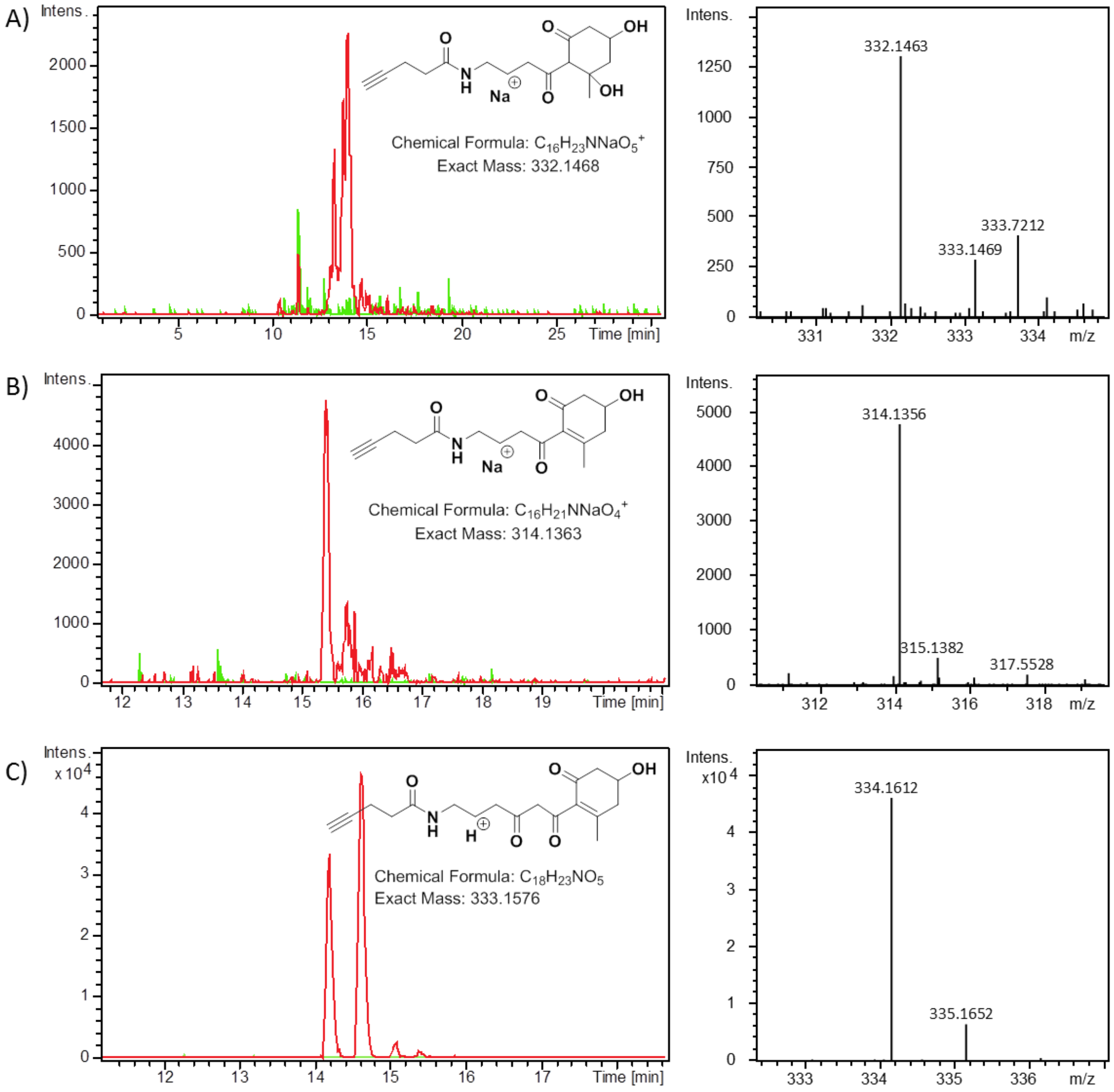

Figure 19S: LC-HRMS analysis (MaXis Impact) of the organic extracts of *E. coli* BAP1 pKOS007-109 grown in the absence (green) and in the presence (red) of **6** (blue)(final concentration 10 mM). $[M+H]^+$ extracted ion traces and high resolution masses are shown for putative hydroxy (**A**) and dehydrated (**B**) tetraketides, as well as for a dehydrated pentaketide (**C**). Note the presence of multiple peaks, which may arise from isomerization and/or cyclization.

2.5.3 Intermediate capture by probe 8

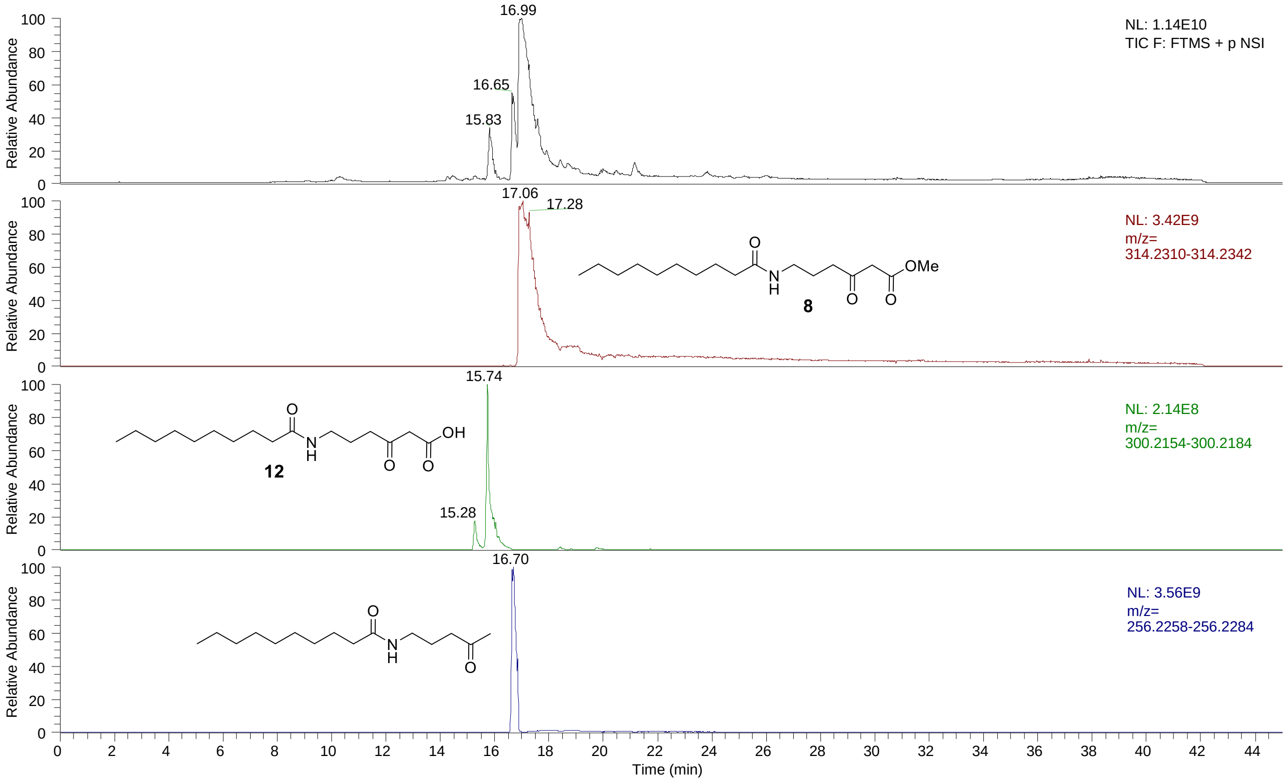

Figure 20S: LC-HRMS analysis (Orbitrap Fusion) of the organic extracts of *E. coli* BAP1 pKOS007-109 grown in the presence of probe **8** (final concentration 10 mM). TIC and $[M+H]^+$ extracted ion traces for **8***,* the hydrolyzed probe **12** and its decarboxylation product are given.

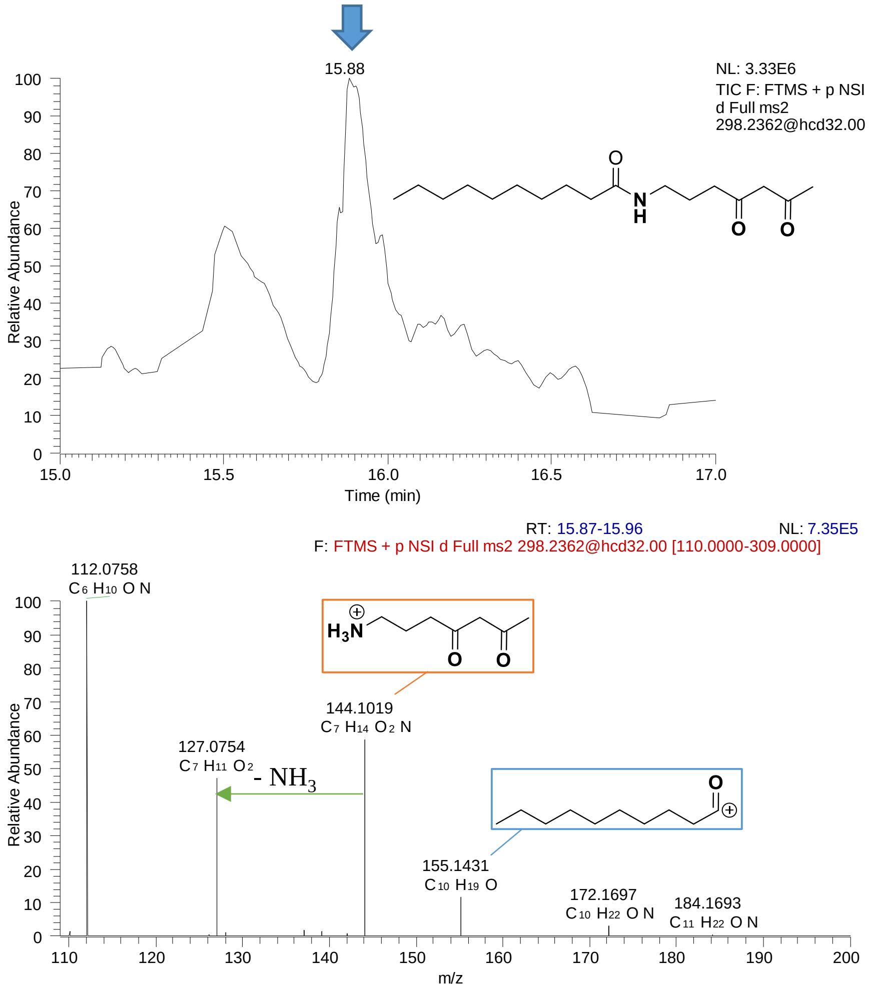

Figure 21S: LC-HRMS analysis (Orbitrap Fusion) of the organic extracts of *E. coli* BAP1 pKOS007-109 grown in the presence of **8:** detection (top) and fragmentation (bottom) of a putative diketide (with putative fragment structural assignment).

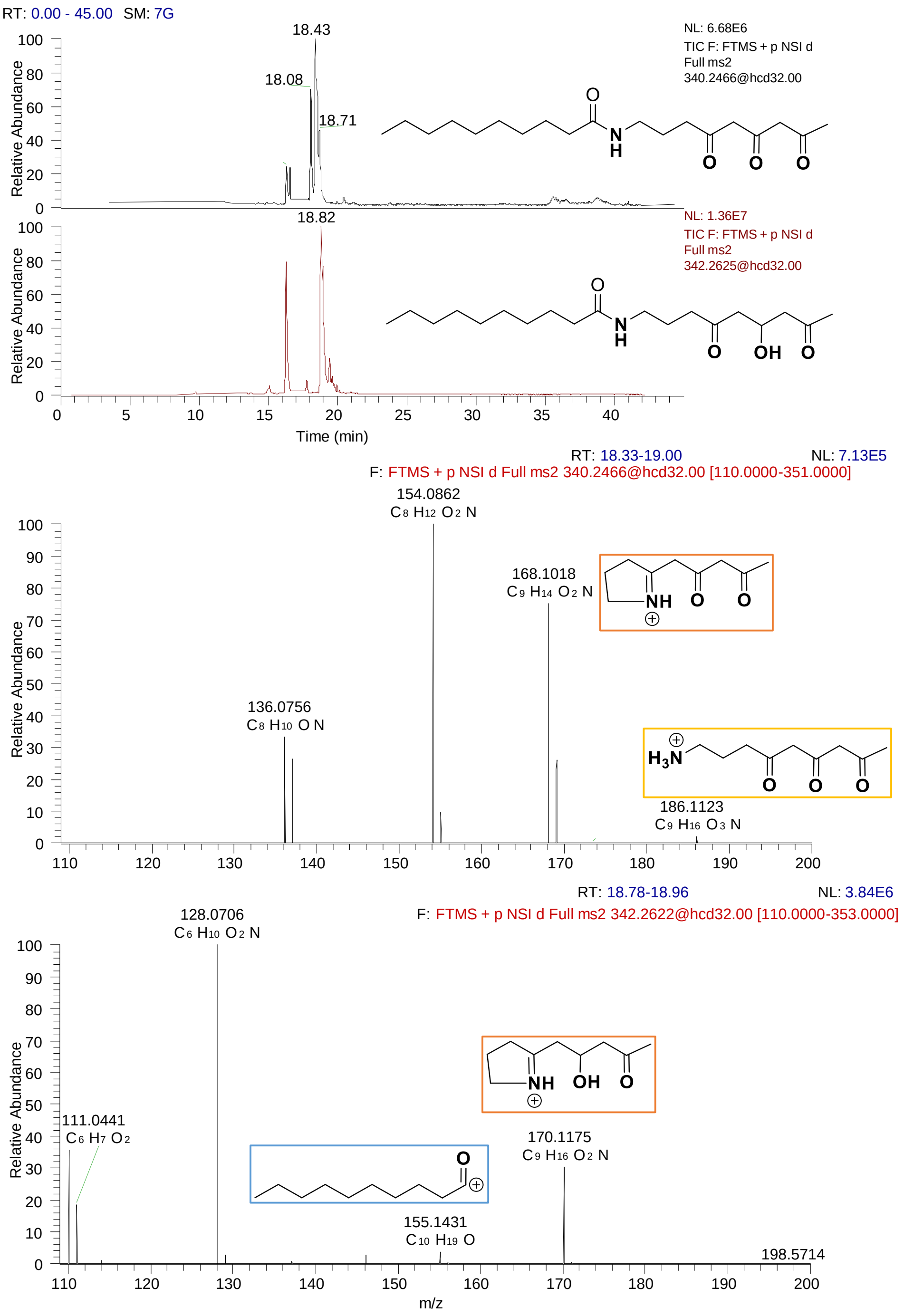

Figure 22S: LC-HRMS analysis (Orbitrap Fusion) of the organic extracts of *E. coli* BAP1 pKOS007-109 grown in the presence of **8**: $[M+H]^+$ extracted ion traces (top) and fragmentations (middle and bottom) of putative triketides (with putative fragment structural assignment).

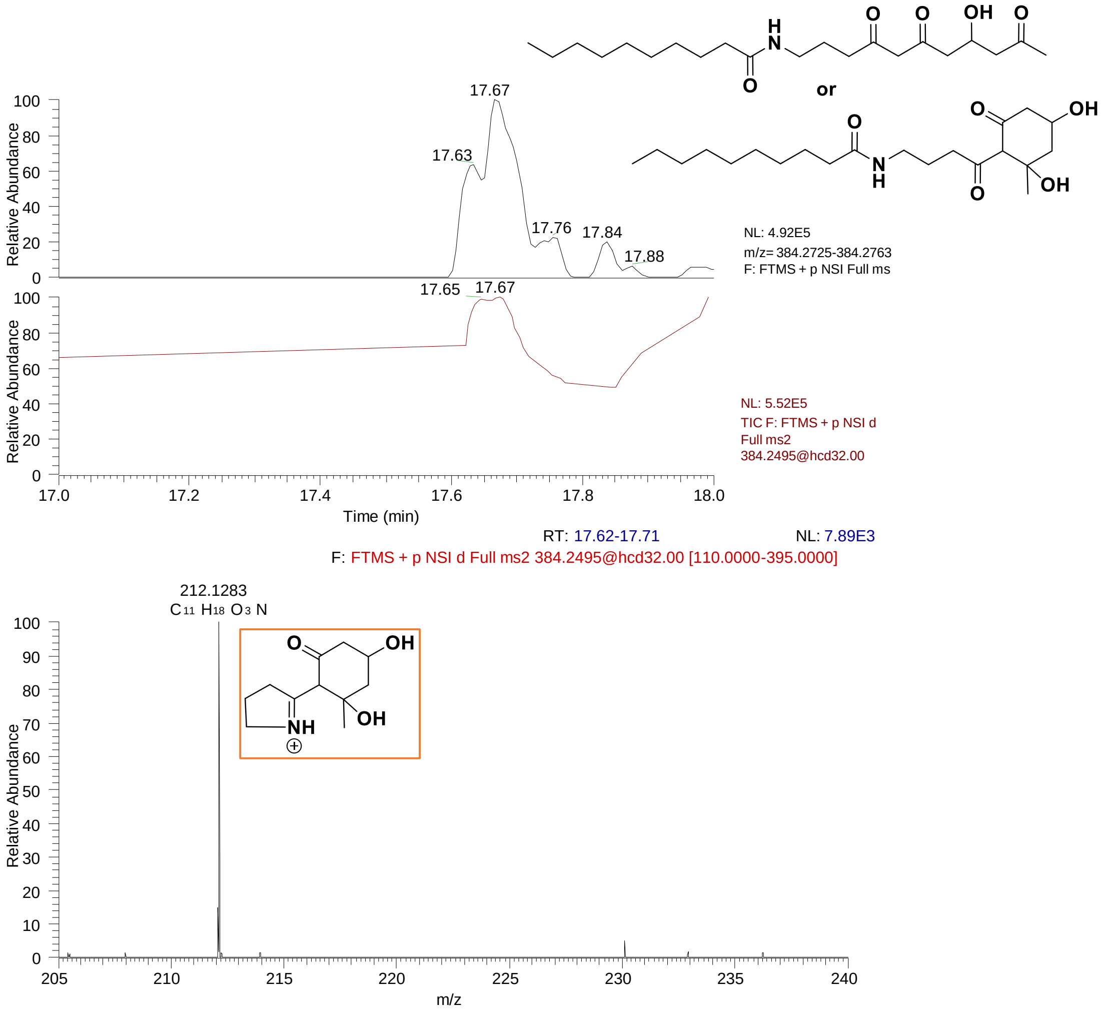

Figure 23S: LC-HRMS analysis (Orbitrap Fusion) of the organic extracts of *E. coli* BAP1 pKOS007-109 grown in the presence of **8**: $[M+H]^+$ extracted ion traces (top) and fragmentation expansion (bottom) of a putative hydroxy tetraketide (expansion, with putative fragment structural assignment; diagnostic *m/z* 155 fragment also present).

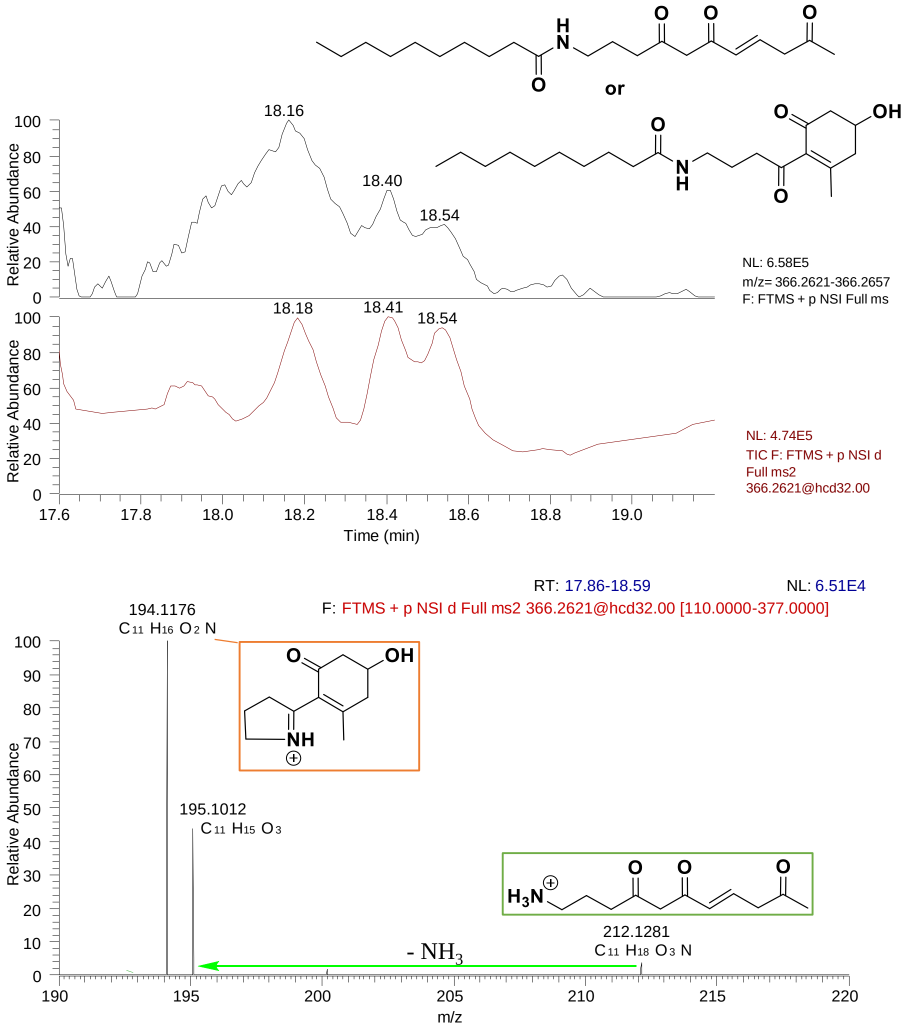

Figure 24S: LC-HRMS analysis (Orbitrap Fusion) of the organic extracts of *E. coli* BAP1 pKOS007-109 grown in the presence of **8:** $[M+H]^+$ extracted ion traces (top) and fragmentation (bottom) of a putative dehydrated tetraketide (expansion, with putative fragment structural assignment). Note the presence of multiple peaks for this species, which may arise from double bond isomerization and/or cyclization.

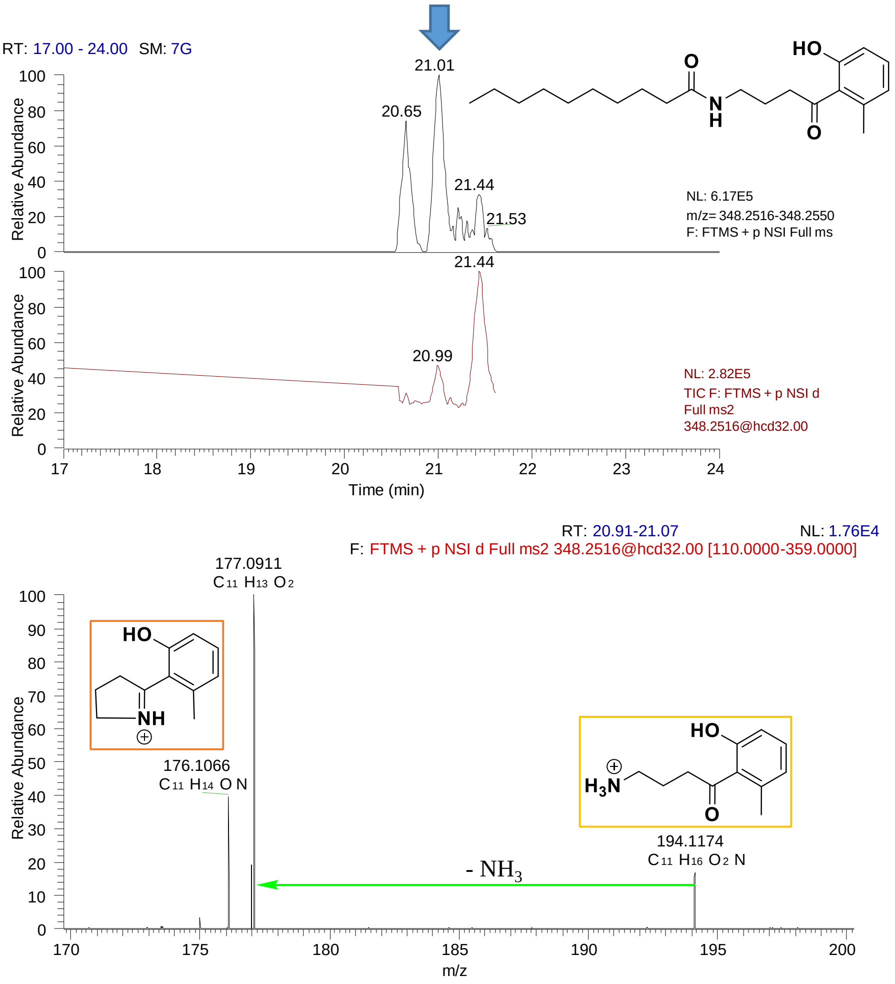

Figure 25S: LC-HRMS analysis (Orbitrap Fusion) of the organic extracts of *E. coli* BAP1 pKOS007-109 grown in the presence of **8**: $[M+H]^+$ extracted ion traces (top) and fragmentation expansion (bottom) of a putative aromatized tetraketide (with putative fragment structural assignment; diagnostic *m/z* 155 fragment also present).

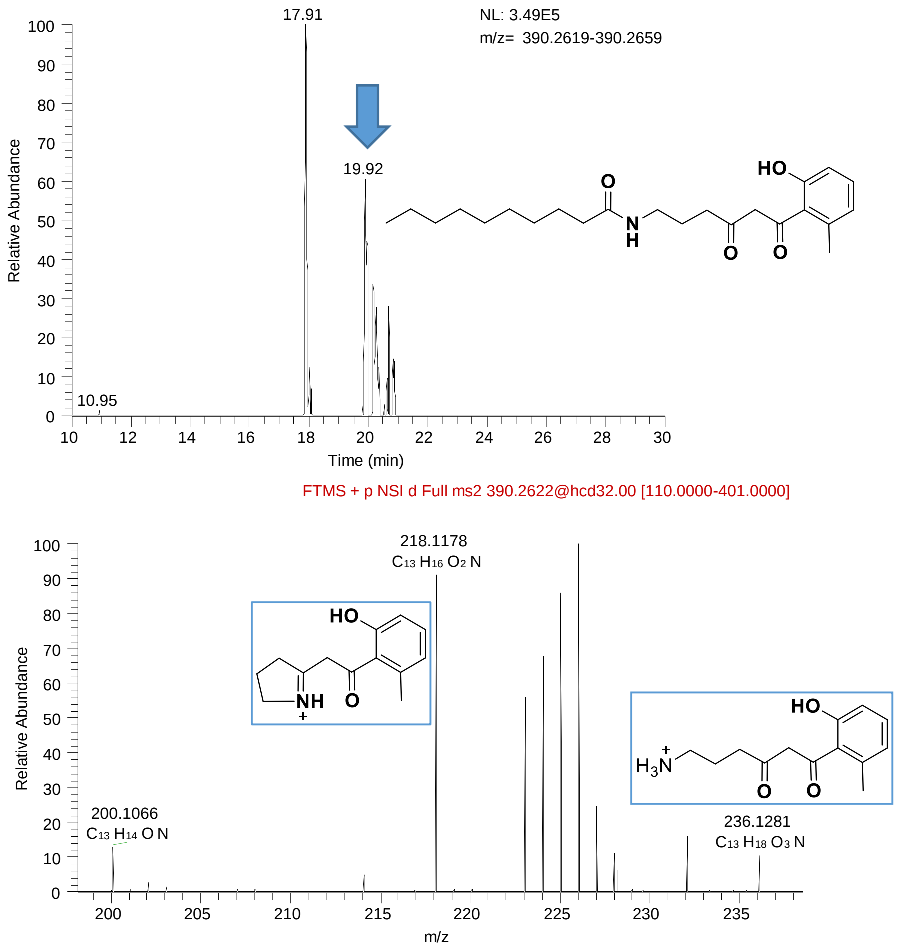

Figure 26S: LC-HRMS analysis (Orbitrap Fusion) of the organic extracts of *E. coli* BAP1 pKOS007-109 grown in the presence of **8**: $[M+H]^+$ extracted ion trace (top) and fragmentation (bottom, expansion) of a putative aromatized pentaketide (with putative fragment structural assignment).

2.6 Capture of 6-MSAS from *S. antibioticus* DSM40725 *via* chain termination probes

2.6.1 Intermediate capture by probe 8

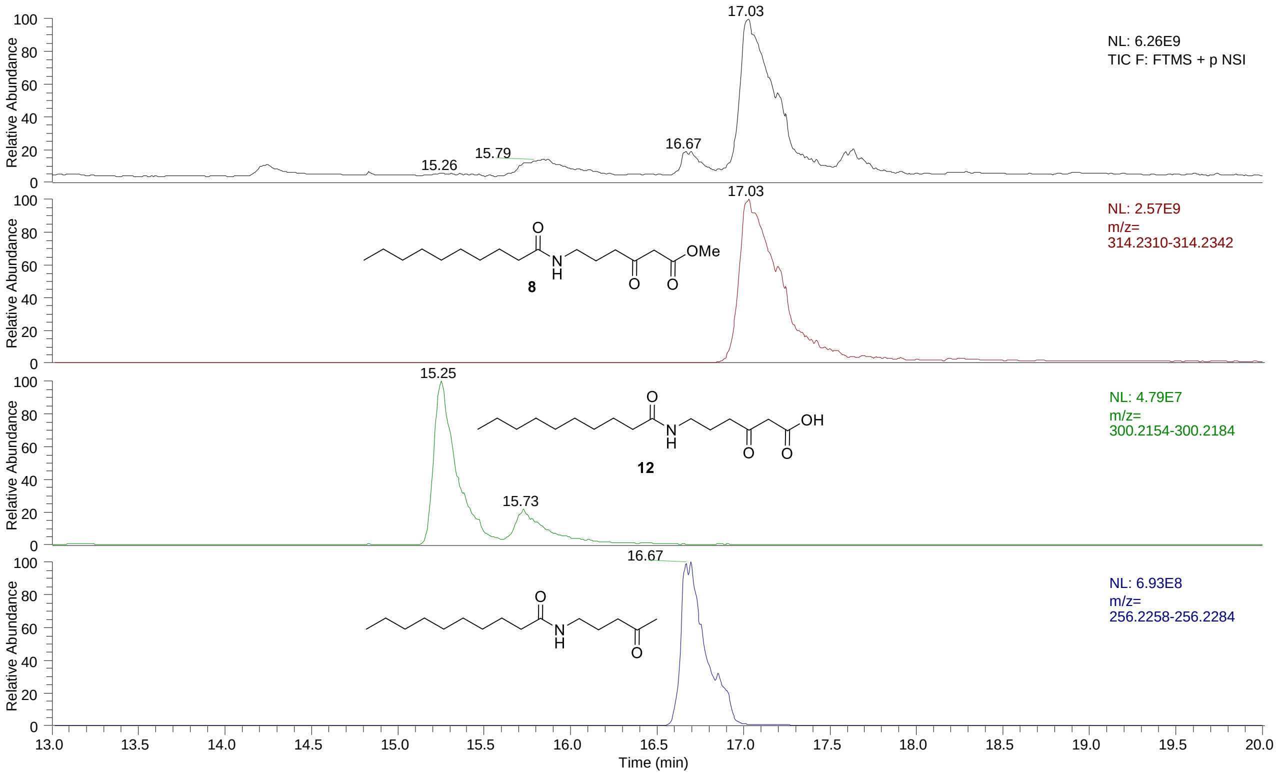

Figure 27S: LC-HRMS analysis (Orbitrap Fusion) of the organic extracts of *S. antibioticus* DSM40725 grown in the presence of probe **8.** TIC and $[M+H]^+$ extracted ion traces for **8**, the hydrolyzed probe **12** and its decarboxylation product are given.

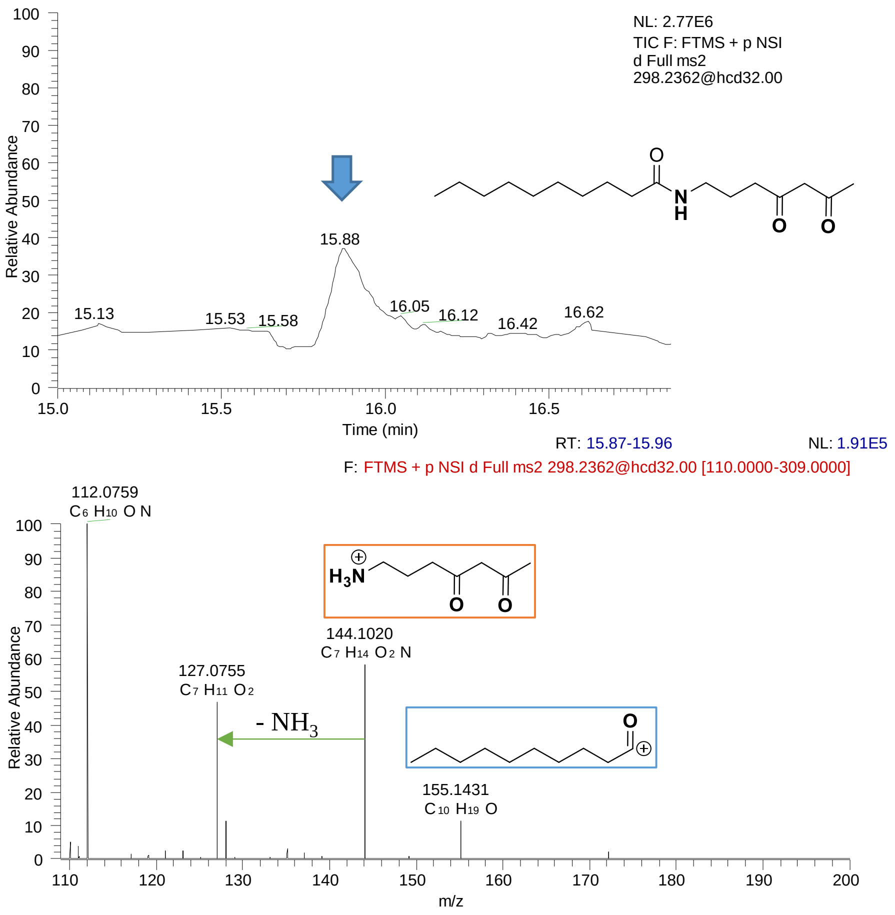

Figure 28S: LC-HRMS analysis (Orbitrap Fusion) of the organic extracts of *S. antibioticus* DSM40725 grown in the presence of **8:** high resolution mass (top) and fragmentation (bottom) of a putative diketide (with putative fragment structural assignment).

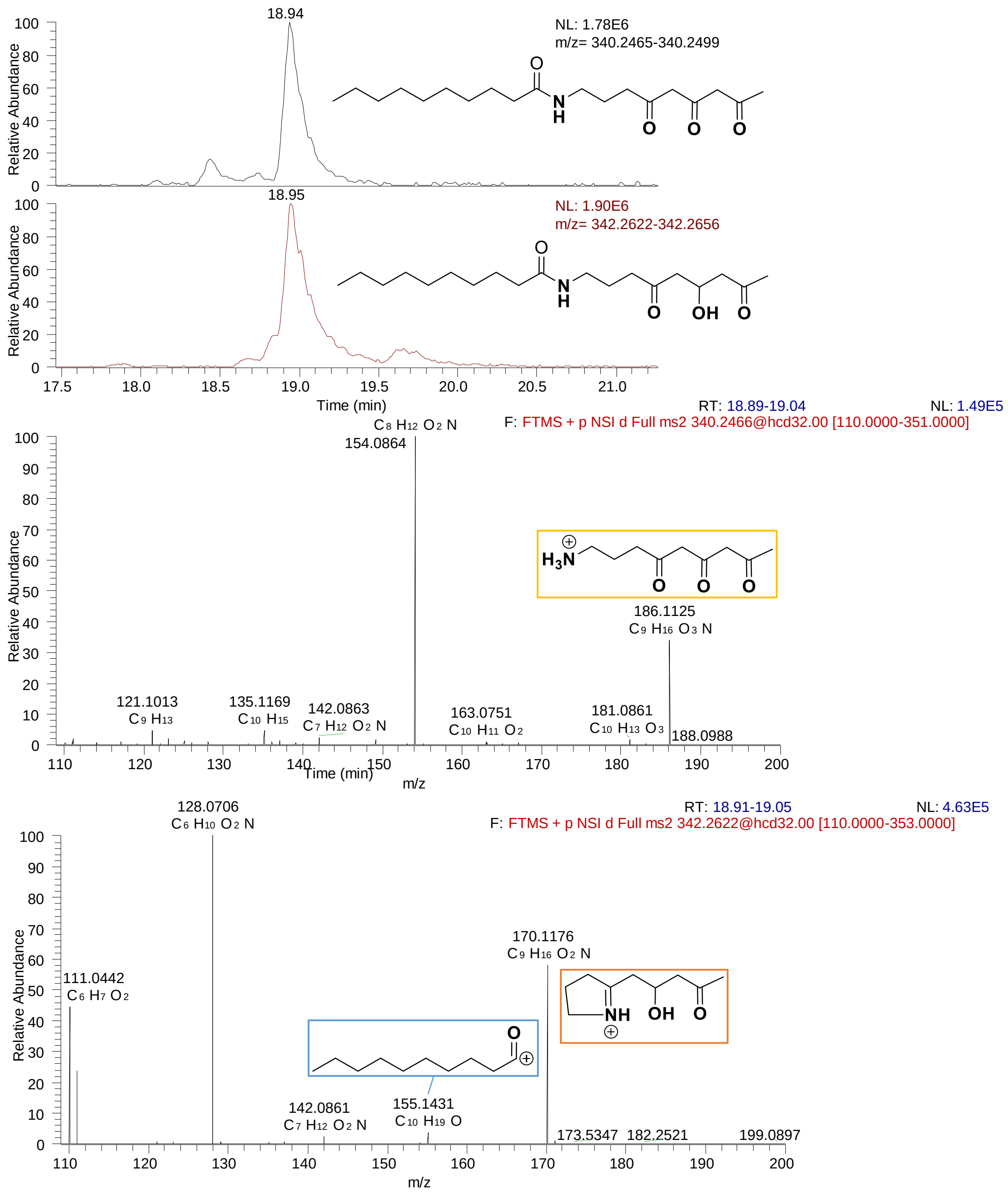

Figure 29S: LC-HRMS analysis (Orbitrap Fusion) of the organic extracts of *S. antibioticus* DSM40725 grown in the presence of **8**: $[M+H]^+$ extracted ion traces (top) and fragmentation (middle and bottom) of the putative triketides (with putative fragment structural assignment).

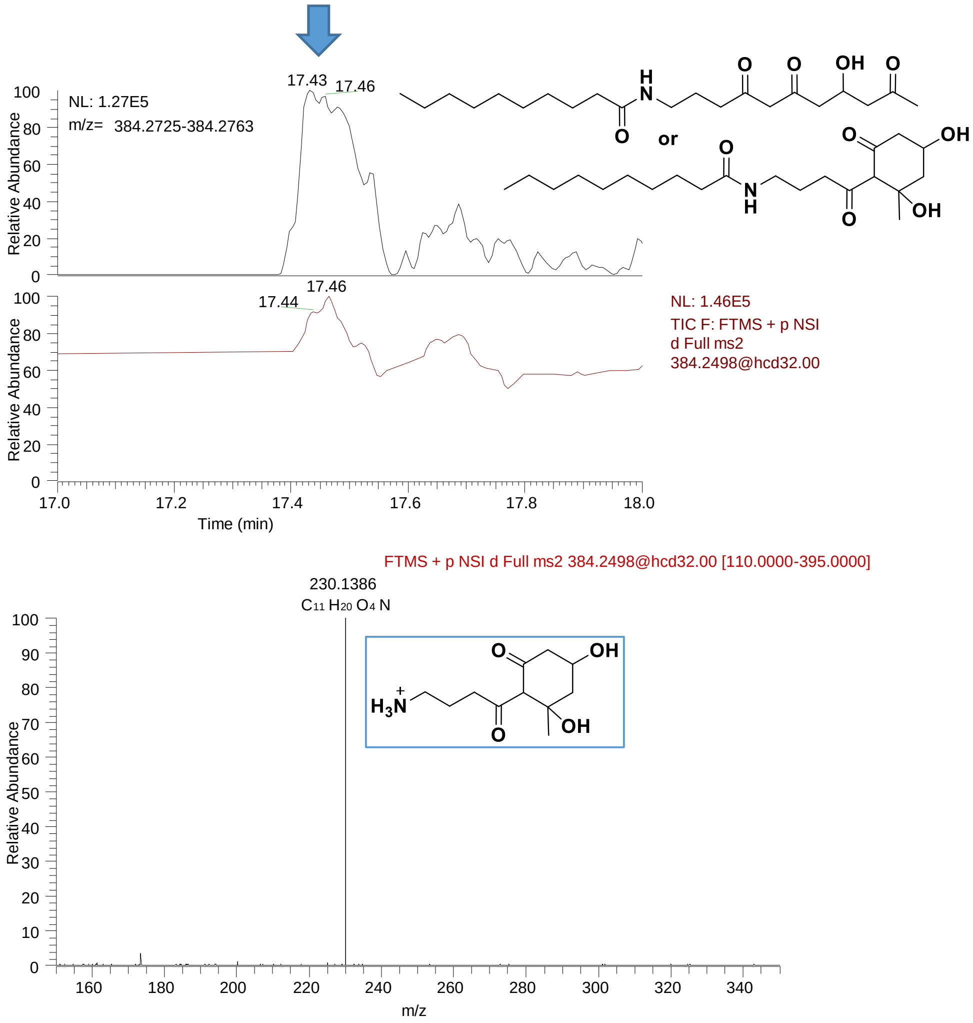

Figure 30S: LC-HRMS analysis (Orbitrap Fusion) of the organic extracts of *S. antibioticus* DSM40725 grown in the presence of **8**: $[M+H]^+$ and MS^2 extracted ion traces (top) and fragmentation (bottom) of a putative hydroxy tetraketide (with putative fragment structural assignment).

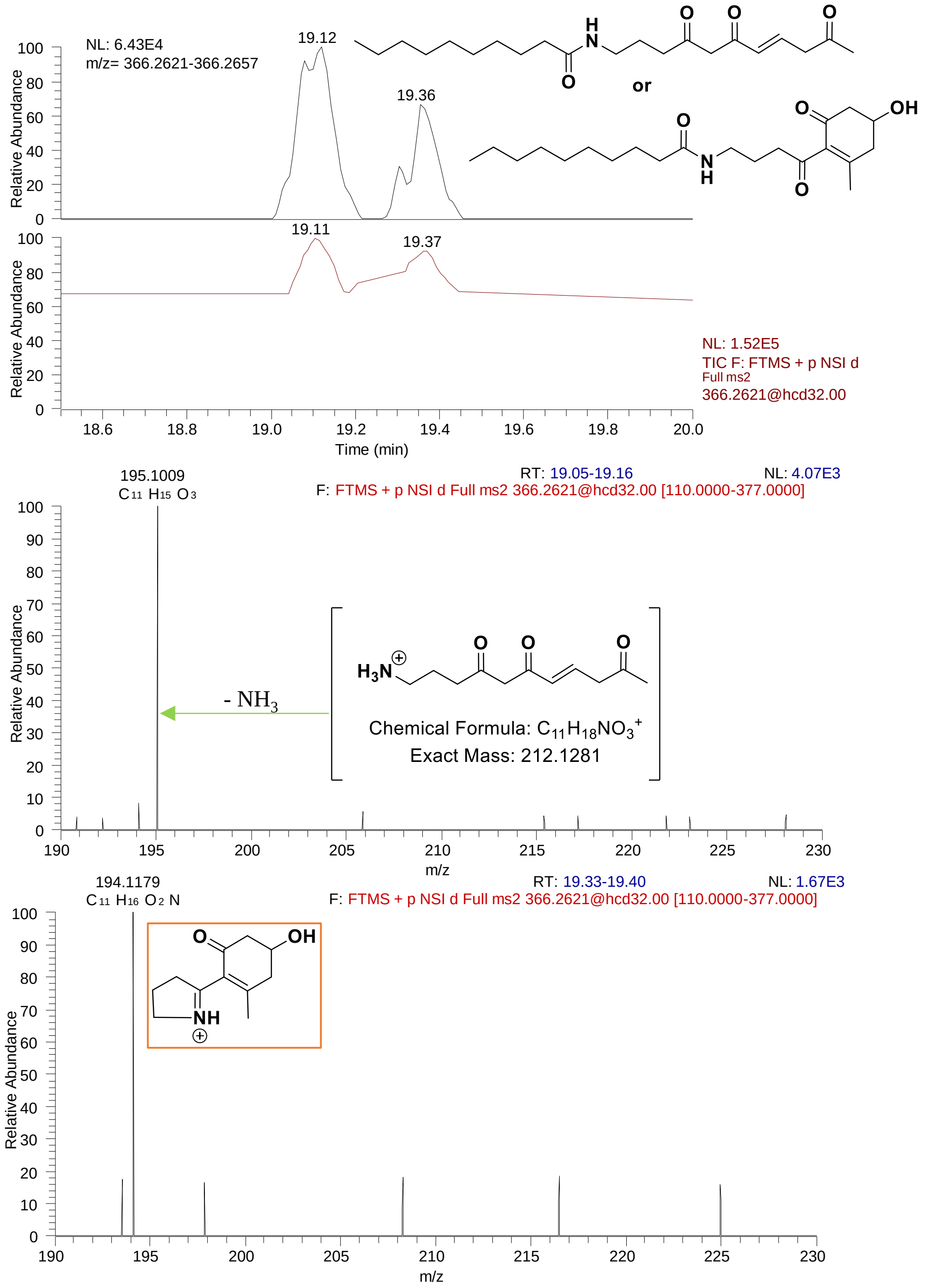

Figure 31S: LC-HRMS analysis (Orbitrap Fusion) of the organic extracts of *S. antibioticus* DSM40725 grown in the presence of **8**: $[M+H]^+$ extracted ion traces (top) and fragmentations (middle and bottom) of a putative dehydrated tetraketide (with putative fragment structural assignment; note the presence of multiple peaks).

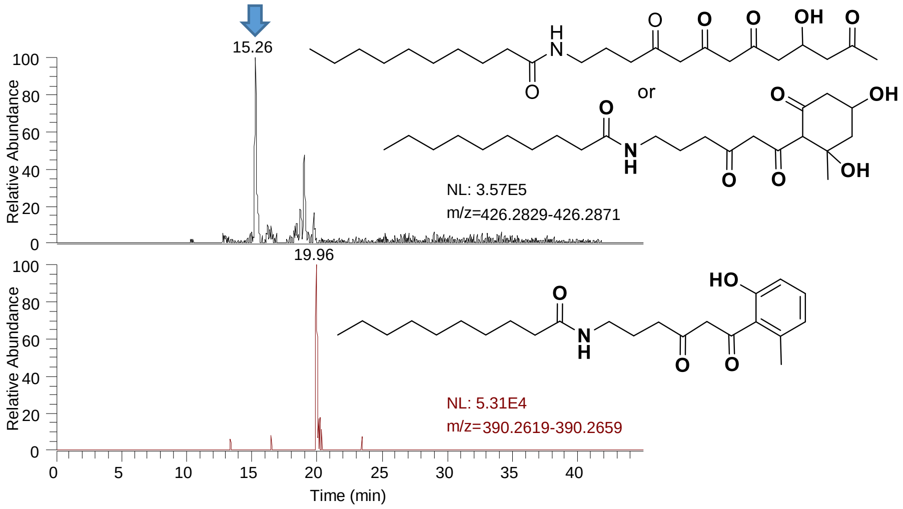

Figure 32S: LC-HRMS analysis (Orbitrap Fusion) of the organic extracts of *S. antibioticus* DSM40725 grown in the presence of **8**: $[M+H]^+$ extracted ion traces for a putative hydroxy and aromatized pentaketides, with identical retention times (and fragmentation patterns, not shown) displayed by analogous species identified in *P. patulum* and *E. coli* extracts (Figures 16, 17 and 26S).

2.6.2 Intermediate capture by probe 6

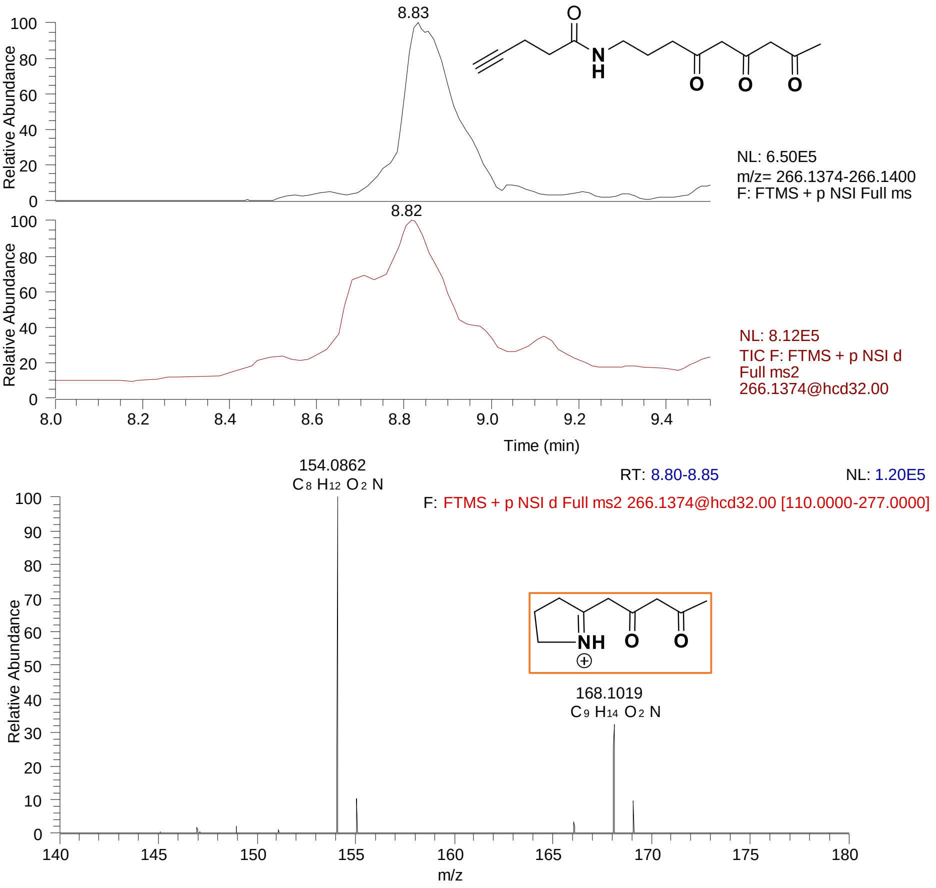

Figure 33S: LC-HRMS analysis (Orbitrap Fusion) of the organic extracts of *S. antibioticus* DSM40725 grown in the presence of **6**: $[M+H]^+$ extracted ion chromatograms (top) and fragmentation (bottom) of a putative triketide (with putative fragment structural assignment).

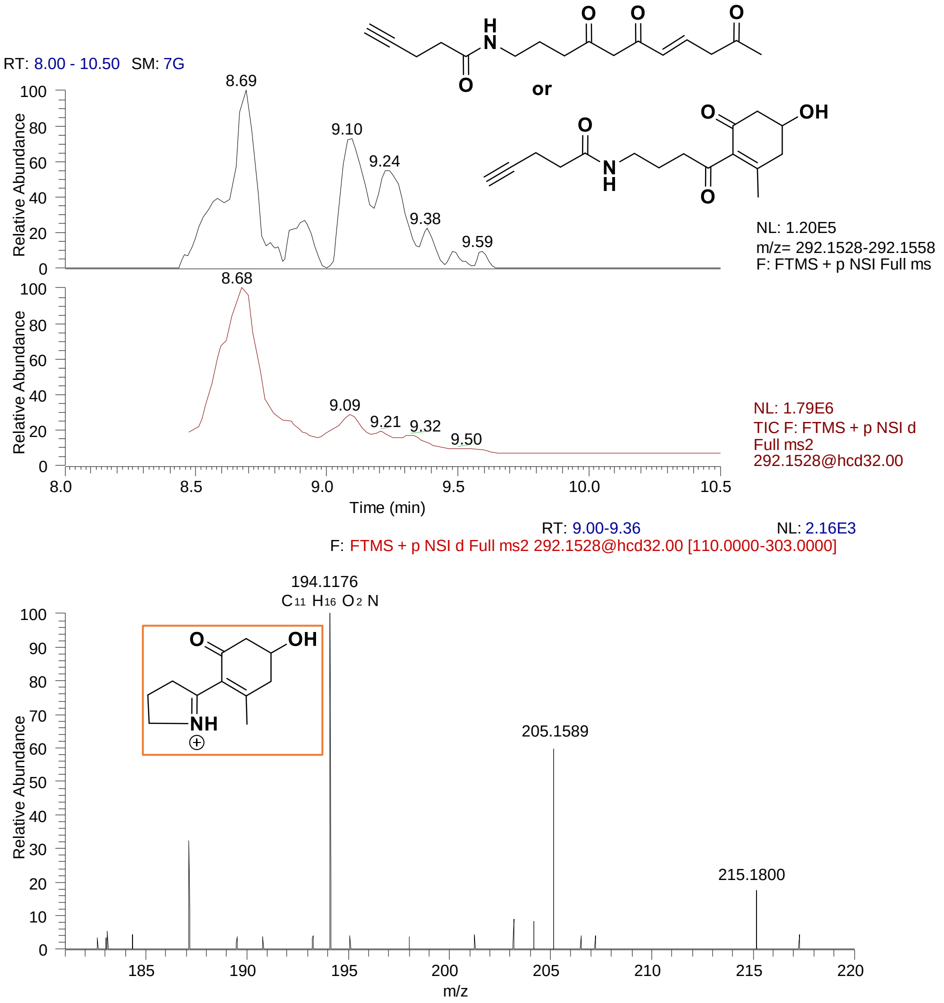

Figure 34S: LC-HRMS analysis (Orbitrap Fusion) of the organic extracts of *S. antibioticus* DSM40725 grown in the presence of **6**: $[M+H]^+$ extracted ion chromatograms (top) and fragmentation (bottom) of a dehydrated tetraketide (with putative fragment structural assignment).

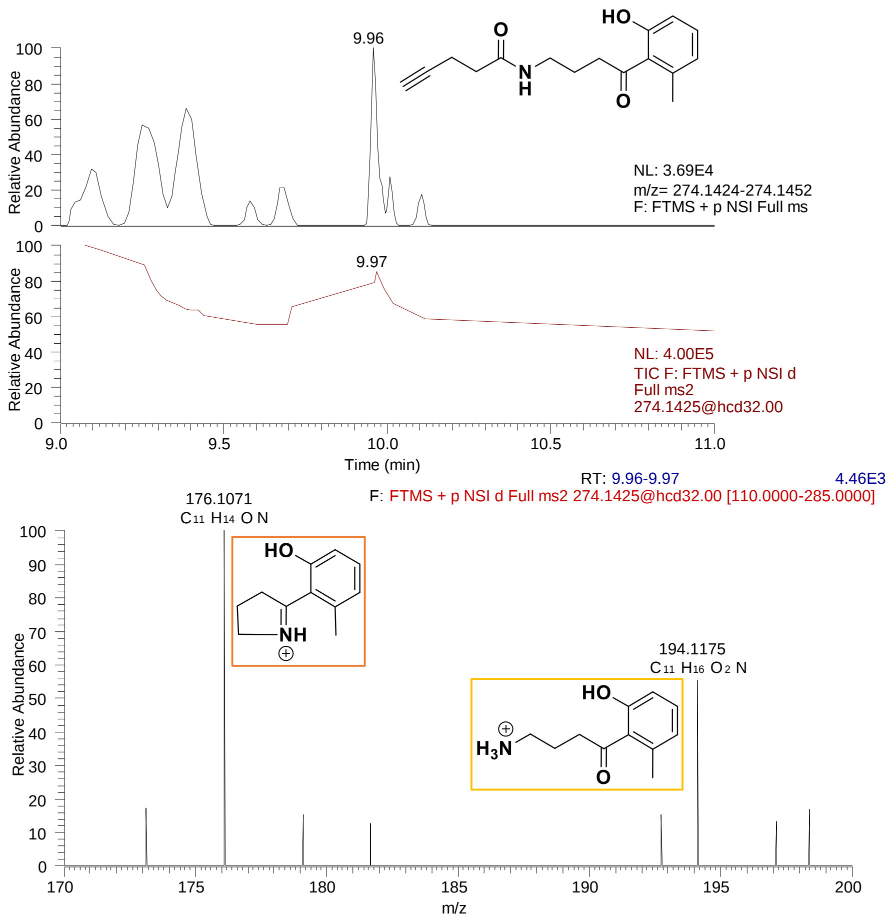

Figure 35S: LC-HRMS analysis (Orbitrap Fusion) of the organic extracts of *S. antibioticus* DSM40725 grown in the presence of **6**: $[M+H]^+$ extracted ion chromatograms (top) and fragmentation (bottom) of a putative aromatized tetraketide (with putative fragment structural assignment). Putative pentaketides were also observed in trace amounts (Table 3S, data not shown).

2.6.3 Intermediate capture by probe 7 (methyl 2-fluoro-3-oxo-6-(pent-4-ynamido) hexanoate)[1b]

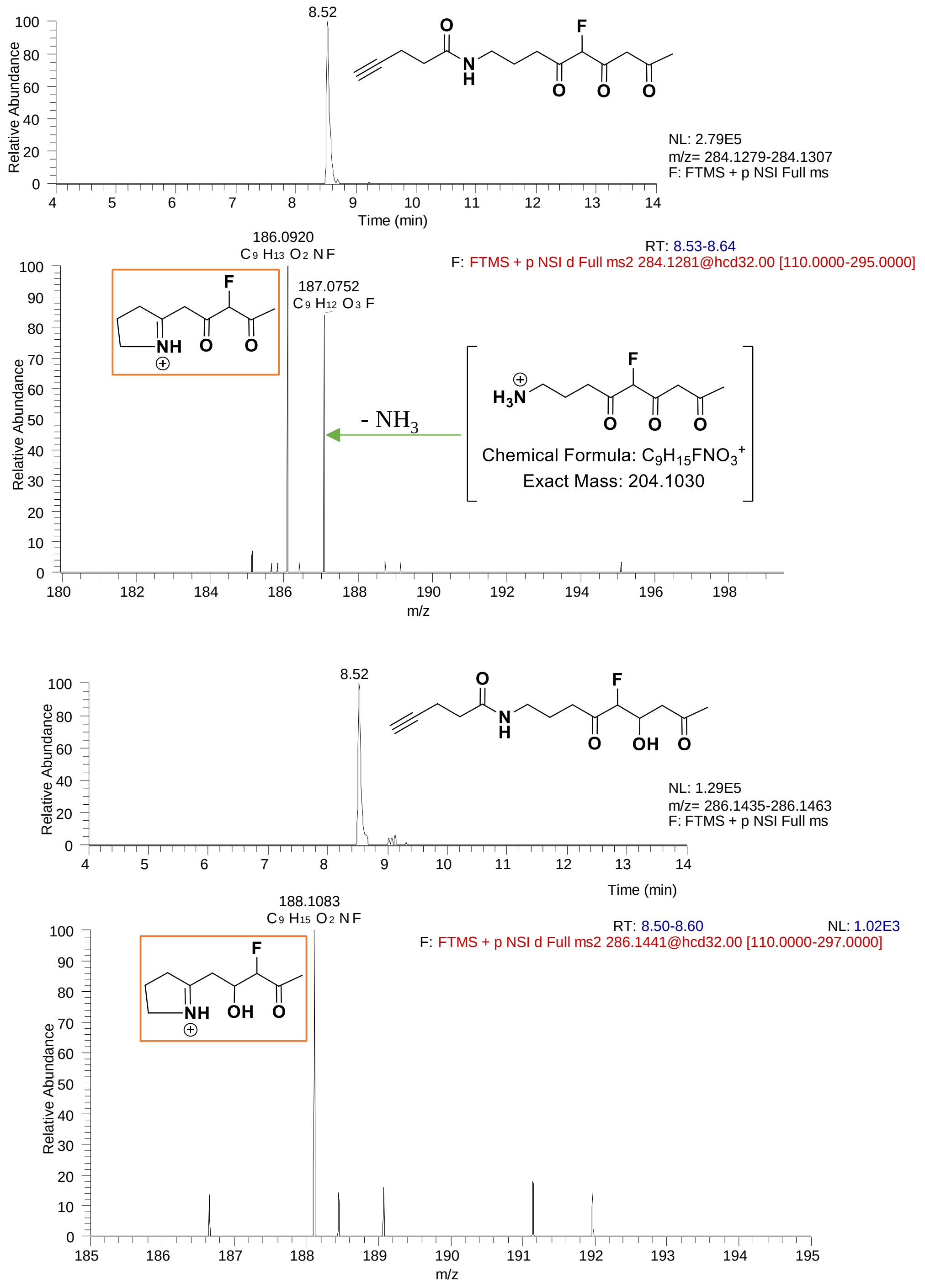

Figure 36S: LC-HRMS analysis (Orbitrap Fusion) of the organic extracts of *S. antibioticus* DSM40725 grown in the presence of **7**: $[M+H]^+$ extracted ion chromatogram and fragmentation of the putative triketides (both with putative fragment structural assignment).

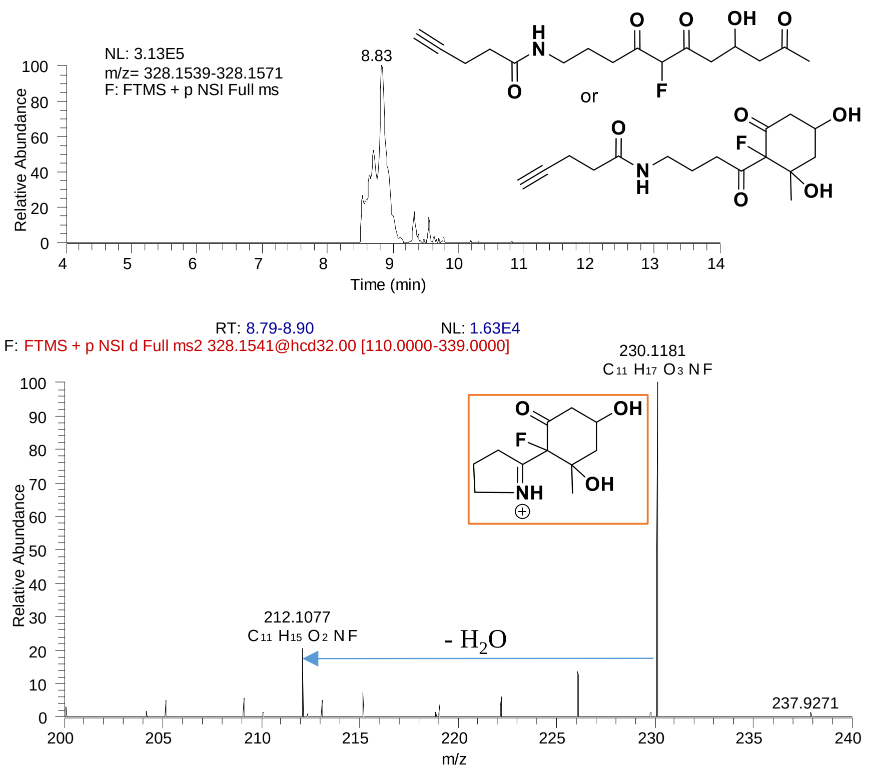

Figure 37S: LC-HRMS analysis (Orbitrap Fusion) of the organic extracts of *S. antibioticus* DSM40725 grown in the presence of **7**: $[M+H]^+$ extracted ion chromatogram (top) and fragmentation (bottom) of a putative hydroxy tetraketide (with putative fragment structural assignment).

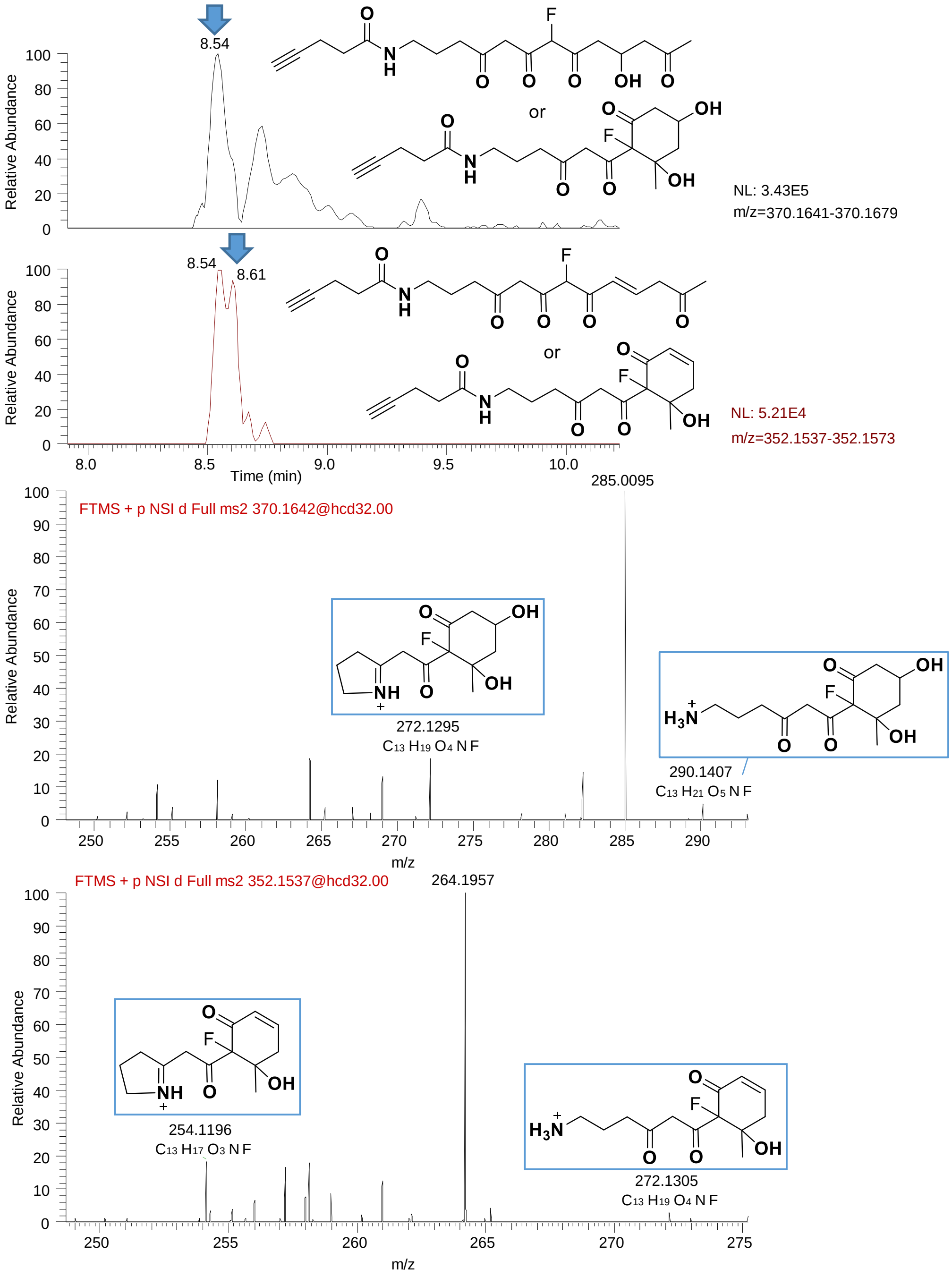

Figure 38S: LC-HRMS analysis (Orbitrap Fusion) of the organic extracts of *S. antibioticus* DSM40725 grown in the presence of **7**: $[M+H]^+$ extracted ion chromatogram (top) and fragmentation (middle and bottom) of putative pentaketides (with putative fragment structural assignment).

3 *In vitro* experiments

3.1 Construction of *E. coli* expressing 6-MSAS and 6-MSAS H958A

The preparation of plasmid pKOS007-109 containing the gene coding for 6-methylsalicylic acid synthase (6-MSAS) has been previously described.[9] For inactivation of the DH domain of 6-MSAS PCR amplifications from pKOS007-109 as a template were carried out in two sections. The left-hand section introduced a mutation into the active site of the DH, converting histidine-958 to an alanine residue between a B*su*36I site and a K*pn*I site using the primers 5'-GGCATCTCCCCTAAGGAGGCCGAGCAGATG-3' and 5'-CTCGGTACCATGGAGAGGAGCGCTGCC CGG -3'. The right-hand section extended the DH domain from a K*pn*I site to a B*am*HI site using primers 5'-CAGCCACCCTCTCCAT GGTACCGAGATCGT-3' and 5'-GATCTTGGGC GGATCCTGCG AGGTGAAGAC-3'. These were digested with K*pn*I/B*su*36I (left) and K*pn*I/B*am*HI (right) and purified via gel extraction. pKOS007-109 was digested with B*su*36I and B*am*HI. The three fragments were ligated using T4 ligase overnight at 16°C using the concentration ratio 1:5:5 (vector:right:left) and the ligation mixture was transformed into competent NovaBlue cells. Colonies were picked and plasmids screened for correct mutation *via* sequencing. Plasmid DHm1 was transformed into *E. coli* BL21 (DE3) alongside pET28a(+)-MtaA[9] or *E. coli* BAP1 in order to yield mutated *holo* synthase.

3.2 6-MSAS and 6-MSAS H958A expression and purification

E. coli BAP1 cells were transformed with plasmid pKOS007-109[9] or plasmid encoding the 6-MSAS H958A mutant and grown in LB medium containing 100 µg/mL carbenicillin to an A_{600}= 0.6 at 37 °C. The temperature was reduced to 20°C and protein expression induced with 0.5 mM IPTG for 16 h. All purification steps were carried out at 4 °C. Cells were harvested by centrifugation (5.000 rpm, 20 minutes, 4 °C) and the pellet resuspended in 7 mL of resuspension buffer (100 mM Tris sulfate buffer, 15% glycerol, 1 mM EDTA, 0.1 mM PMSF, 1 mM benzamidine, 4 mM DTT). Cells were broken in a cell disruptor (20 psi) and cell debris removed by centrifugation (11.000 rpm, 20 minutes, 4 °C). $(NH_4)_2SO_4$ to 22% was added to the supernatant and incubated for an hour at 4 °C before centrifugation (5000 rpm, 30 minutes, 4 °C). $(NH_4)_2SO_4$ to 40% was added and the mixture equilibrated at 4°C for an hour before centrifugation at 11000 x *g* and 4 °C for 15 minutes. The supernatant was decanted and the cell pellet was either flash frozen and stored at -80 °C or further purified *via* gel filtration chromatography.

Gel filtration chromatography was carried out on an ÄKTA Explorer FPLC system with a HiLoad 16/600 Superdex 200 Prep Grade column (Figure 39S and Figure 40S). The ammonium sulfate precipitated protein was dissolved in buffer containing 100 mM Tris sulfate buffer, pH 7.6, 10% glycerol, 5 mM DTT, 1 mM EDTA, 0.1 mM PMSF and 1 mM benzamidine. 1 mL of the protein solution (100 mg/mL) was injected onto the column. The fractions containing the desired peak were combined and concentrated in a PVP-treated spin concentrator with a 100 kDa cut-off (Amicon). The glycerol content of concentrated fractions was adjusted to 50 % to store the protein at -20 °C.

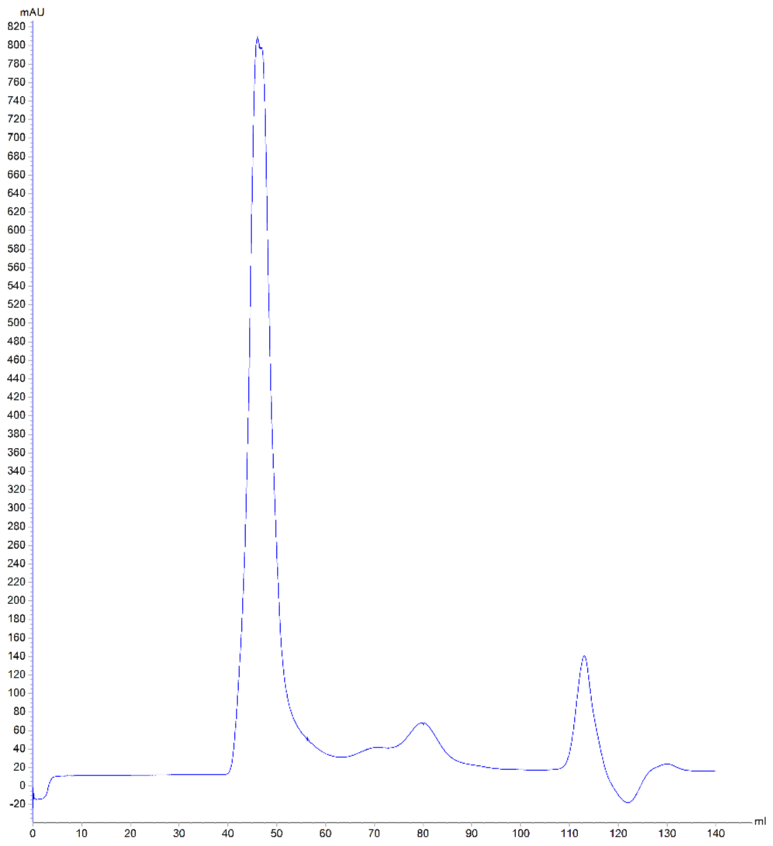

Figure 39S: Representative purification chromatogram of 6-MSAS on a HiLoad 16/600 Superdex 200 Prep Grade gel filtration column.

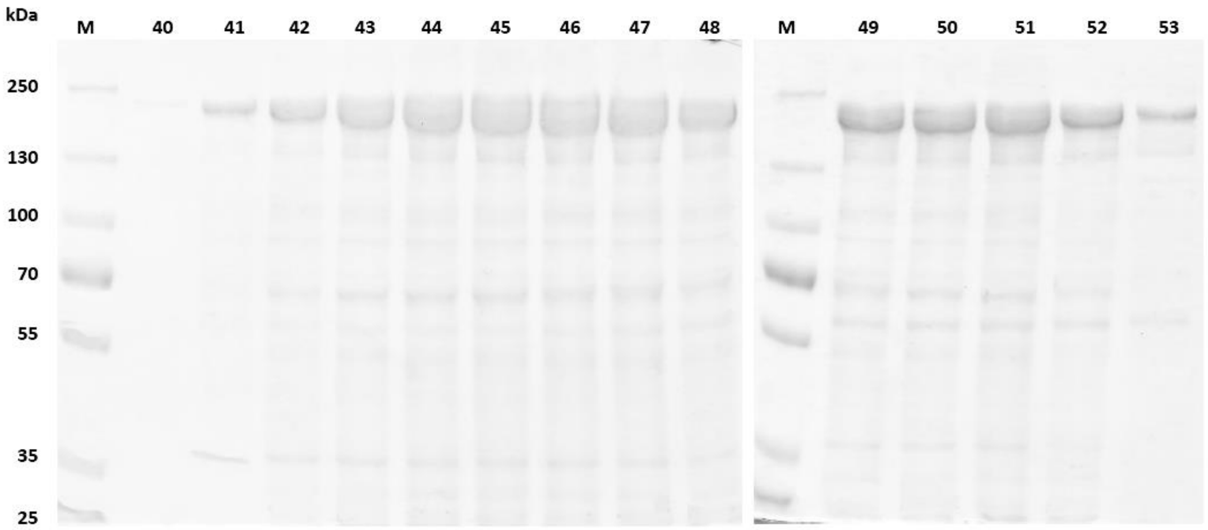

Figure 40S: Coomassie Brilliant Blue stained 8% SDS-PAGE Gel of fractions 40-53 from gel filtration on HiLoad 16/600 Superdex 200 Prep Grade column (Figure 39S).

3.3 6-MSAS and 6-MSAS H958A activity assays

3.3.1 Production of 6-MSA and TAL

6-MSAS was incubated with DTT (10 mM final concentration) for 15 minutes at room temperature before being added to 900 µL of assay buffer (100 mM Tris sulfate buffer, 1 mM EDTA, 15% glycerol, pH 7.6) 10 µL BSA (100 mg/mL), 2 µL NADPH (100 mM), 7 µL acetyl-CoA (30 mM), 7 µL malonyl-CoA (30 mM). The assay mixture was incubated for 16 hours at 25°C before acidification to pH 2 with HCl and extraction with ethyl acetate twice. Extracts were combined, removed *in vacuo* and the residue dissolved in methanol before analysis by TLC (solvent system: benzene/acetone/acetic acid, 10:2:1, v/v/v; Figure 41S), analytical HPLC and LC-MS.

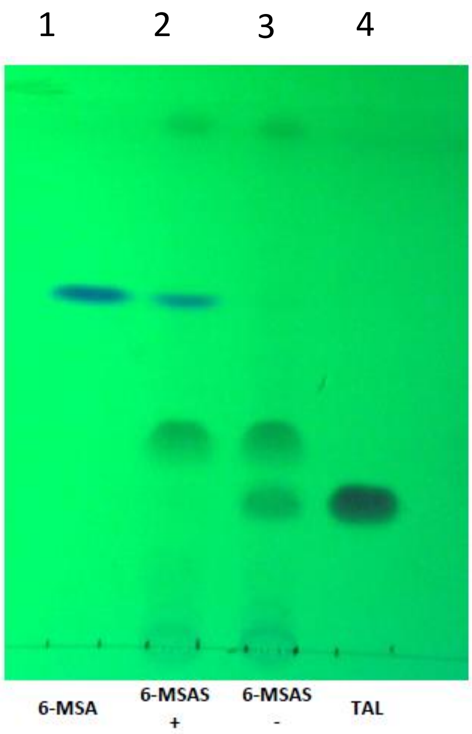

Figure 41S: Formation of 6-MSA and TAL verified by TLC (UV visualisation). Lane 1: authentic 6-MSA; lane 2: 6-MSAS incubated with acetyl CoA and malonyl CoA in the presence (**+**) of NADPH; lane 3: 6-MSAS incubated with acetyl CoA and malonyl CoA without (-) NADPH; lane 4: authentic TAL. In the presence of NADPH 6-MSAS produces 6-MSA (lane 2), in the absence of NADPH no 6-MSA is produced but TAL is found instead (lanes 3 and 4). In similar assay conditions, 6-MSAS H958A does not make 6-MSA in the presence of NADPH and when NADPH is omitted TAL is formed (data not shown).

3.3.2 Ketoreductase activity assay

The ketoreductase activity of recombinant synthases was tested based on a protocol by O'Hare *et al.*[10]. Purified 6-MSAS and 6-MSAS H958A mutant (THm) were tested by adding 50 µL of enzyme solution (10 mg/mL) to 50 µL reaction mixture (100 mM potassium phosphate, pH 7.5, 1.6 mM NADPH and 10 mM trans-1-decalone) in a UV-transparent 96-well plate (NUNC). Reactions were incubated at 25 °C in a Tecan microplate reader and absorbance at 340nm was measured at 30 s intervals for 20 min (Figure 10). NADPH consumption in the presence of the surrogate substrate trans-1-decalone for both enzymes is comparable indicating the surrogate is accepted by both ketoreductases and both are active (Figure 42S).

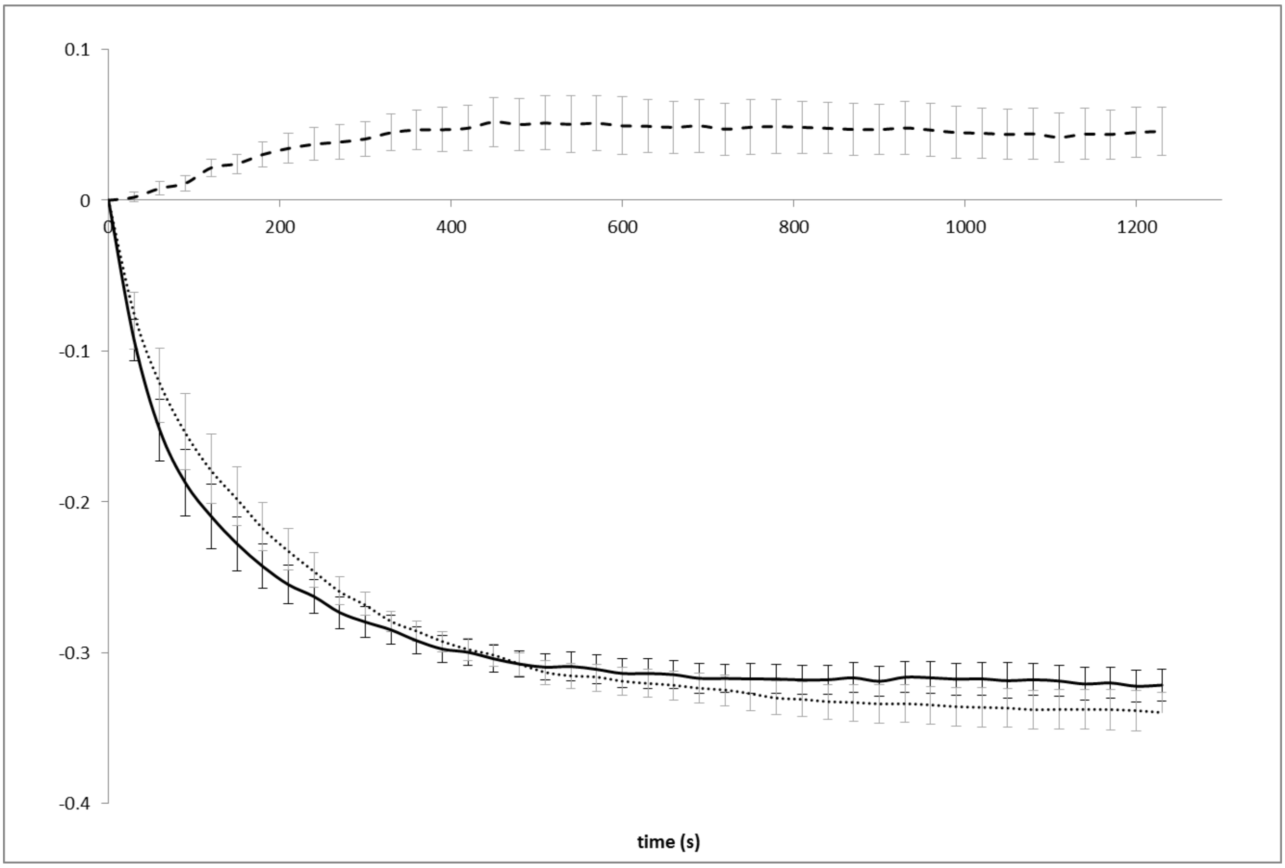

Figure 42S: Ketoreductase activity assay. Dashed Line: control assay containing 50 µL BSA (50 mg/mL) instead of the synthases; solid line: 6-MSAS assay; dotted line: 6-MSAS H958A assay. The measurements, which were carried out in triplicate, were repeated for a total of four times, and the mean ± standard error of the mean (SEM) was considered.

3.3.3 Chain termination assays

Probes **9a-b** were generated *in vitro* by using pig liver esterase (PLE) on methyl ester substrates **4a-b** as previously reported.[1a] **4a-b** were added to standard 6-MSAS assays (section 3.3.1) as previously described.[1a, 9]

3.4 *In vitro* capture of intermediates from 6-MSAS and 6-MSAS H958A *via* probes 9a-b[1a, 9]

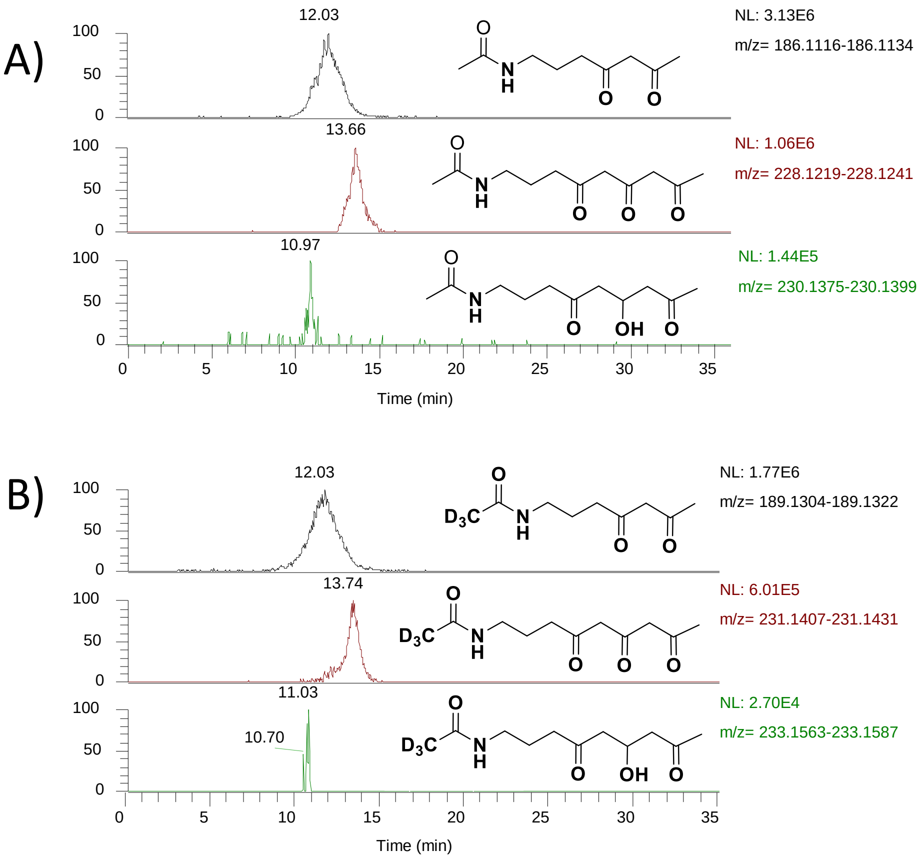

Figure 43S: LC-HRMS analyses of the organic extracts of recombinant 6-MSAS standard assay in the presence of probes **9a-b** (generated *in vitro* by pig liver esterase-catalyzed hydrolysis of **4a-b**):[1a, 9] $[M+H]^+$ extracted ion traces for putative diketides and triketides (unlabeled, **A)**, and deuterated, **B)**). The analyses were performed on an Orbitrap Classic instrument as previously reported.[1a, 1c, 9] These species were not found in control samples (data not shown). In the absence of NADPH reduced triketides were not detected (data not shown).[9]

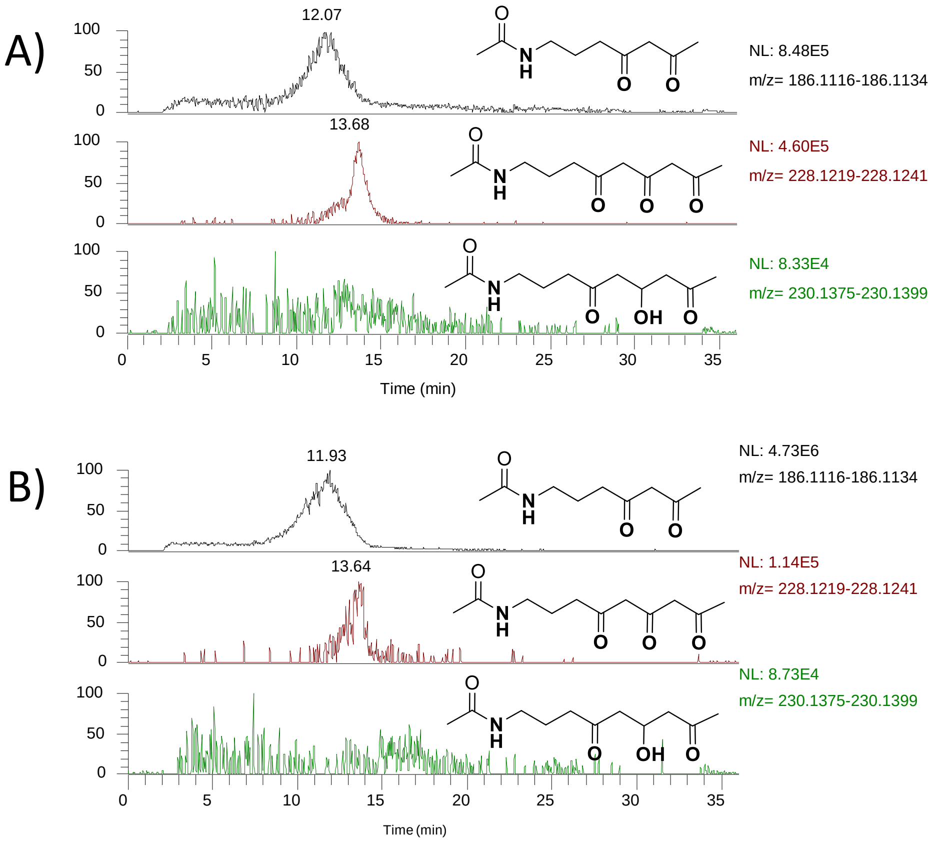

Figure 44S: LC-HRMS analyses of the organic extracts of recombinant 6-MSAS H958A standard assay in the presence of **9a**:[1a, 9] **A)** $[M+H]^+$ extracted ion traces for putative diketides and triketides in the presence of NADPH; **B)** $[M+H]^+$ extracted ion traces for the same species in the absence of NADPH. The analyses were performed on an Orbitrap Classic instrument as previously reported.[1a, 1c, 9] Even in the presence of NADPH the reduced triketides is hardly detectable (**A**), in contrast to what observed for the wild type enzyme (Figure 43S)**:** this is currently under investigation.

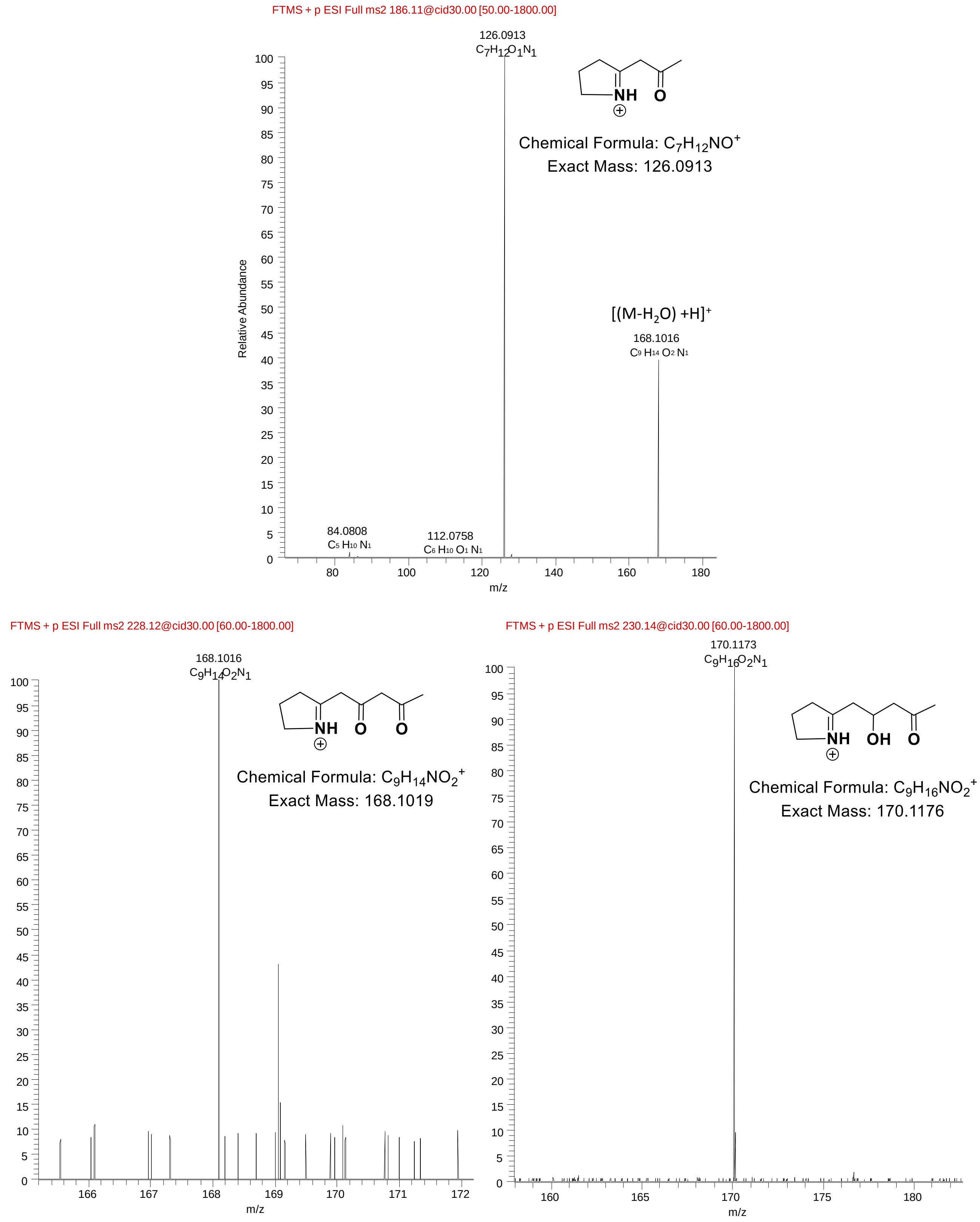

Figure 45S: High resolution fragmentation (Thermo Orbitrap Classic)[1a, 1c, 9] of diketide (top) and triketide species (bottom) observed *in vitro*. Fragmentation of deuterated analogues displayed a similar pattern (data not shown).

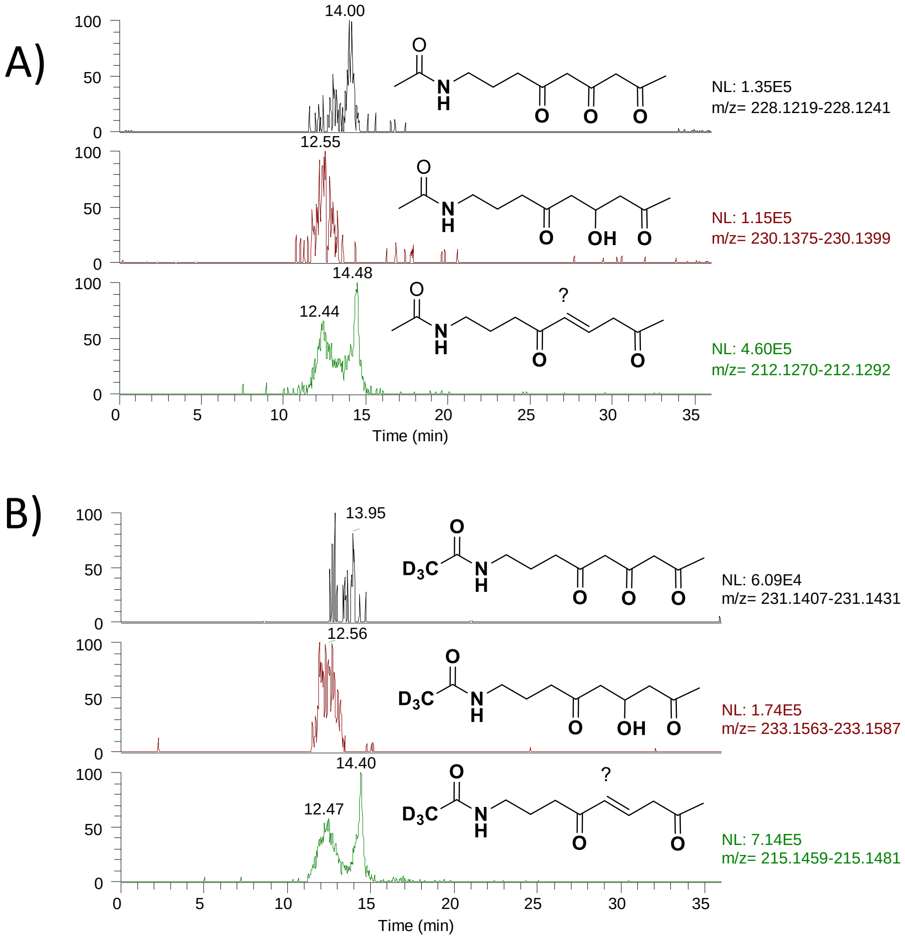

Figure 46S: LC-HRMS analyses of the organic extracts of recombinant 6-MSAS primed with acetoacetyl-CoA in the presence of NADPH and **9a-b**[1a, 9] (no malonyl CoA present). **A)** $[M+H]^+$ extracted ion traces for putative triketides captured by **9a**; **B)** $[M+H]^+$ extracted ion traces for putative triketides captured by **9b**. The analyses were performed on an Orbitrap Classic instrument as previously reported.[1a, 1c, 9] The putative dehydrated triketide species of masses *m/z* 212 and *m/z* 215 were further characterized by ms/ms, giving fragments similar to those of Figure 45S (data not shown).[9]

3.5 Enzymatic dehydration of *N*-(6-hydroxy-4,8-dioxo-nonyl)acetamide 13

100 µL of purified 6-MSAS or 6-MSAS H958A (10 mg/mL) were incubated with DTT (10 mM final concentration) for 15 minutes at room temperature before being added to 400 µL of assay buffer (100 mM Tris sulfate buffer, 1 mM EDTA, 15 % glycerol, pH 7.6) containing 1 mM of *N*-(6-hydroxy-4,8-dioxo-nonyl)acetamide **13.** After 16 h incubation at 25°C the enzymatic mixtures were extracted twice with ethyl acetate; ethyl acetate was removed *in vacuo* and the residue was redissolved in 50 µL methanol and analyzed *via* analytical HPLC or LC-MS. For analytical HPLC analysis (Figure 47S) 10 µL of the extract were injected onto an Atlantis T3 5 µm 2.1x150mm column. The mobile phase consisted of a gradient of water and acetonitrile (HPLC grade, containing 0.1% trifluoroacetic acid, elution gradient starting from 100% H_2O and linearly increasing to 50% ACN over 30 minutes) at a flow rate of 0.3mL/min, with UV detection at 210 nm. No enzyme-catalyzed dehydration of **13** was observed for 6-MSAS (Figure 47S C) or 6-MSAS H958A (Figure 47S D).

A

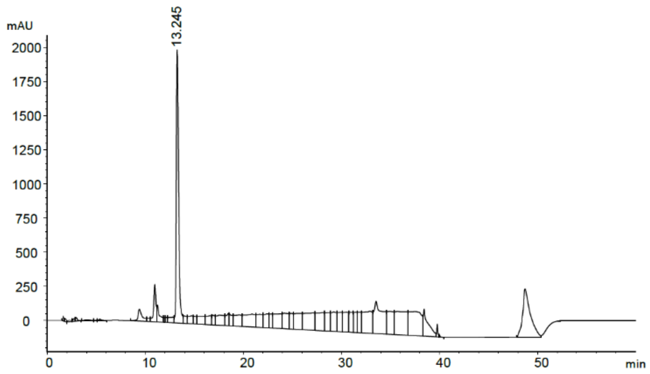

B

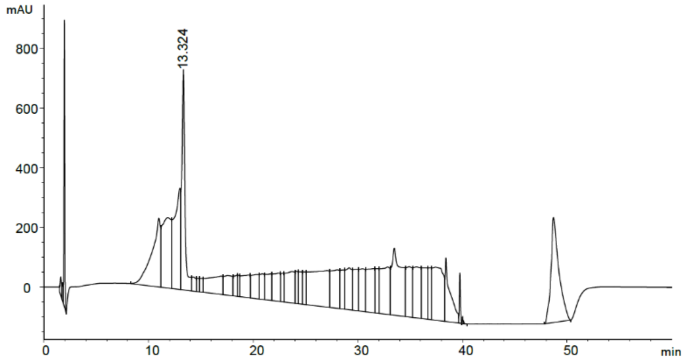

C

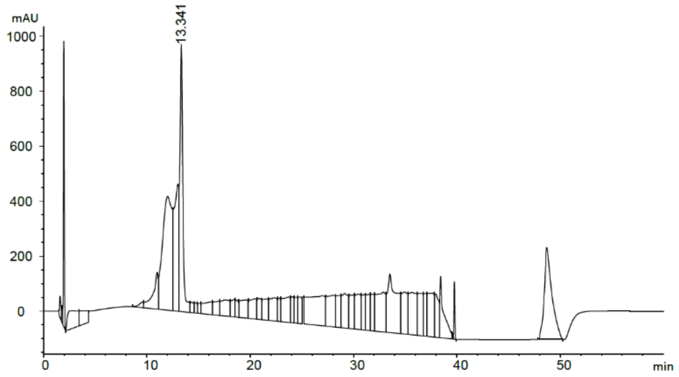

D

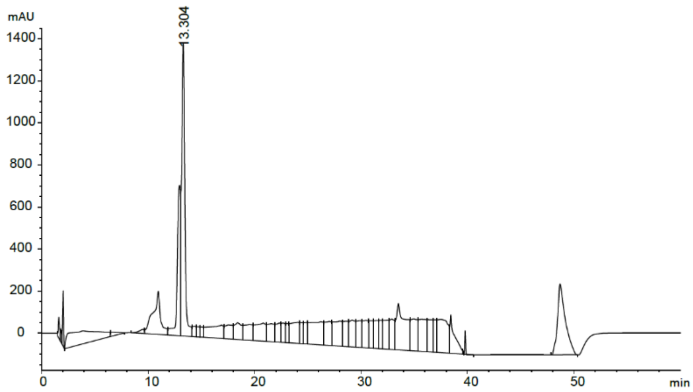

Figure 47S: Analytical HPLC analysis of dehydratase activity assays. **A:** synthetic triketide **13** (in MeOH) ; **B**: synthetic triketide **13** incubated with boiled 6-MSAS; **C**: synthetic triketide **13** incubated with 6-MSAS; **D**: synthetic triketide **13** incubated with 6-MSAS H958A.

3.6 Thioester hydrolase assays

Following the work of Moriguchi *et al.*,[4] the thioester hydrolase activity of 6-MSAS was tested utilizing 6-MSA *N*-acetylcysteamine thioester (**14**, Figure 48S) and 6-MSA *N*-decanoyl thioester (**15**, Figure 49S) as substrates. Substrate **14** or **15** (1mM) was incubated with enzyme (6-MSAS or 6-MSAS H958A) in 100 µL assay buffer (100 mM Tris sulfate buffer, 1 mM EDTA, 15 % glycerol, pH 7.6) for 16 hours at 22 °C. The assay was acidified with HCl, extracted twice with ethyl acetate, the extract concentrated *in vacuo* and redissolved in 50 µL methanol. 10 µL were injected onto an Atlantis T3 5 µm 2.1x150mm analytical HPLC column and/or analyzed by TLC. For analytical HPLC analysis of assays with **14** (Figure), the mobile phase consisted of a gradient of water and acetonitrile (HPLC grade, containing 0.1% trifluoroacetic acid, elution gradient starting from 100% H_2O and linearly increasing to 50% ACN over 30 minutes, at a flow rate of 0.3 mL/min, with UV detection at 280 nm). 6-MSAS did not hydrolyze **14** (Figure 48S C) nor **15** to release 6-MSA (Figure 49S, lane 3).

Figure 48S (in following page)**:** Analytical HPLC analyses of thioester hydrolase activity assays with substrate **14**. **A**: 6-MSA authentic standard (Rt= 19.3 min); **B**: substrate **14** incubated in buffer (Rt= 18.1 min); **C**: substrate **14** incubated with 6-MSAS; **D**: substrate **14** incubated with 6-MSAS H958A. No enzymatic hydrolysis of **14** was observed. Additional HPLC analysis traces of control assays with boiled enzymes and others are available on request.

A

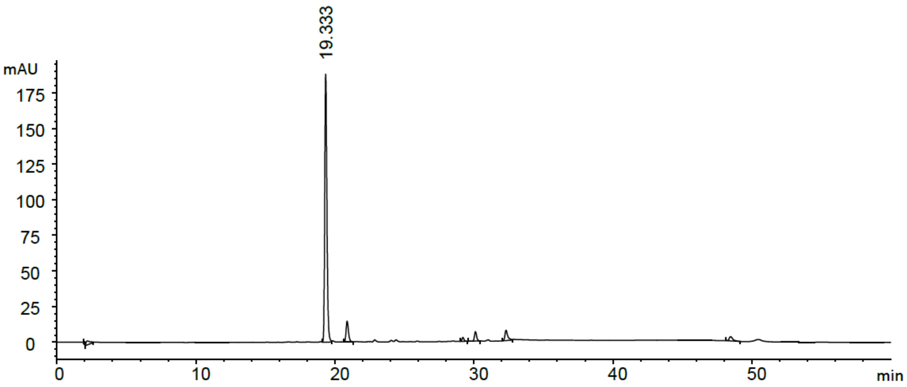
19.333
mAU
175
150
125
100
75
50
25
0
0
10
20
30
40
50
min

B

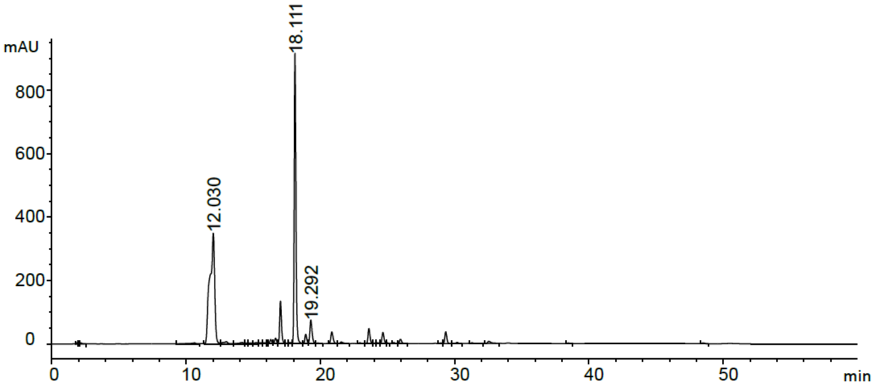
18.111
mAU
800
600
400
200
0
12.030
19.292
0
10
20
30
40
50
min

C

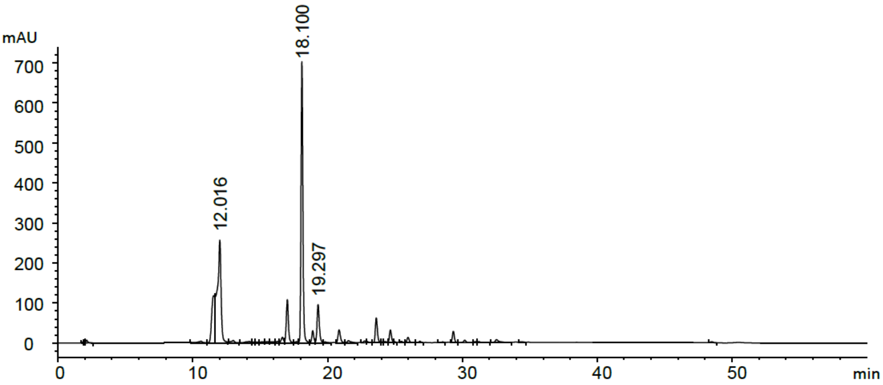
18.100
mAU
700
600
500
400
300
200
100
0
12.016
19.297
0
10
20
30
40
50
min

D

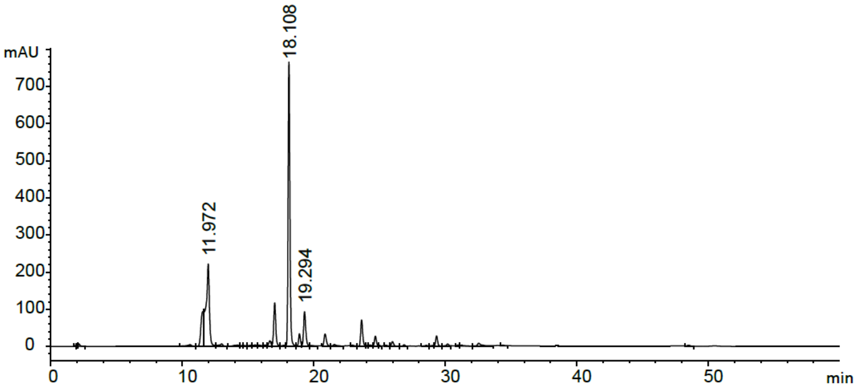
18.108
mAU
700
600
500
400
300
200
100
0
11.972
19.294
0
10
20
30
40
50
min

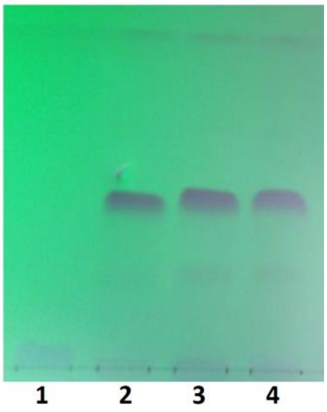

Figure 49S: TLC analyses of thioester hydrolase activity assays with 6-MSA *N*-decanoyl thioester **15** (visualization under UV light). Lane 1: 6-MSA standard (on the base line); lane 2: substrate **15** in buffer; lane 3: substrate **15** incubated with 6-MSAS; lane 4: substrate **15** incubated with 6-MSAS H958A. Additional TLC analysis of control assays with boiled enzymes were comparable to lanes 2-4 (data available on request).

3.7 Comparison of *P. patulum* 6-MSAS to homologues

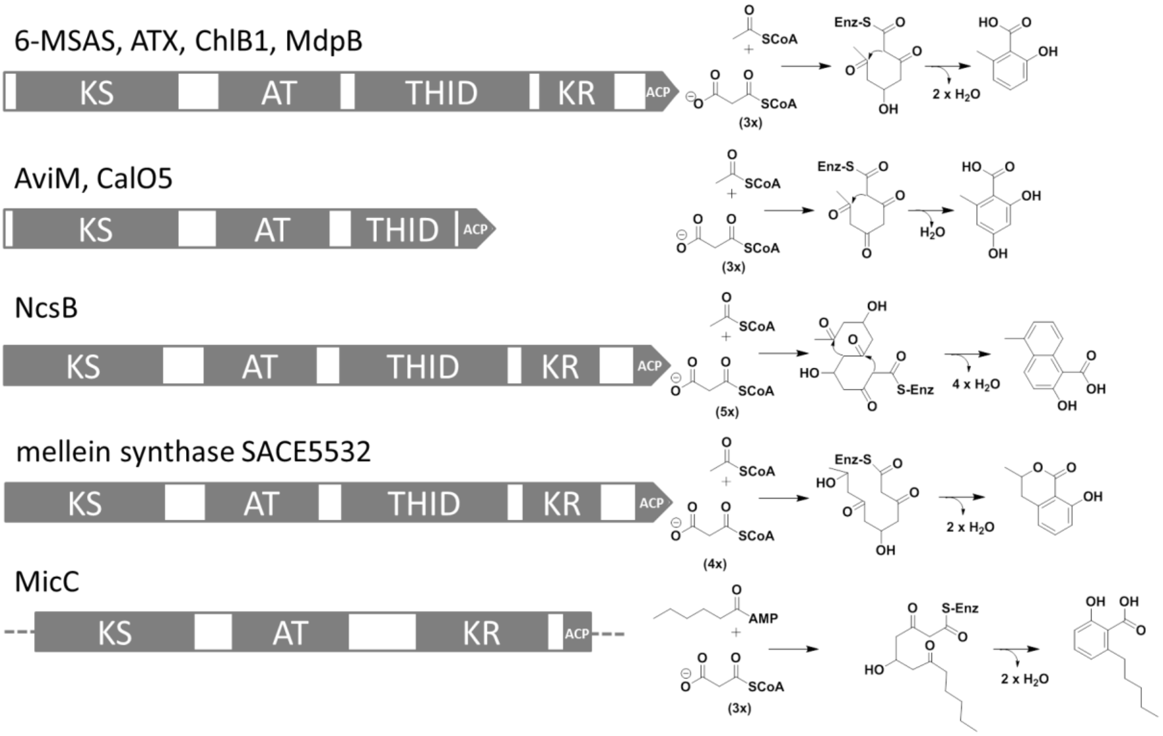

Figure 50S: Overview of iterative PKSs responsible for the production of 6-MSA and related products. The iPKSs herein shown share a highly conserved domain architecture. AviM and CalO5 assemble orsellinic acid and lack a ketoreductase domain. MicC lacks the THID domain that is found in all other 6-MSAS homologues, and its product, 6-pentylsalicylic acid, is immediately transferred to a nonribosomal peptide synthetase.

Table 4S: Summary of iPKSs identity and similarity to 6-MSAS from *P.patulum*

iPKS	Organism	Product	Identity to 6-MSAS from *Penicillium patulum*	Similarity to 6-MSAS from *Penicillium patulum*
**ATX**	*Aspergillus terreus*	6-MSA	62.2%	75.1%
**ChlB1**	*Streptomyces antibioticus*	6-MSA	40.9%	56.3%
**MdpB**	*Actinomadura madurae*	6-MSA	41.4%	55.4%
**AviM**	*Streptomyces viridochromogenes Tü57*	OSA	29.0%	39.3%
**CalO5**	*Micromonospora echinospora*	OSA	30.3%	40.8%
**NcsB**	*Streptomyces carzinostaticus*	2-hydroxyl-5-methyl-NPA	38.6%	54.8%
**mellein synthase SACE5532**	*Parastagonospora nodorum*	mellein	51.2%	68.4%
**MicC**	*Ralstonia solanacearum GMI1000*	6-pentylsalicylic acid	25.8%	38.9%

Figure 51S: Sequence alignment of iPKSs responsible for the production of 6-MSA-based products.

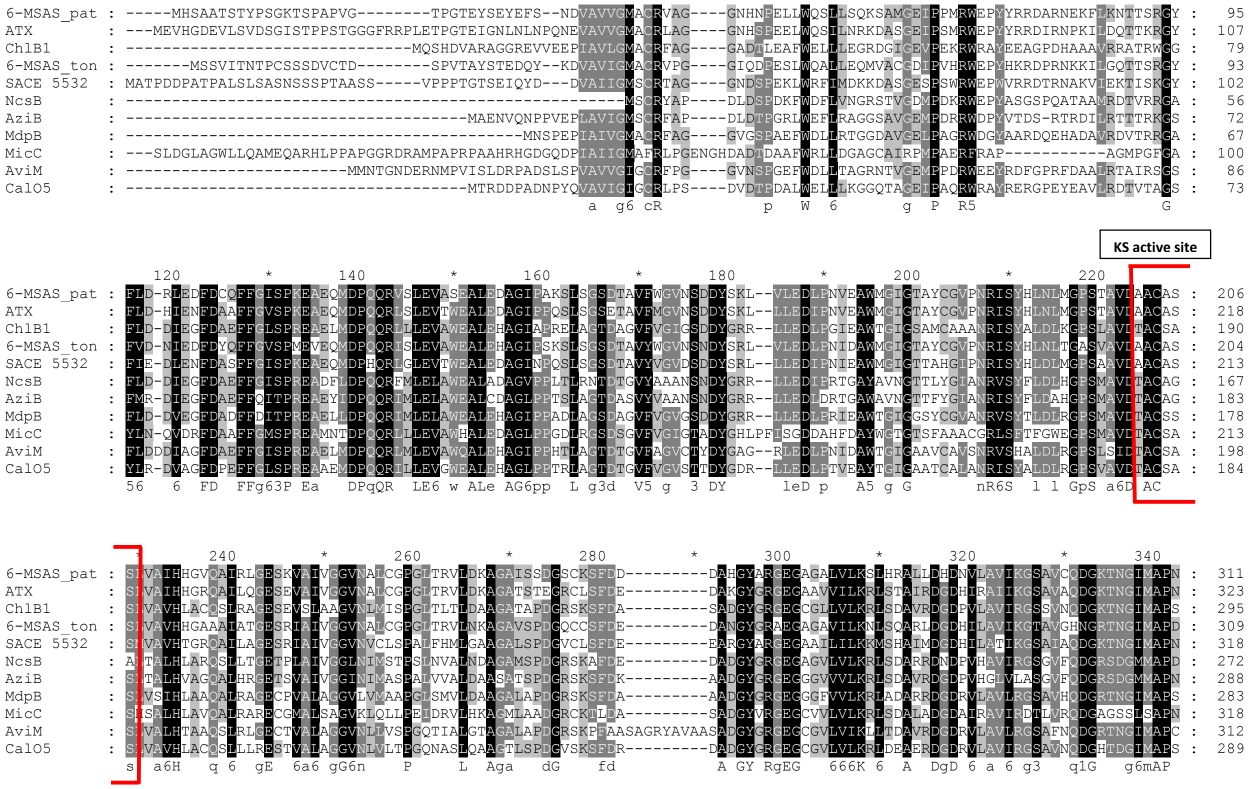

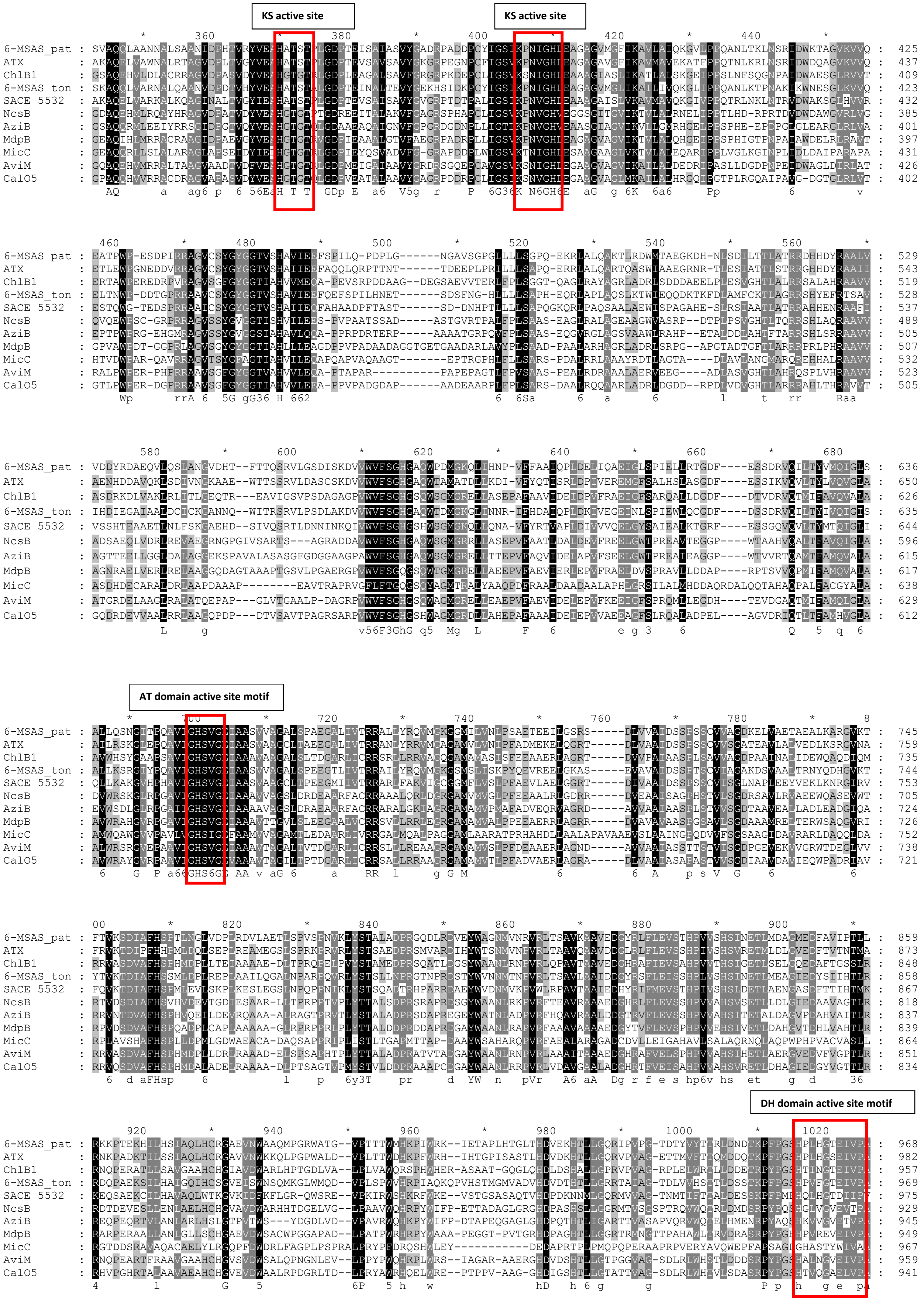
KS active site
KS active site
AT domain active site motif
DH domain active site motif
6-MSAS_pat
ATX
ChlB1
6-MSAS_ton
SACE 5532
NcsB
AziB
MdpB
MicC
AviM
CalO5

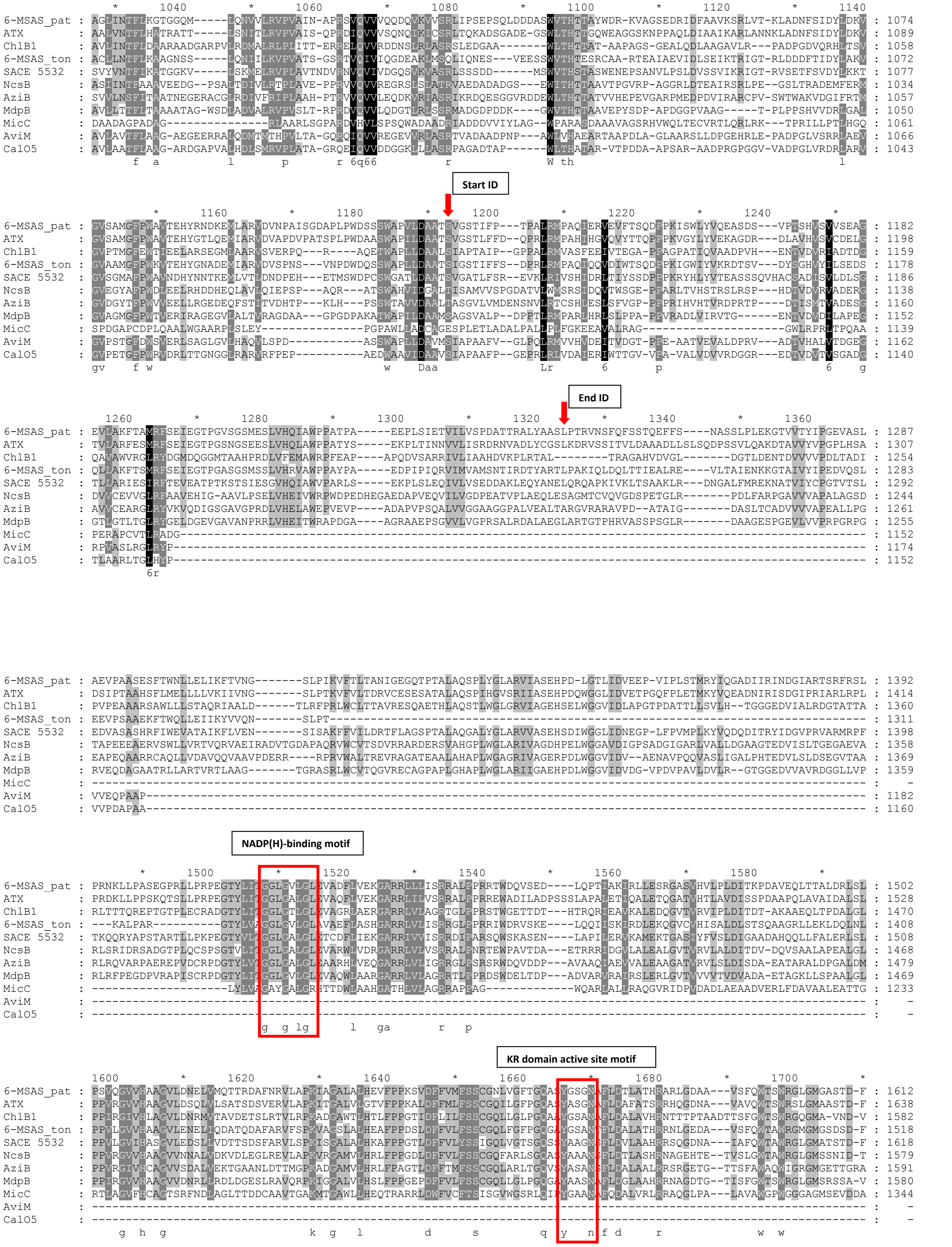
Start ID
End ID
NADP(H)-binding motif
KR domain active site motif

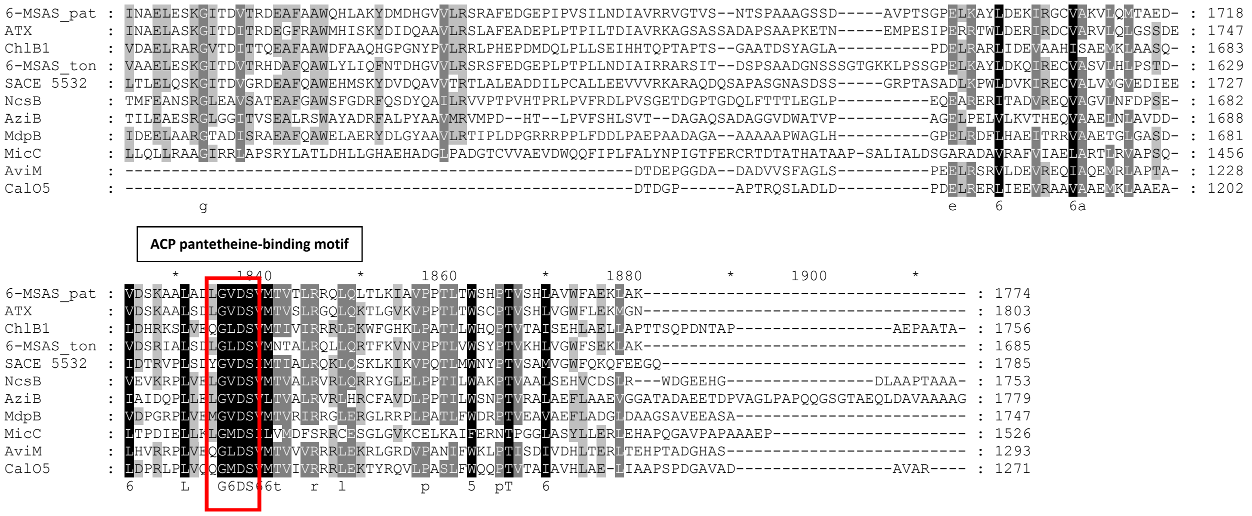

3.8 NMR Spectra of Synthetic compounds

3.8.1 ¹H- NMR of butyl butyl 6-acetamido-3-oxohexanoate (5)

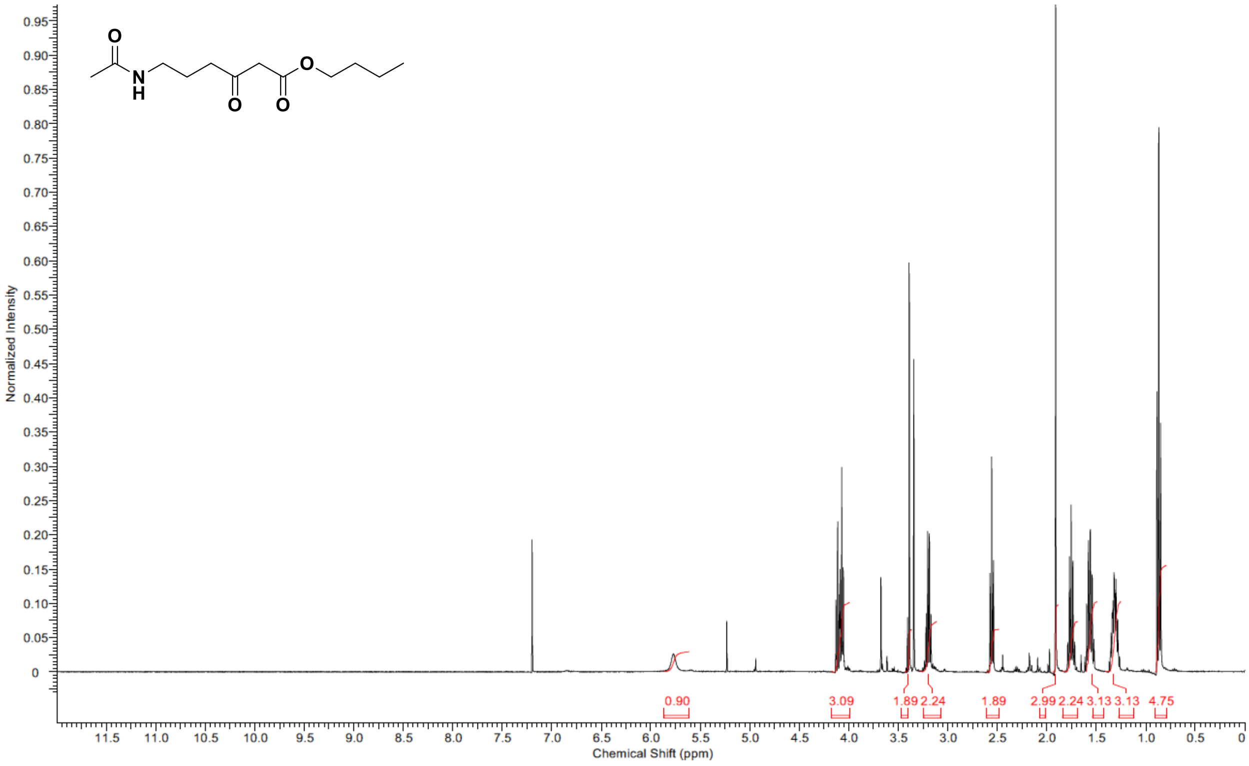

3.8.2 ¹H- and ¹³C- NMR of methyl 2-(2-(3-acetamidopropyl)-4-phenyl-1,3-dioxolan-2-yl)acetate (17)

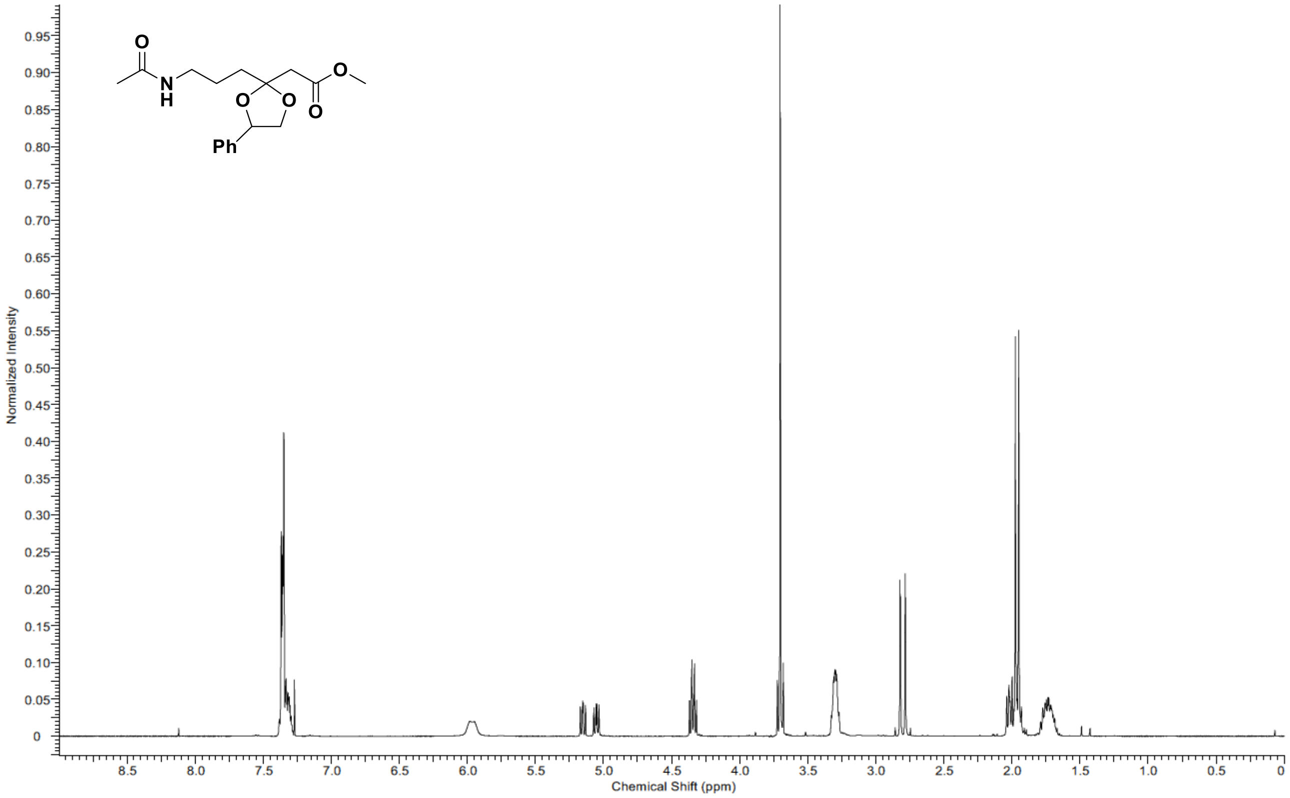

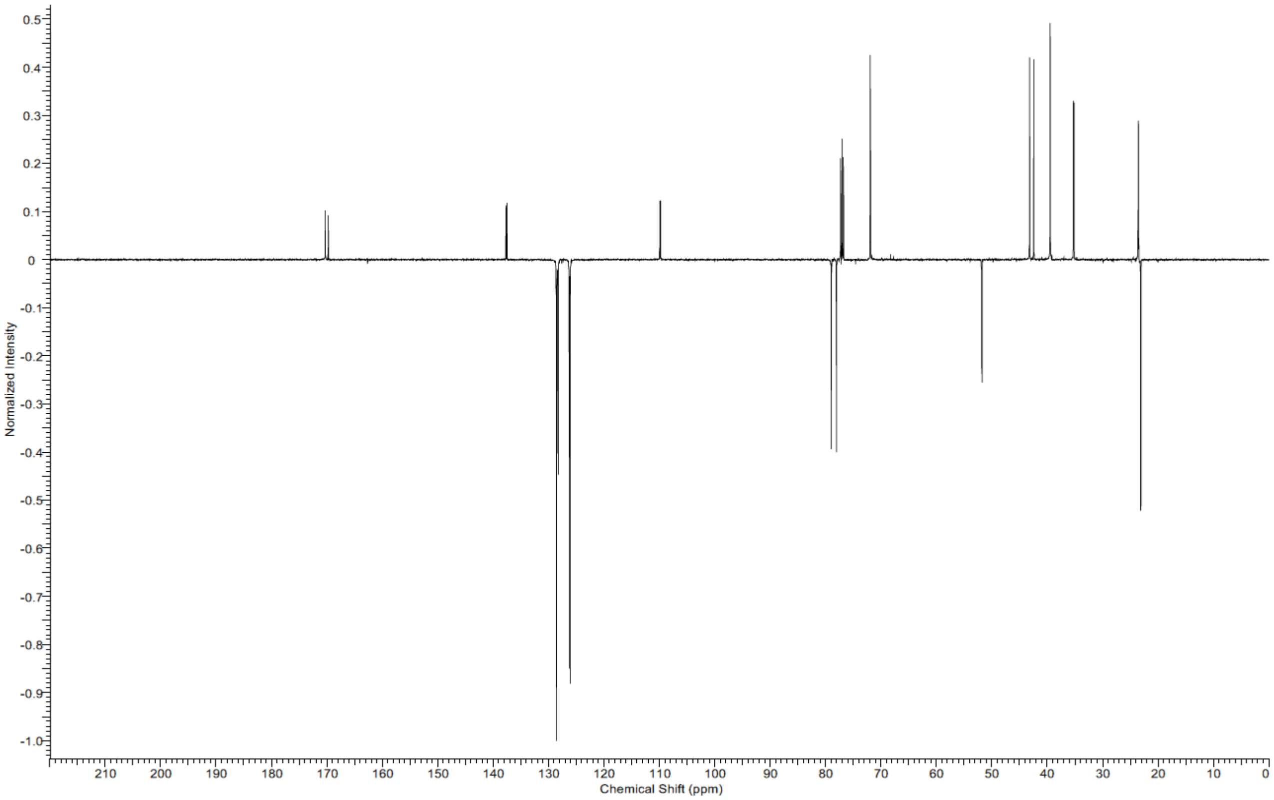

3.8.3 ^{1}H- and ^{13}C- NMR of N-(3-(2-(2-hydroxyethyl)-4-phenyl-1,3-dioxolan-2-yl)propyl)acetamide (18)

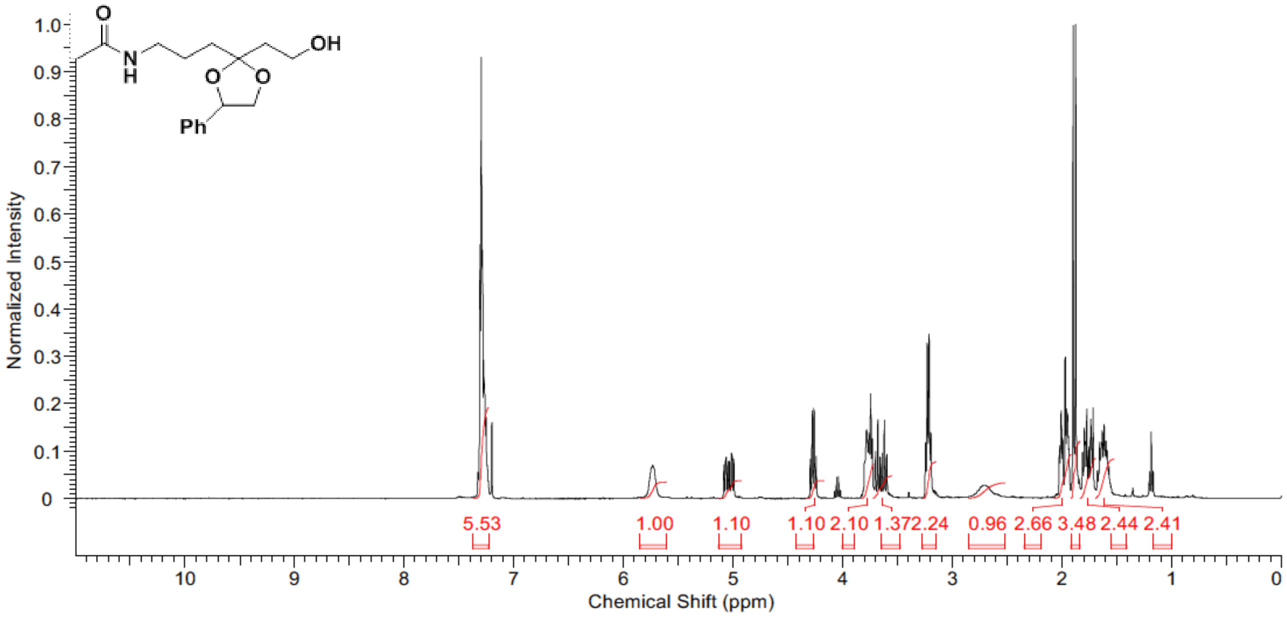

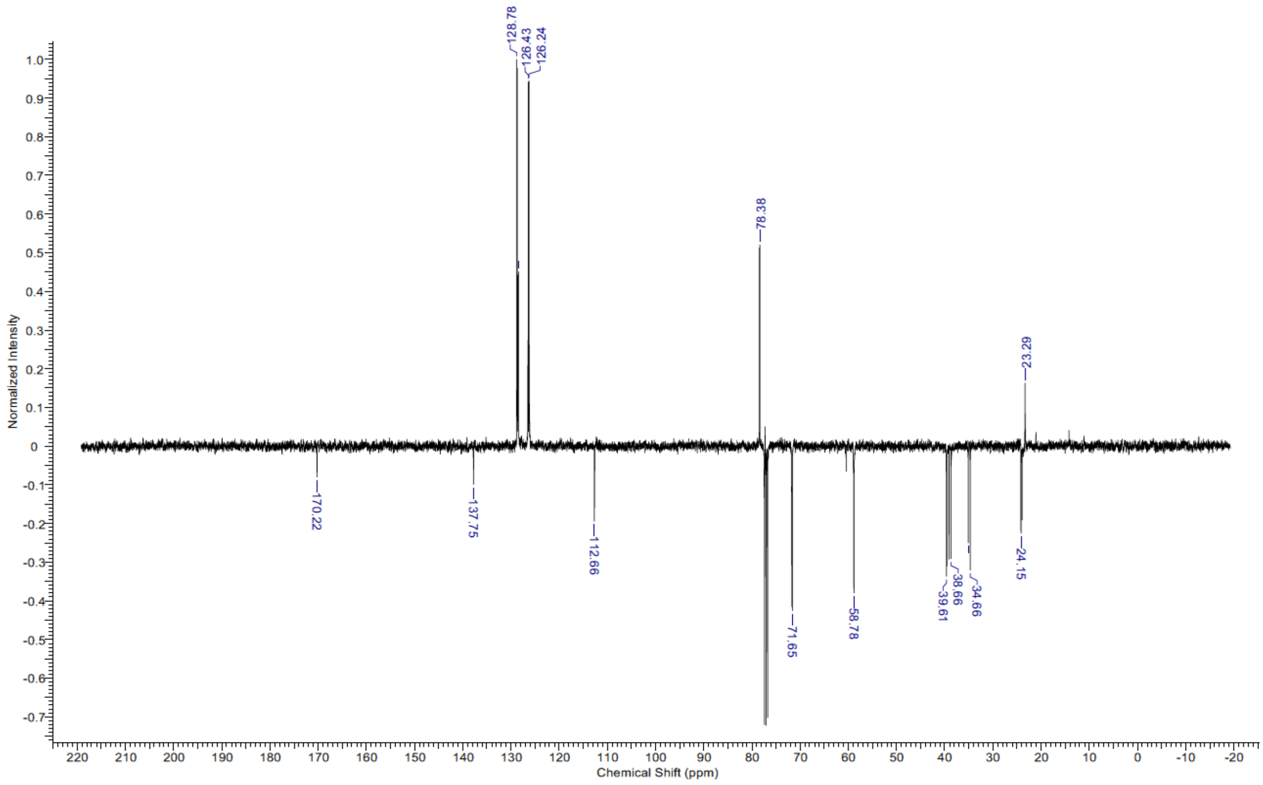

3.8.4 ^{1}H- NMR of *N*-[3-[2-(2-oxoethyl)-4-phenyl-1,3-dioxolan-2 yl]propyl] acetamide (19)

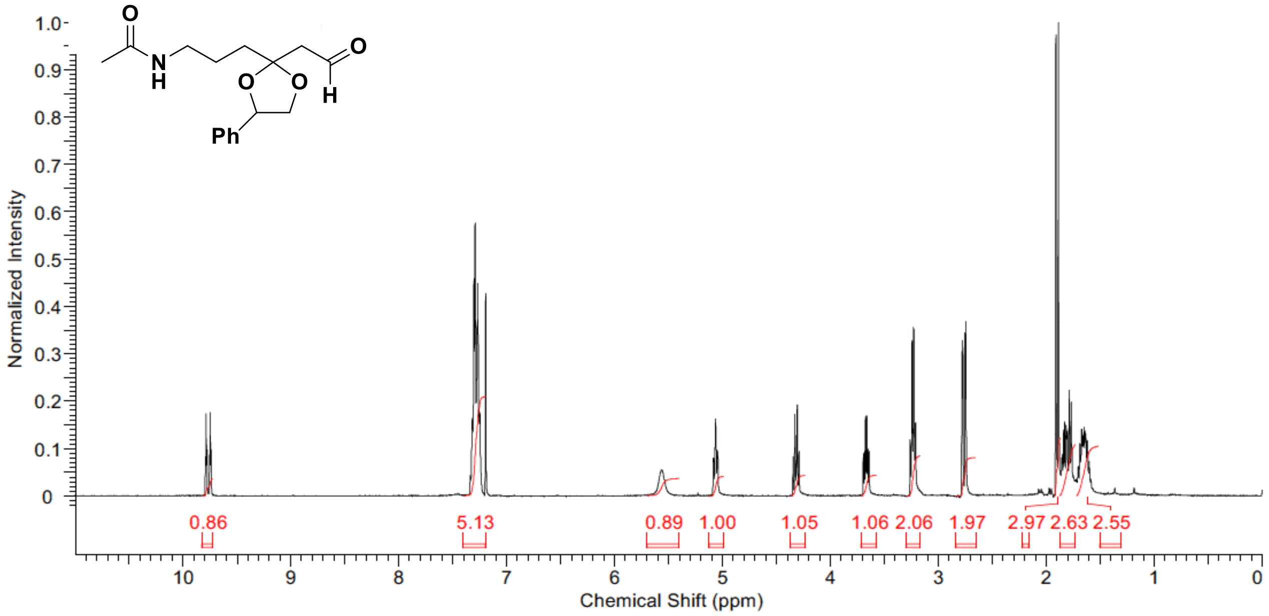

3.8.5 ^{1}H- and ^{13}C- NMR of *N*-(6-hydroxy-4,8-dioxo-nonyl)acetamide (13)

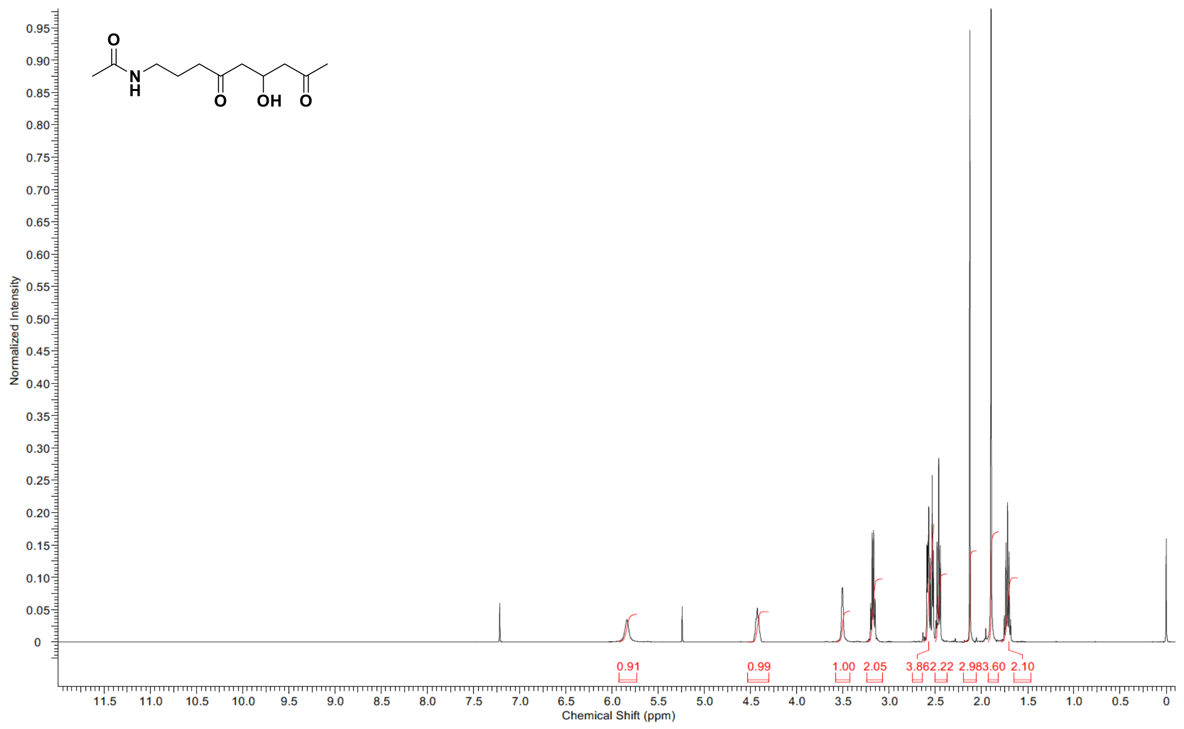

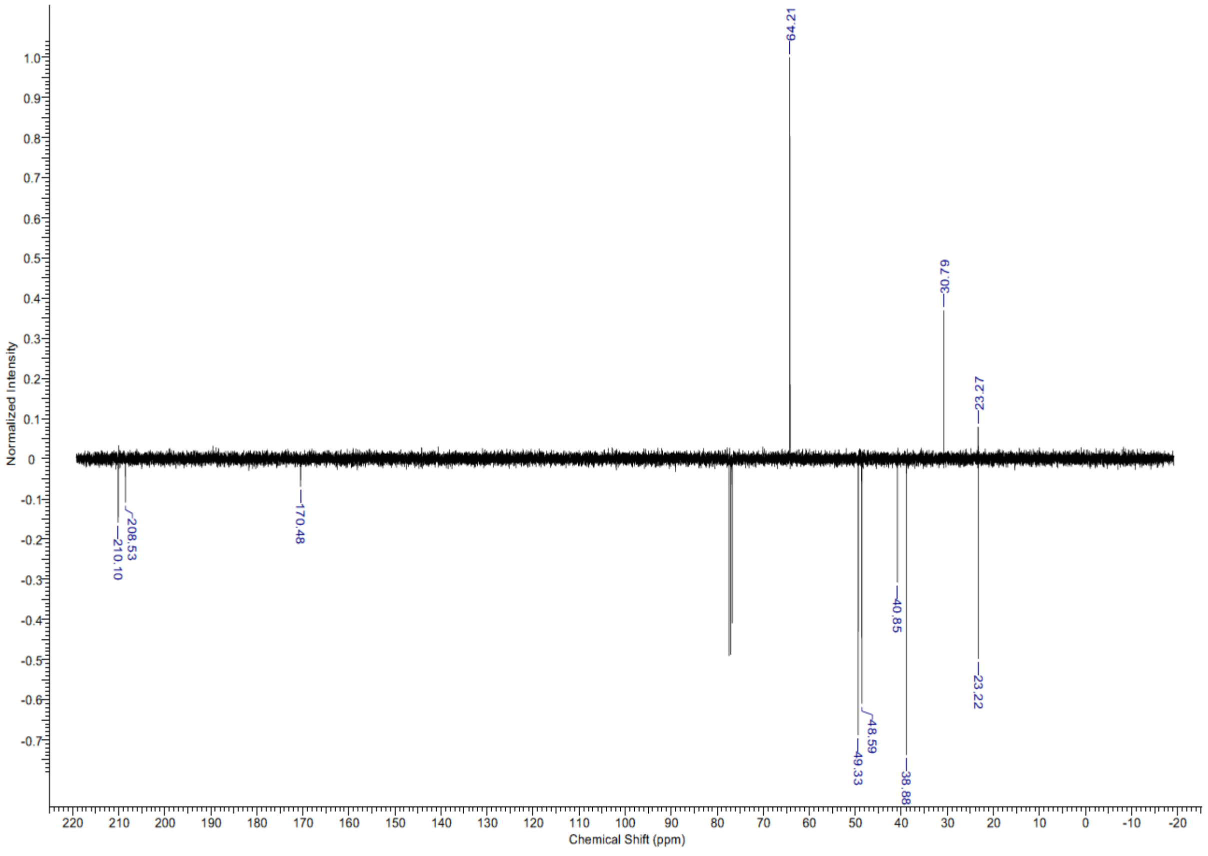

3.8.6 ^{1}H- and ^{13}C-NMR of *N*, *N'*-didecanoylcystamine (23)

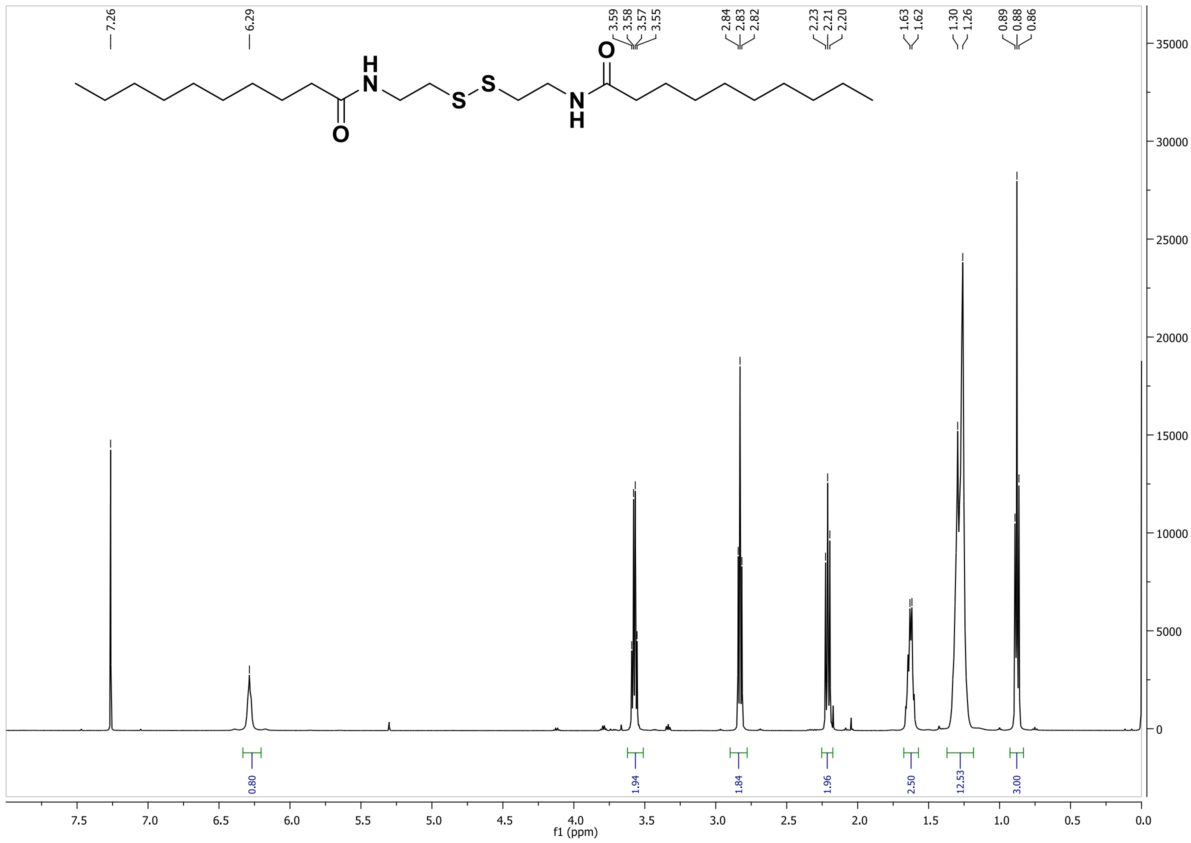

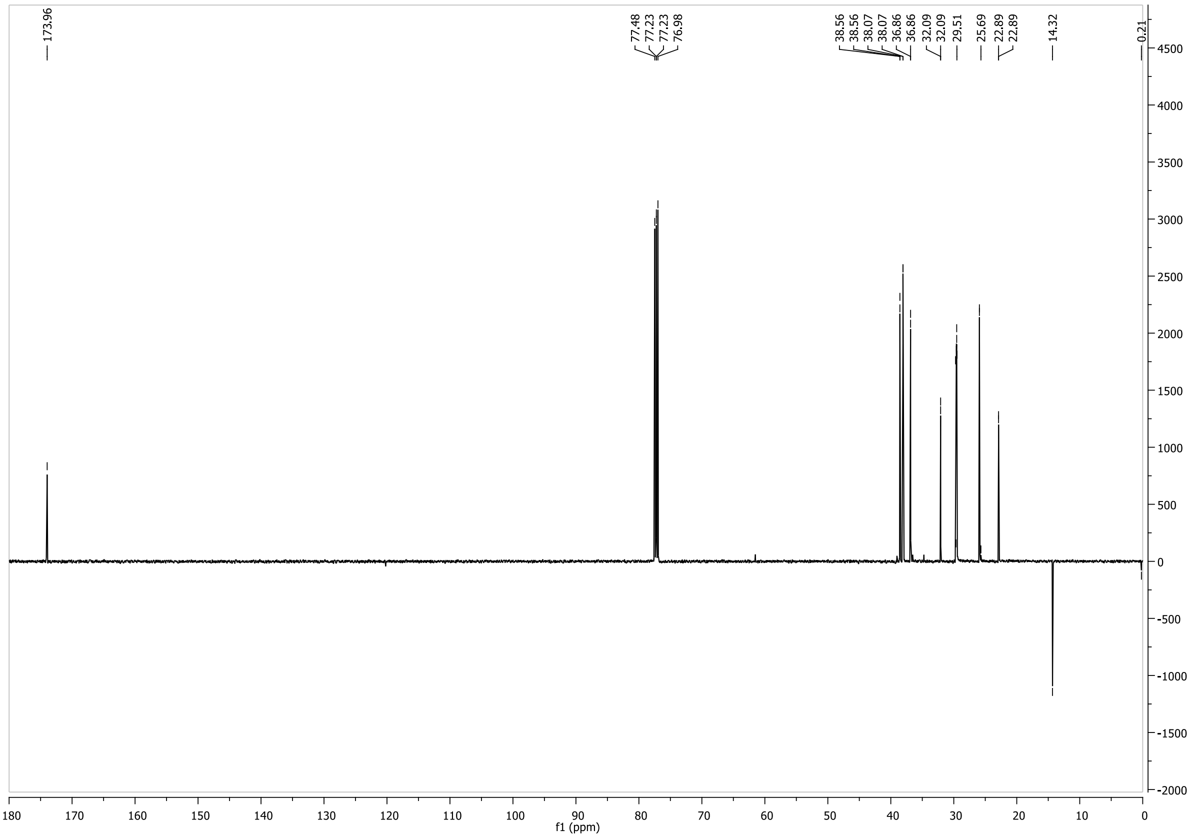

3.8.7 ^{1}H- and ^{13}C-NMR of *N*-decanoylcysteamine (24)

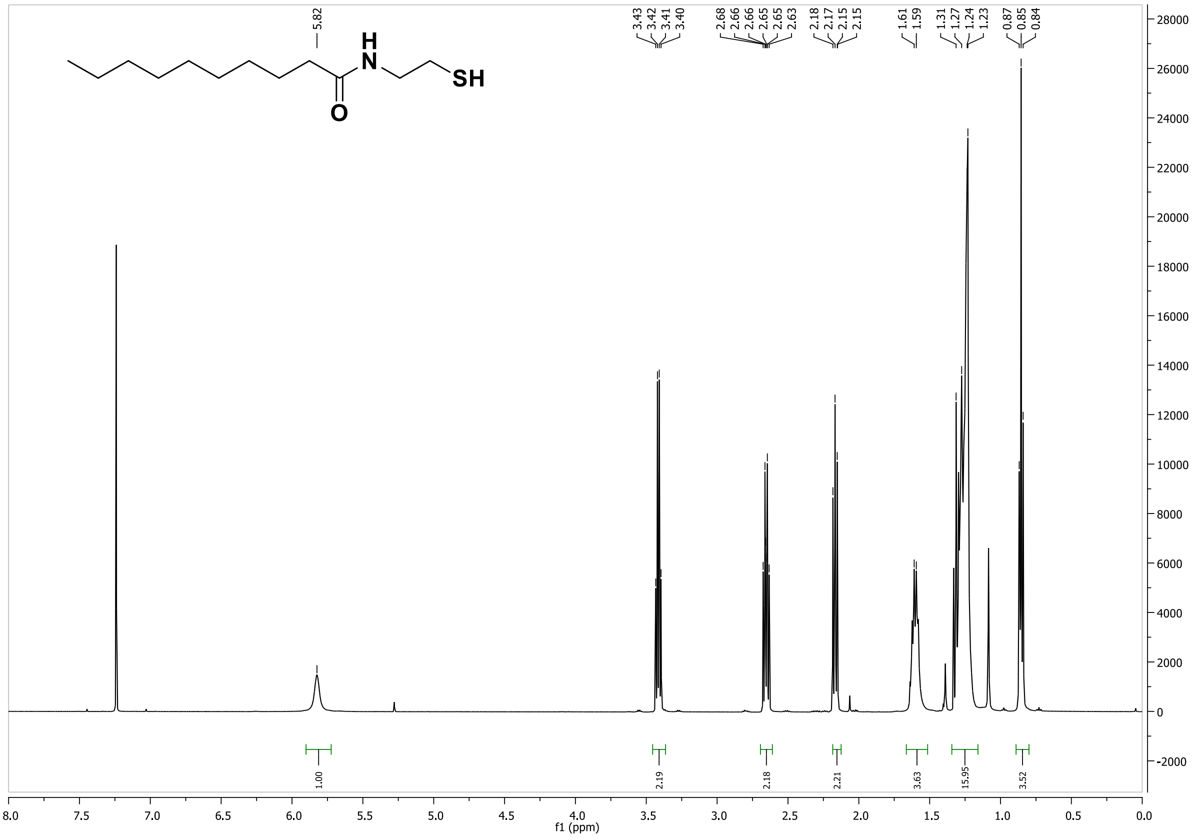

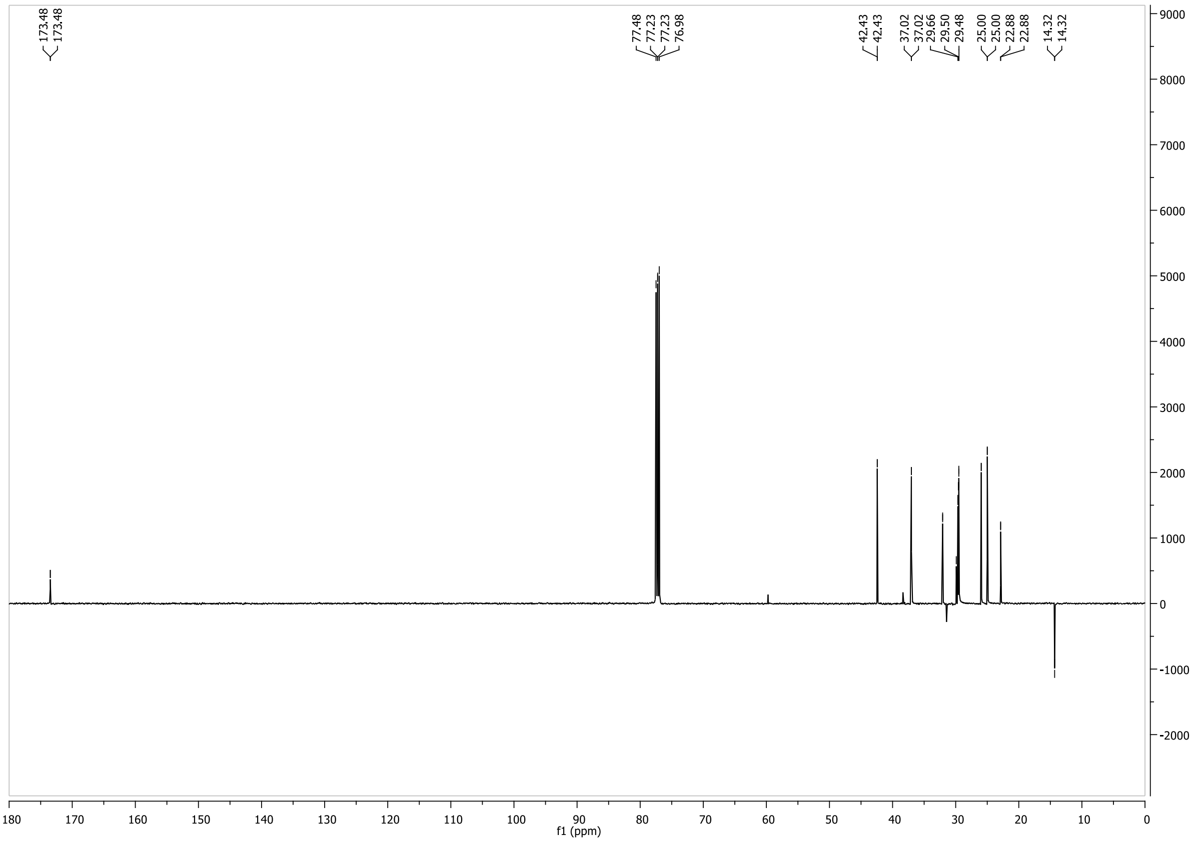

3.8.8 ^{1}H- and ^{13}C-NMR of 6-MSA *N*-decanoylcysteamine thioester (15)

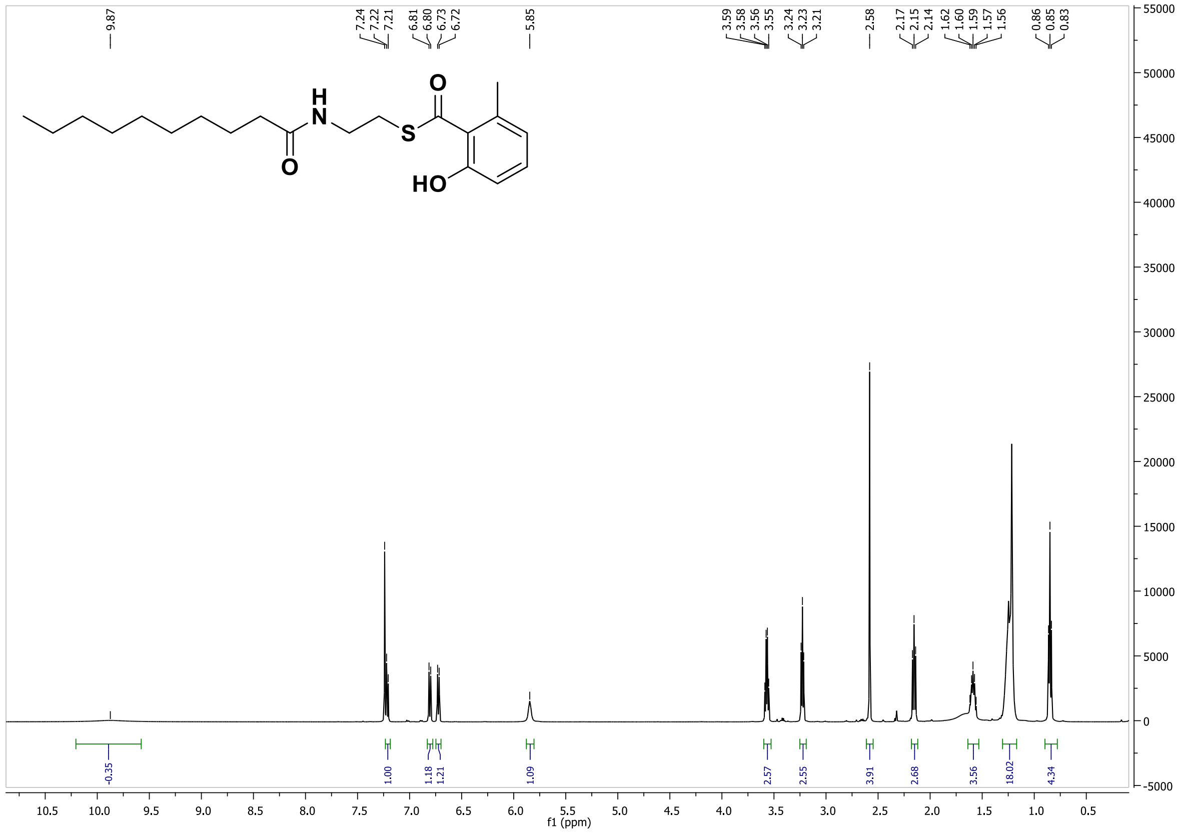

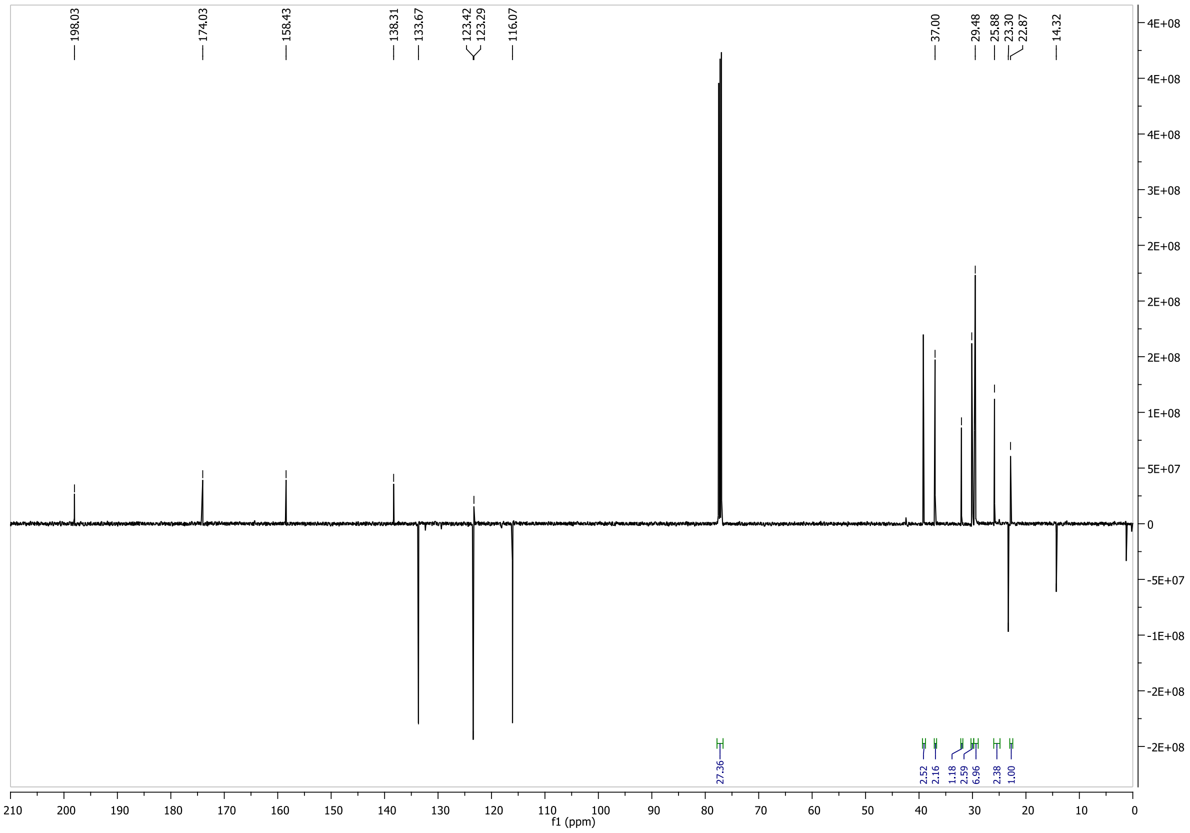

4 References

[1] a) M. Tosin, L. Betancor, E. Stephens, W. M. Ariel Li, J. B. Spencer, P. F. Leadlay, *ChemBioChem* **2010**, *11*, 539-546; b) E. Riva, I. Wilkening, S. Gazzola, W. M. Li, L. Smith, P. F. Leadlay, M. Tosin, *Angew. Chem. Int. Ed. Engl.* **2014**, *53*, 11944-11949; c) M. Tosin, Y. Demydchuk, J. S. Parascandolo, C. B. Per, F. J. Leeper, P. F. Leadlay, *Chem. Commun.* **2011**, *47*, 3460-3462.

[2] F. P. Silverman, P. D. Petracek, D. F. Heiman, C. M. Fledderman, P. Warrior, *J. Agric. Food. Chem.* **2005**, *53*, 9775-9780.

[3] F. Mazzini, F. Galli, P. Salvadori, *Eur. J. Org. Chem.* **2006**, *2006*, 5588-5593.

[4] T. Moriguchi, Y. Kezuka, T. Nonaka, Y. Ebizuka, I. Fujii, *J. Biol. Chem.* **2010**, *285*, 15637-15643.

[5] E. D. Goddard-Borger, M. B. Tropak, S. Yonekawa, C. Tysoe, D. J. Mahuran, S. G. Withers, *J. Med. Chem.* **2012**, *55*, 2737-2745.

[6] B. McCaughan, G. Kay, R. M. Knott, D. Cairns, *Bioorg. Med. Chem. Lett.* **2008**, *18*, 1716-1719.

[7] L. Tran, M. Tosin, J. B. Spencer, P. F. Leadlay, K. J. Weissman, *ChemBioChem* **2008**, *9*, 905-915.

[8] B. Kusebauch, N. Brendel, H. Kirchner, H.-M. Dahse, C. Hertweck, *ChemBioChem* **2011**, *12*, 2284-2288.

[9] H. K. Potter, *PhD Thesis*, University of Cambridge **2011**.

[10] H. M. O'Hare, A. Baerga-Ortiz, B. Popovic, J. B. Spencer, P. F. Leadlay, *Chem. Biol.* **2006**, *13*, 287-296.
